# New Benzimidazole-Triazole Derivatives as Topoisomerase I Inhibitors: Design, Synthesis, Anticancer Screening, and Molecular Modeling Studies

**DOI:** 10.1021/acsomega.3c10345

**Published:** 2024-03-06

**Authors:** Ulviye Acar Çevik, Betül Kaya, Ismail Celik, Mithun Rudrapal, Gourav Rakshit, Arzu Karayel, Serkan Levent, Derya Osmaniye, Begüm Nurpelin Sağlık Özkan, Merve Baysal, Özlem Atlı Ekliog̈lu, Yusuf Özkay, Zafer Asım Kaplancıklı

**Affiliations:** † Department of Pharmaceutical Chemistry, Faculty of Pharmacy, Anadolu University, Eski̧ehir 26470, Turkey; ‡ Department of Pharmaceutical Chemistry, Faculty of Pharmacy, Zonguldak Bülent Ecevit University, Zonguldak 67100, Turkey; § Department of Pharmaceutical Chemistry, Faculty of Pharmacy, Erciyes University, Kayseri 38039, Turkey; ∥ Department of Pharmaceutical Sciences, School of Biotechnology and Pharmaceutical Sciences, Vignan’s Foundation for Science, Technology & Research (Deemed to Be University), Guntur 522213, India; ⊥ Department of Pharmaceutical Sciences & Technology, Birla Institute of Technology, Ranchi 835215, India; # Department of Physics, Faculty of Arts and Science, Hitit University, Çorum 19030, Turkey; ∇ Department of Pharmaceutical Toxicology, Faculty of Pharmacy, Anadolu University, Eski̧ehir 26470, Turkey

## Abstract

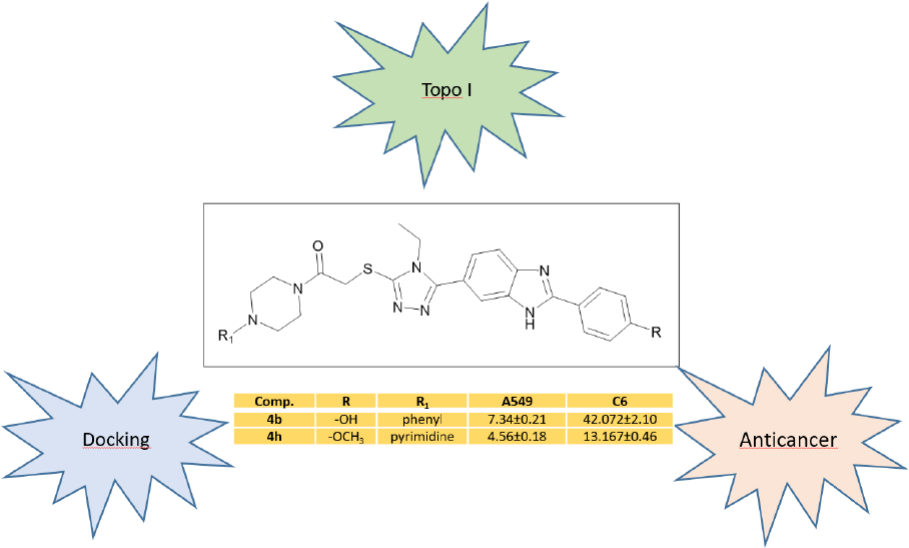

In this study, we designed, synthesized, and evaluated a series of 1,2,4-triazole benzimidazoles for their cytotoxic effects against the A549, C6, and NIH3T3 cell lines. Additionally, these compounds were assessed for their inhibitory activity against DNA topoisomerase I, aiming to develop novel anticancer agents. The synthesized final compounds **4a**–**h** were characterized using ^1^H NMR, ^13^C NMR, and HRMS. Among them, compounds **4b** and **4h** emerged as the most potent agents against the A549 cell line, exhibiting an IC_50_ value of 7.34 ± 0.21 μM and 4.56 ± 0.18 μM, respectively. These results were compared to standard drugs, doxorubicin (IC_50_ = 12.420 ± 0.5 μM) and Hoechst 33342 (IC_50_ = 0.422 ± 0.02 μM). Notably, all tested compounds displayed higher cytotoxicity toward A549 cells than C6 cells. Compounds **4b** and **4h** demonstrated significant inhibitory activity against topoisomerase I, highlighting their potential as lead compounds in anticancer therapy. Subsequent in silico molecular docking studies were conducted to elucidate the potential binding interactions of compounds **4b** and **4h** with the target enzyme topoisomerase I. Molecular dynamics studies also assessed and validated the binding affinity and stability. These studies confirmed the promising binding affinity of these compounds, reinforcing their status as lead candidates. According to DFT, compound **4b** having the lower energy gap value (Δ*E* = 3.598 eV) is more chemically reactive than the others, which is consistent with significant inhibitory activity against topoisomerase I. Furthermore, in silico ADME profiles for compounds **4b** and **4h** were evaluated using SwissADME, providing insights into their pharmacokinetic properties.

## Introduction

Cancer is caused by the abnormal and uncontrolled proliferation of cells and is presently the second leading cause of death worldwide.^1^ Although numerous approved drugs with various mechanisms have been used in cancer therapy, their advantages are frequently outweighed by their poor safety and efficacy records. Therefore, it is still difficult to find and produce anticancer drugs with strong therapeutic efficacy and few side effects.^2,3^ Despite the recent advances in targeted chemotherapy, discovery of novel anticancer agents that selectively inhibit the growth of cancer cells is one of the most beneficial candidates for considered strategies.^4,5^

Topoisomerases are a group of enzymes that regulate DNA supercoiling and entanglements during important cellular processes such as replication, transcription, recombination, and repair.^6,7^ Human topoisomerase can be classified into two primary categories based on whether it cleaves single or double strands of DNA: type I (Topo I) and type II (Topo II).^8−11^

Topo I inhibitors are divided into Topo I poisons and catalytic inhibitors based on their mode of action.^12,13^ The Topo I poison stabilizes the DNA–Topo I complex to form a transient and cleavable DNA–Topo I covalent complex and prevent the cleaved DNA strand from relegation, thus leading to the accumulation of undesired truncated DNA. In contrast with the Topo I poison, the Topo I catalytic inhibitor inhibits Topo I-mediated DNA cleavage in more diverse manners. They may intercalate between the DNA bases to inhibit the association of DNA with Topo I to bind with Topo I and prevent the interaction with DNA or interact with Topo I and allow the assembly of Topo I and DNA but inhibit the formation of the Topo I cleavage complex.^14^ Topo I is a key target for anticancer drug discovery because of the significant role it plays in cellular processes.^15^ Because of the crucial role in the maintenance and replication of DNA during cancer cell proliferation, Topo inhibitors are therefore a major class of anticancer agents for cancer treatment.^16^

The benzimidazole nucleus emerged as a key pharmacophore in cancer research because of its broad anticancer potential and adaptable tumor inhibitory mechanisms, in addition to its simple synthesis methods for obtaining a variety of derivatives. The benzimidazole motif is present in several known anticancer medications as well as various bioactive compounds.^17^ Through several modes of action, the benzimidazole scaffold is essential to the creation of anticancer medicines such as bendamustine, carbendazim, nocodazole, and veliparp.^18^ Hoescht 33258 and Hoechst 33342 are examples of benzimidazoles, a structurally distinct class of Topo I poisons that function as DNA minor groove binders.^19−22^

When the literature is examined, In 2023, Othman et al., designed and synthesized a series of benzimidazole-triazole derivatives. Among the tested compounds, compound **5a** (IC_50_= 3.87–8.34 μM) was the most potent antitumor agents against HepG-2, HCT-116, MCF-7,and HeLa cancer cells lines, with activity comparable to that of Dox (IC_50_ = 4.17–5.57 μM). The compound **5a** exerted strong inhibitory activity on Topo II (IC_50_ = 2.52 μM) which is better than Dox (IC_50_ = 3.62 μM).^23^ In 2022, Nawareg et al., synthesized three different series (hydrazone, oxadiazole, and triazole) containing the benzimidazole structure. They were further investigated for Topo II enzyme inhibition, where hybrids 13 (hydrazine drivetive) and 20 (oxadiazole derivative) were the most active candidates with IC_50_ values of 6.72 and 8.18 μM respectively compared to staurosporine (IC_50_ = 4.64 μM). They were further investigated for Topo II enzyme inhibition, where hybrids 13 (hydrazine drivetive) and 20 (oxadiazole derivative) were the most active candidates with IC_50_ values of 6.72 and 8.18 μM respectively compared to staurosporine (IC_50_ = 4.64 μM). Compounds 13 and 20 showed a good binding with Topo II catalytically active sites via the key amino acids, that confirmed their higher Topo II inhibitory activity.^24^

**Figure 1:**
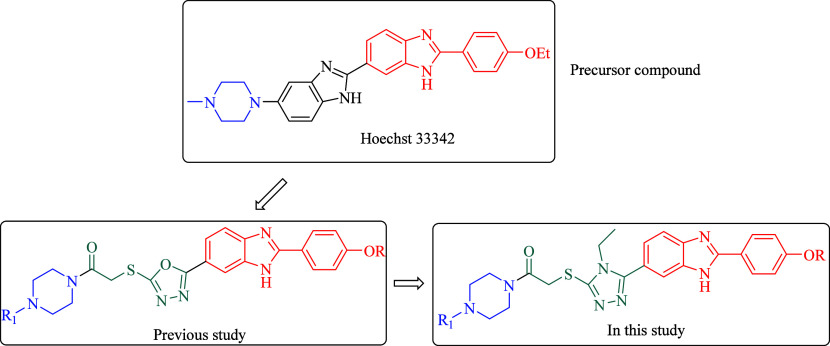
Design strategies of some new benzimidazole derivatives as anticancer agents.

In previous study, Hoechst compounds have been identified as precursor compounds.^25^ Instead of bisbenzimidazole, the 1,3,4-oxadiazole ring attached to the benzimidazole ring was synthesized. Promising results were obtained as a result of the study. In this study, the triazole ring was used instead of the oxadiazole ring as illustrated in Figure 1. The cytotoxicity of these synthesized compounds to different cell lines, and Topo I inhibitory activities were evaluated. To determine the possible interactions of compounds, that showed high activity, docking studies have been performed. In this study, 12 newly synthesized compounds were optimized with the Density Functional Theory (DFT) method and their electronic properties were calculated in order to understand which of the substituent groups added to both sides of the benzimidazole-triazole main skeleton is more stable and chemically more active.

## Results and Discussion

### Chemistry

**Figure 2:**
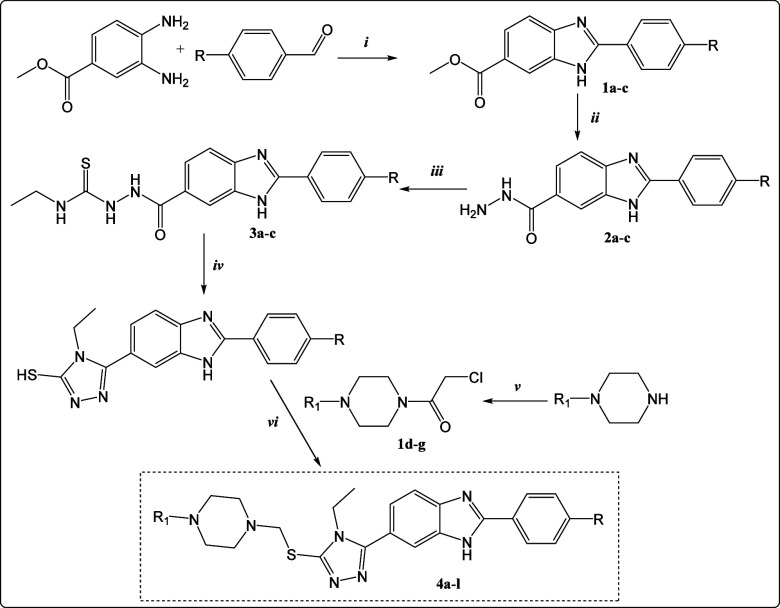
Synthesis procedure for obtaining the target compounds (**4a**–**l**). Reagent and conditions: (i) Na_2_S_2_O_5_, DMF, 240 °C, 10 bar, 5 min by MWI; (ii) NH_2_NH_2_·H_2_O, 240 °C, 10 bar, 10 min by MWI; (iii) ethylthiocyanate, EtOH, reflux; (iv) NaOH/EtOH, reflux 2h; HCl to pH = 2, ice water precip; (v) piperazine/THF, TEA, chloracetyl chloride/THF, 0 °C, stir; (vi) piperazines/acetone, 40 °C, 12h, reflux.

The synthesis pathway of the designed and synthesized benzimidazole derivatives (**4a**–**l**) is showed in Figure 2. In the first step of the synthesis studies, 4-substituted benzaldehyde and sodium disulfide were reacted in dimethylformamide under microwave irradiation, and as a result of the condensation reaction of the resulting benzaldehyde sodium bisulfite adduct and 3,4-diamino benzoate under microwave irradiation, methyl 2-(4-substitutedphenyl)-1H-benzimidazole-6-carboxylate (**1a**–**c**) derivatives were obtained. In the next step, compounds **1a**–**c** were treated with hydrazine hydrate under microwave irradiation to obtain 2-(4-substitutedphenyl)-1H-benzo[d]imidazole-6-carbohydrazide (**2a**–**c**) derivatives. The synthesized hydrazide derivatives (**2a**–**c**) were refluxed with ethyl thiocyanate in ethanol and the precipitated product was collected. After the precipitated product dries, the compounds (**2a**–**c**) were refluxed with NaOH in ethanol to obtain triazole derivatives (**3a**–**c**). As a result of the acetylation reaction between various 1-substituted piperazine derivatives and chloroacetyl chloride, 2-chloro-1-(4-substitutedpiperazine-1-yl)-ethan-1-one derivatives (**1d**–**g**) were obtained. The last reaction step was carried out between compounds **3a**–**c** and **1d**–**h** and 2-((5-(2-(4-substitutedphenyl)-1H-benzo[d]imidazol-6-yl)-4H–1,2,4-triazol-3-yl)thio)-1-(4-substitutedpiperazin-1-yl)-ethan-1-one target compounds (**4a**–**l**) were obtained.

The structures of final compounds **4a**–**l** were characterized by NMR and HRMS. In the ^1^H NMR spectra, singlet protons between 4.32 and 5.44 ppm were assigned to −CH_2_ attached to the carbonyl group. Piperazine protons resonated between 2.30 and 3.89 ppm. The peaks in the range of 3.80–4.47 ppm for compounds **4e**–**4h** were attributed to the presence of the methoxy group. Aromatic protons were observed at between 6.66 and 8.52 ppm. In the ^13^C NMR spectra, the peaks due to aliphatic carbons were determined at 12.33–64.40 ppm whereas aromatic carbons and carbonyl carbons resonated between 103.11 and 169.10 ppm. In the spectra of HRMS, the determined molecular weights were consistent with the expected values.

### Cell Viability Assay

The antiproliferative activity of synthesized compounds **4a**–**l** against A549 (lung carcinoma cell line) and C6 (rat glioma cell line) as well as NIH3T3 (mouse embryo fibroblast cell line) was represented in Table 1. The majority of the synthesized compounds exhibit low to moderate activity against the tested cancer cell lines in comparison to doxorubicin and Hoechst 33342. Compounds **4b** and **4h** were more potent than doxorubicin (IC_50_ = 12.420 ± 0.5 μM) against A549 cell line with the IC_50_ value of 7.34 ± 0.21 and 4.56 ± 0.18 μM, respectively. Compound 4h was the most cytotoxic against C6 cell line with the IC_50_ value of 13.167 ± 0.46 μM, which is higher than that of doxorubicin and lower than that of Hoechst 33342. The antiproliferative activity of compounds **4c** (IC_50_= 15.84 ± 0.63) and 4l ((IC_50_= 12.43 ± 0.54) was comparable with doxorubicin against A549 cells.

**Table 1:**
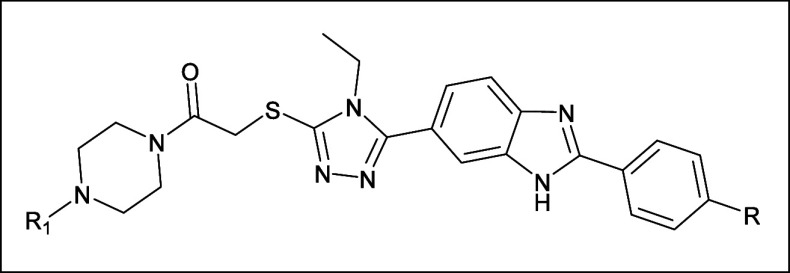
IC_50_ Values (μM) of the Compounds in A549, C6 Cell Lines

comp.	R	**R**_**1**_	A549	C6	NIH3T3
**4a**	–OH	ethyl	21.80 ± 1.04	>100	64.85 ± 1.96
**4b**	–OH	phenyl	7.34 ± 0.21	42.072 ± 2.10	56.69 ± 1.18
**4c**	–OH	pyridine	15.84 ± 0.63	24.026 ± 2.40	32.18 ± 0.74
**4d**	–OH	pyrimidine	54.42 ± 1.84	>100	80.50 ± 2.76
**4e**	–OCH_3_	ethyl	75.67 ± 2.55	>100	77.68 ± 2.47
**4f**	–OCH_3_	phenyl	69.43 ± 3.04	>100	92.84 ± 3.06
**4g**	–OCH_3_	pyridine	87.21 ± 3.97	>100	85.94 ± 2.92
**4h**	–OCH_3_	pyrimidine	4.56 ± 0.18	13.167 ± 0.46	74.44 ± 2.68
**4i**	–OC_2_H_5_	ethyl	32.98 ± 1.42	39.369 ± 2.58	30.64 ± 1.08
**4j**	–OC_2_H_5_	phenyl	54.12 ± 2.18	>100	69.54 ± 2.85
**4k**	–OC_2_H_5_	pyridine	24.86 ± 1.21	64.630 ± 2.66	46.36 ± 1.205
**4l**	–OC_2_H_5_	pyrimidine	12.43 ± 0.54	38.80 ± 1.82	35.69 ± 1.114
doxorubicin			12.420 ± 0.5	28.690 ± 1.22	1055.24 ± 9.125
Hoechst 33342			0.422 ± 0.02	1.051 ± 0.30	12.95 ± 0.598

It is crucial that an anticancer agent affects the cancer cell line but having minimal or no side-effect on healthy cells. For this purpose, the cytotoxic effects of the active compounds on the NIH3T3 cell line were investigated. It is seen that compounds **4b** and **4h**, which are especially effective on cancer cells, have high IC_50_ values on healthy cells. This shows that the compounds are not toxic to healthy cells at the IC_50_ values that are effective on cancer cells.

All compounds were found to be more potent against A549 cells than C6 cells. Compounds in the series can be divided into three groups according to the substituent (−OH, −OCH_3_, or −OC_2_H_5_) on phenyl ring to discuss the structure–activity relationships. Taking into consideration of the cytotoxic activity of the compounds against A549 cell line, the phenyl substituent attached to the piperazine ring enhanced the cytotoxic activity the most compared to ethyl group or pyridine and pyrimidine rings, in the structure of hydroxyl bearing **4a**–**4d** compounds. Considering the structures of methoxy containing compounds **4e**–**4h**, the pyrimidine substituent attached to piperazine ring is favorable than the other substituents, resulting the highest cytotoxic activity in the series. In the ethoxy bearing compounds **4i**–**4l**, the pyrimidine substituent attached to piperazine is contributed the most to the antiproliferative activity. The most active compounds **4b** and **4h** contain hydroxyl and methoxy groups on the phenyl ring; phenyl and pyrimidine rings attached to piperazine, respectively. When analyzed the cytotoxicity against the C6 cell line, the pyridine substituent is enhanced the activity in compounds **4a**–**4d**. In compounds **4i**–**4l**, all substituents except pyrimidine significantly reduced the activity. The contribution of the pyrimidine substituent in compound **4l** was the most between compounds **4i**–**4l**.

### DNA Topoisomerase I Assay

The anticancer activities of synthesized compounds (**4a**–**l**) were determined by the MTT method, IC_50_ values were calculated, and the DNA topoisomerase I inhibitory effects of the compounds **4b** and **4h**, which stand out in terms of activity, were evaluated. Topogen’s Topoisomerase I Drug Screening Kit was used to investigate the topoisomerase I effects of the compounds. With this kit, it is determined whether the compounds that inhibit Topo I activity act as catalytic inhibitors or topoisomerase poisons. Topoisomerase I inhibition activities of compounds **4b**, **4h**, Hoechst 33342, and camptothecin were evaluated in vitro and the analysis of the reaction products was visualized by the electrophoresis method. Supercoiled plasmid DNA (pHOT 1) was used as the control group. As another control group, plasmid DNA and topoisomerase I were used to ensure that the DNA became relaxed. Hoechst 33342, known as a topoisomerase poison, and camptothecin were used as positive controls.

**Figure 3:**
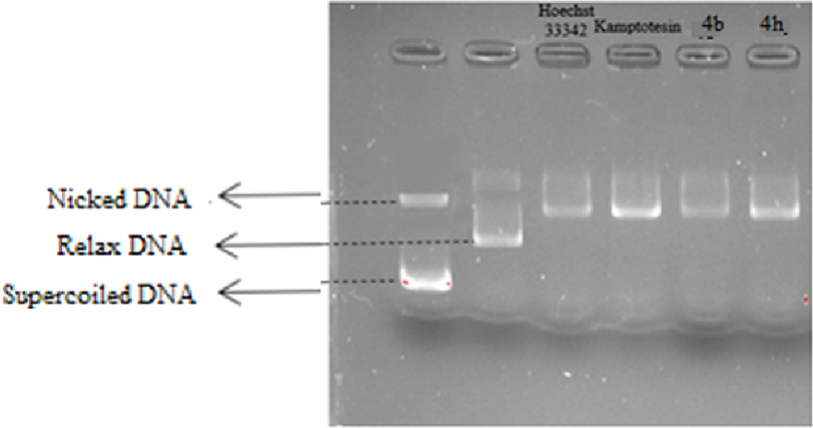
Topo I activity of compounds **4b**, **4h**, Hoechst 33342, and camptothecin.

When gel images of Hoechst 33342 and camptothecin are examined, it is observed that the broken (nick) DNA band is thicker compared to the control and there is no band of supercoiled DNA in the medium. It was found that the controls used prevented the recombination of single-stranded DNA breaks created by the Topo I enzyme, causing an increase in single-stranded DNA breaks (nicks) and had a strong inhibitory effect as a topoisomerase poison. In Figure 3, it can be seen that the reference drugs and the synthesis compounds have very similar appearances, but no structure belonging to the superhelical DNA structure is seen. This shows that our compounds have a strong inhibitory effect as topoisomerase poisons. If the compounds had inhibited the catalytic activity of the enzyme, the DNA would not be able to relax and a supercoiled band would be observed in the gel image. If the compounds had no activity on Topo I, the Topo I enzyme would relax the DNA and a relaxed band would be observed in the gel image.

### Docking and Molecular Interactions

**Figure 4:**
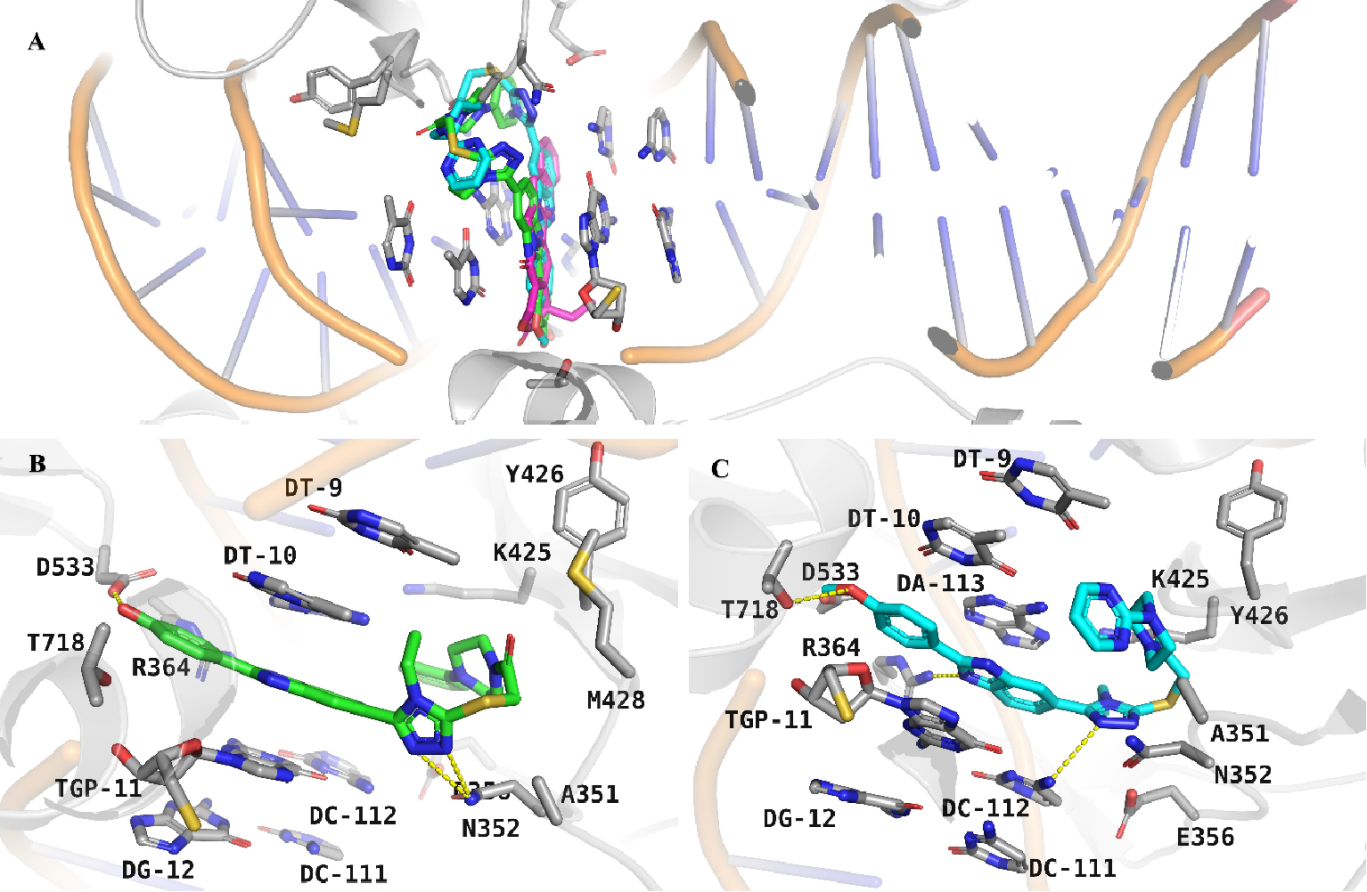
Molecular docking study of compounds **4b** and **4h** with human DNA topoisomerase I (PDB ID: 1T8I). (A) Whole pose visualization of **4b** (green), **4h** (cyan), and reference and cocrystal compound camptothecin (magenta). (B,C) Binding poses and H bond interaction details of **4b** and **4h** with human DNA topoisomerase.

In the molecular docking study, we showed the interaction profiles of compounds **4b** and **4h** with human DNA topoisomerase I (PDB ID: 1T8I) and their moderate potential as novel agents in cancer therapy.^25^ The binding poses of camptothecin and compounds **4b** and **4h** within the active site of human DNA topoisomerase I are comparatively visualized in Figure 4A. This depiction is instrumental in illustrating the conformational alignments and spatial orientation of the molecules within the active pocket. It is particularly noteworthy how compounds **4b** and **4h** are positioned relative to the reference compound, camptothecin, providing a foundational context for the analysis of subsequent interactions. The docking simulations were validated, as evidenced by the low RMSD of 0.45 Å achieved during the redocking of camptothecin, emphasizing the predictive model’s precision.^27^

In vitro experimental data corroborated the binding patterns of compound **4b** at the enzyme’s active site. It formed key hydrogen bonds with N352, D533, and R364, which enhanced specificity and anchored the compound within the active site. A C–H bond with N352 was noted for further stabilization. Pi-anion interactions with E356 and the nucleotide backbones of DT-9 and DA-113 were significant, suggesting an interaction with the DNA, potentially perturbing its normal function. Hydrophobic interactions through van der Waals forces with amino acid residues K425, I427, Y426, W416, and P431, as well as pi-alkyl interactions with DT-10, L429, M428, and A351, contributed to a favorable binding milieu (Figure 4B).

Correspondingly, the in vitro efficacy of compound **4h** aligned with its predicted interaction map, comprising hydrogen bonds with N352, R364, and D533. Pi-pi stacking with R364 and DA-113, alongside pi-alkyl interactions with DT-10 and other pivotal residues, established a robust binding profile, further solidified by van der Waals contacts, augmenting both the compound’s affinity and specificity (Figure 4C).

These interactions point to an intricate mechanism by which compounds **4b** and **4h** bind with the enzyme’s active site, also engaging the DNA substrate, indicative of a dual inhibitory action that significantly hampers the enzyme’s role in DNA replication. The docking results, now substantiated by in vitro findings, pave the way for studies to validate the inhibitory efficacy of compounds **4b** and **4h** further and to clarify their modes of action.

### Molecular Dynamics Studies

Based on the observed biological activity and molecular docking results, it is evident that compounds **4b** and **4h** have potential to serve as prime candidates as topoisomerase I inhibitors. To validate the binding poses of these protein–ligand complexes, we conducted a 300 ns molecular dynamics simulation. This investigation aimed to unveil the dynamic alterations occurring in the presence of these ligands during the simulation. Molecular dynamics studies are commonly employed to scrutinize the properties of macromolecules and analyze the physical movements of atoms and molecules. The ensuing results from the simulation are detailed below.

### Examining Protein–Ligand Interactions via a 300 ns Simulation: Investigating the Behavior of the Human DNA Topoisomerase I Protein (PDB-1T8I) when Interacting with Molecules **4b** and **4h**

#### Analysis of RMSD

**Figure 5:**
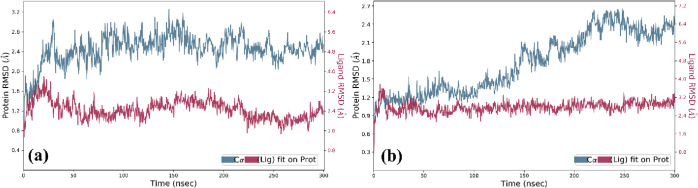
RMSD plot depicting the 300 ns simulation trajectory for the (a) 1T8I–**4b** complex and (b) 1T8I–**4h** complex.

The RMSD value represents the average displacement change of a specific group of atoms within a frame compared to a reference frame. In this study, the LigfitProt was employed to assess both ligand and protein RMSD, aligning the protein–ligand complex with the reference protein backbone. In the 1T8I–**4b** complex (Figure 5a), the ligand RMSD remained relatively steady, fluctuating around 2.4 Å throughout the simulation. The protein exhibited a consistent RMSD of 2.4 Å with only minor fluctuations. In contrast, for the 1T8I–**4h** complex (Figure 5b), the ligand displayed exceptional stability throughout the trajectory, maintaining a constant RMSD of 3.2 Å without significant fluctuations. The protein, when in the ligand-bound state, exhibited minimal variations. It initially had an RMSD of 0.9–1.6 Å from 0 to 150 ns and later displayed an RMSD of 1.8–2.4 Å from 150 to 300 ns.

#### Analysis of RMSF

**Figure 6:**
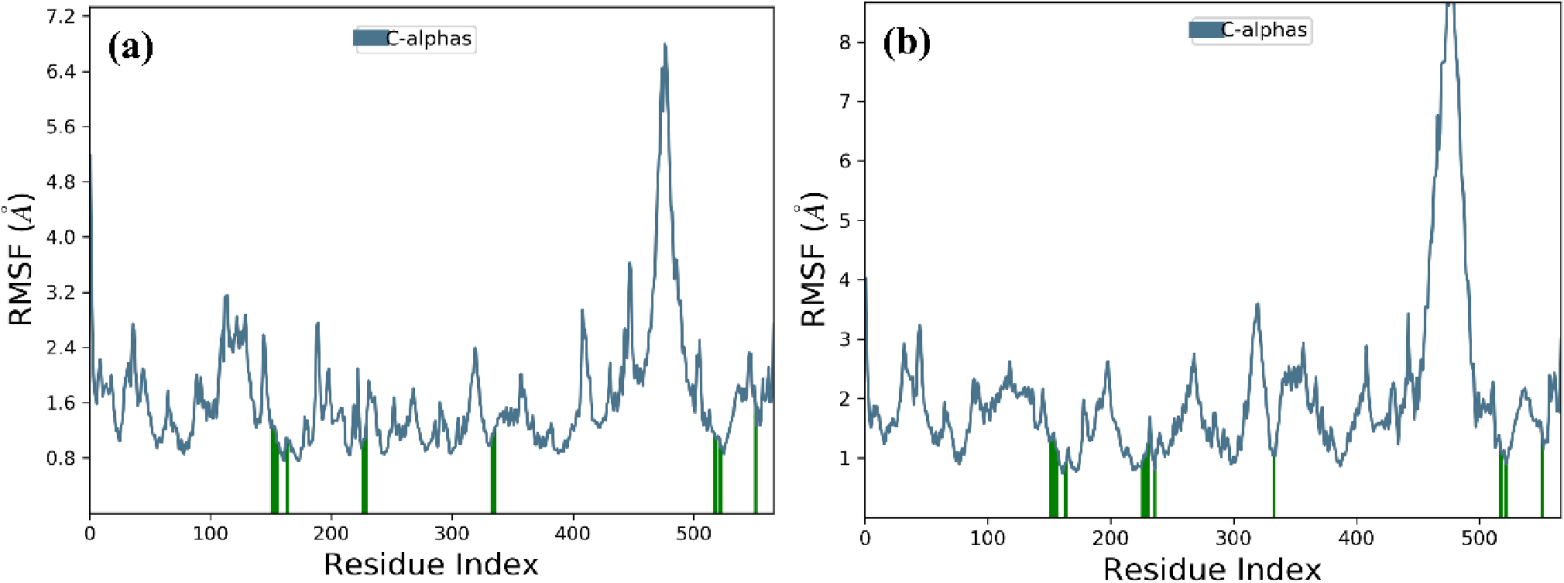
RMSF plot depicting the 300 ns simulation trajectory for the (a) 1T8I–**4b** complex and (b) 1T8I–**4h** complex.

To illustrate local variations along the protein chain, Root Mean Square Fluctuation (RMSF) provides valuable insights. Peaks on the RMSF chart pinpoint the regions of the protein undergoing the most significant fluctuations during the simulation. Typically, the N- and C-terminal tails of the protein exhibit greater mobility compared to other areas. Structured elements such as alpha helices and beta strands, being more rigid, undergo fewer changes than flexible loop sections. In the presence of **4b** (Figure 6a), the protein’s RMSF profile remains relatively stable at 2.4 Å, except for amino acids ranging from 450 to 500, where higher fluctuations, reaching 6.4 Å, are observed, primarily within the loop regions. For 4h (Figure 6b), a similar trend is observed, with fluctuations occurring in amino acids from 450 to 500, reaching ranges of over 8 Å, but eventually stabilizing.

#### Analysis of the Percentage of Protein–Ligand Contacts

**Figure 7:**
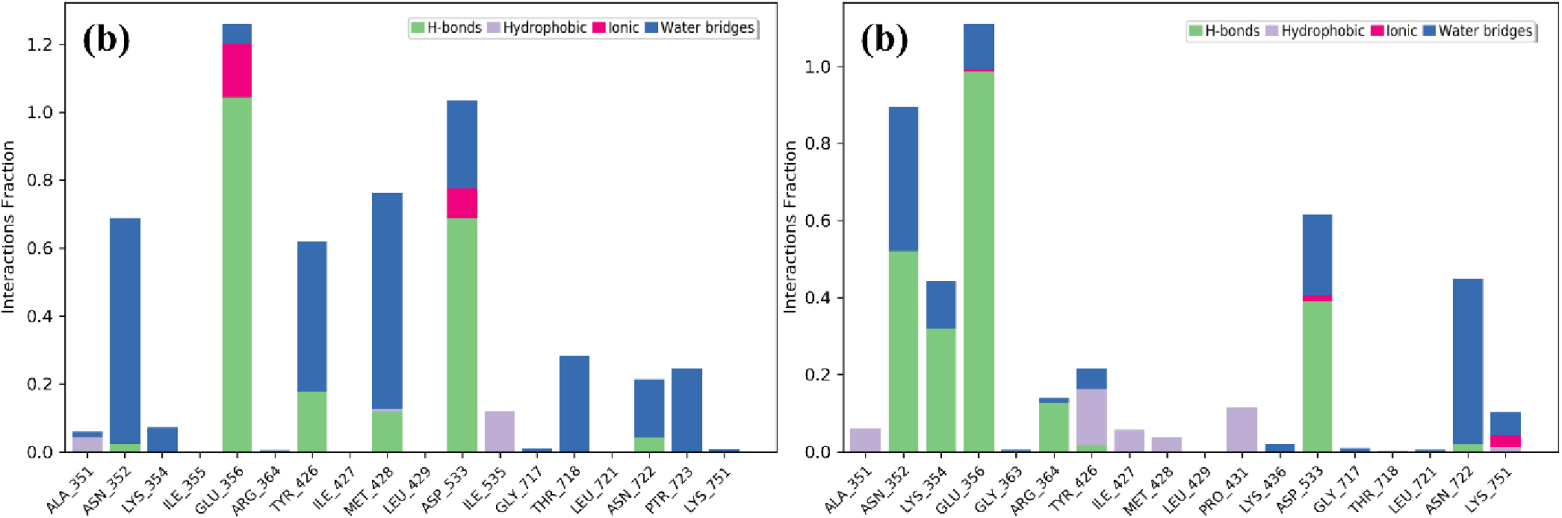
Stacked bar charts illustrating protein interactions with (a) 1T8I–**4b** complex and (b) 1T8I–**4h** complex throughout the simulation, showcasing the percentage of protein–ligand contacts.

**Figure 8:**
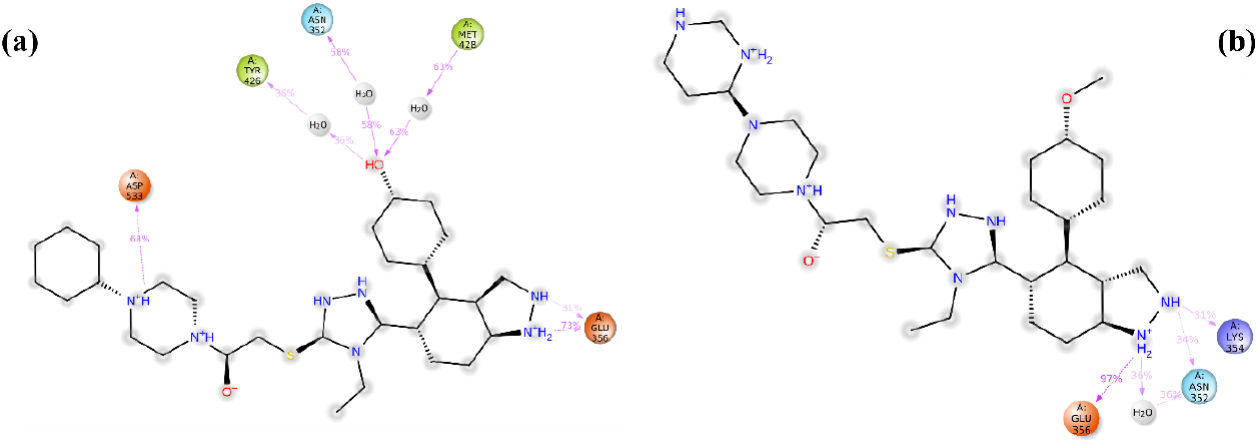
In-depth atomic interactions of (a) **4b** and (b) **4h** with the critical amino acid residues at the active site of 1T8I.

Throughout the simulation, we meticulously monitored the protein–ligand contacts, with a particular emphasis on hydrogen bonds, which play a pivotal role in ligand binding. Understanding the properties of these hydrogen bonds is crucial in drug development due to their significant impact on drug specificity, metabolism, and absorption. In the case of the 1T8I–**4b** complex (Figure 7a), we observed hydrogen-bonding interactions with Glu356 (100%), Tyr426 (20%), Met428 (20%), and Asp533 (70%). Some of these hydrogen bonds were consistent with those identified in docking studies. Furthermore, the complex engaged in hydrophobic interactions and water bridge interactions with Ile535, Asn352, Thr718, Asn722, and Lys354. Ionic bonds were also observed with Glu256 and Asp533. Similarly, in the 1T8I–**4h** complex (Figure 7b), the ligand positioned itself within the active site and formed hydrogen bonds with Asn352 (50%), Lys354 (35%), Glu356 (100%), Arg364 (15%), and Asp533 (40%). The active site pocket facilitated various interactions, including ionic bonds (with Asp533 and Lys751), hydrophobic contacts (involving Ala351, Tyr426, Ile427, Met428, and Pro431), and water bridges with Asn352, Lys354, Lys436, Asn722, and Lys751. Additionally, a detailed analysis of atomic interactions for **4a** and **4h** is presented in Figure 8a,b, respectively.

#### Timeline Visualization of Protein–Ligand Contacts

**Figure 9:**
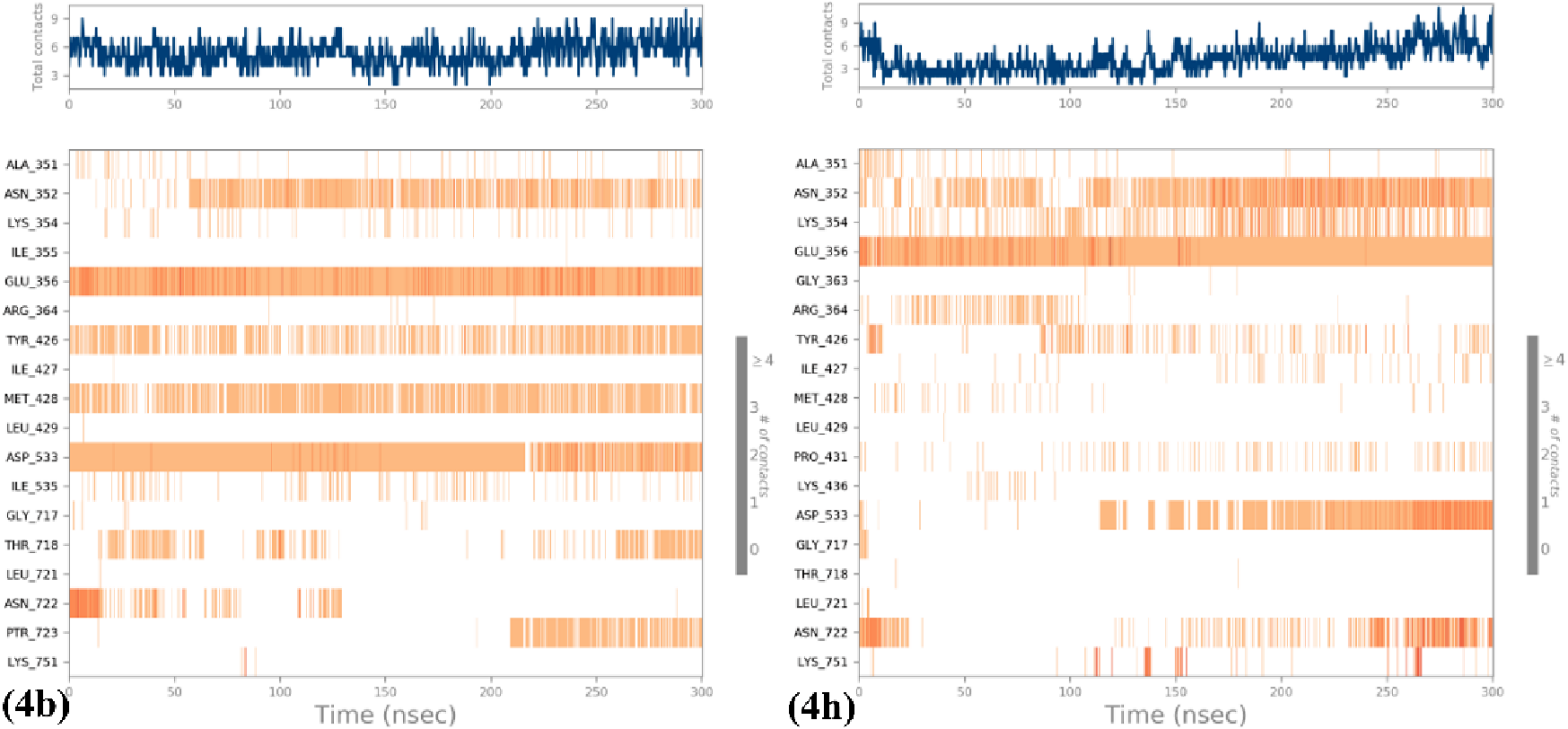
Timeline visualization of protein–ligand contacts of the (a) 1T8I–**4b** complex and (b) 1T8I–**4h** complex.

The various interactions and contacts, such as hydrogen bonds, hydrophobic interactions, ionic bonds, and water bridges, are visually represented in Figure 9 as a timeline. In the top panel (dark blue), one can observe the cumulative count of distinct interactions formed by the protein with the ligand over the entire trajectory. In the bottom panel, a visualization of the specific residues that interact with the ligand in each frame of the trajectory has been shown. The color scale on the right side of the plot indicates that certain residues establish multiple specific contacts with the ligand, represented by a deeper shade of orange.

Compounds **4b** and **4h** underwent an MM-GBSA analysis to elucidate the binding energies with the respective human DNA Topoisomerase I Protein (PDB-1T8I). This analytical approach serves to ascertain the stability of the protein–ligand complex postbinding to the active site. The MM-GBSA analysis revealed that both **4b** and **4h** demonstrated optimal binding energies with the human DNA Topoisomerase I Protein: −86.64 and −90.88 kcal/mol. This substantiates their potential for binding and inhibiting the proteins in question.

### Quantum Mechanical Calculations

The optimized geometries of all structures correspond to true minima, as no imaginary frequencies were observed in the vibration frequency survey. In order to investigate the electronic properties of current molecules, molecular electrostatic potential (MEP) and HOMO–LUMO analyzes were performed at the B3LYP/6-31G(d,p) level. HOMO–LUMO energies (eV) and calculated global reactivity parameters of the compounds are given in Table 2.

**Table 2:** HOMO–LUMO Energies (eV) and Calculated Global Reactivity Parameters of the Best Stable States of the Compounds **4a**–**4l** at the B3LYP/6-31G(d,p) Level in the Gas Phase^a^

compound	*E*_L_ (eV)	*E*_H_ (eV)	Δ*E* (eV)	IP (eV)	EA (eV)	χ (eV)	η (eV)	σ (eV)^−1^	μ (eV)	ω (eV)
**4a**	–1.190	–5.353	4.163	5.353	1.190	3.272	2.082	0.240	–3.272	2.572
**4b**	–1.378	–4.976	3.598	4.976	1.378	3.177	1.799	0.278	–3.177	2.806
**4c**	–1.373	–5.237	3.864	5.237	1.373	3.305	1.932	0.259	–3.305	2.826
**4d**	–1.218	–5.393	4.175	5.393	1.218	3.306	2.088	0.239	–3.306	2.617
**4e**	–1.161	–5.317	4.156	5.317	1.161	3.239	2.078	0.241	–3.239	2.524
**4f**	–1.345	–4.971	3.626	4.971	1.345	3.158	1.813	0.276	–3.158	2.751
**4g**	–1.342	–5.224	3.882	5.224	1.342	3.283	1.941	0.258	–3.283	2.776
**4h**	–1.187	–5.356	4.169	5.356	1.187	3.271	2.085	0.240	–3.271	2.567
**4i**	–1.138	–5.294	4.156	5.294	1.138	3.216	2.078	0.241	–3.216	2.489
**4j**	–1.320	–4.970	3.650	4.970	1.320	3.145	1.825	0.274	–3.145	2.710
**4k**	–1.320	–5.221	3.900	5.221	1.320	3.270	1.950	0.256	–3.270	2.742
**4l**	–1.167	–5.329	4.162	5.329	1.167	3.248	2.081	0.240	–3.248	2.534

a Gap Δ*E*: (*E*_LUMO_–*E*_HOMO_), IP (−HOMO): ionization potential, EA (−LUMO): electron affinity, χ(IP+EA)/2: electronegativity, η (IP–EA)/2: chemical hardness, σ (1/2η): chemical softness, μ −(IP+EA)/2: chemical potential, ω (μ2/2η): electrophilic index.

**Figure 10:**
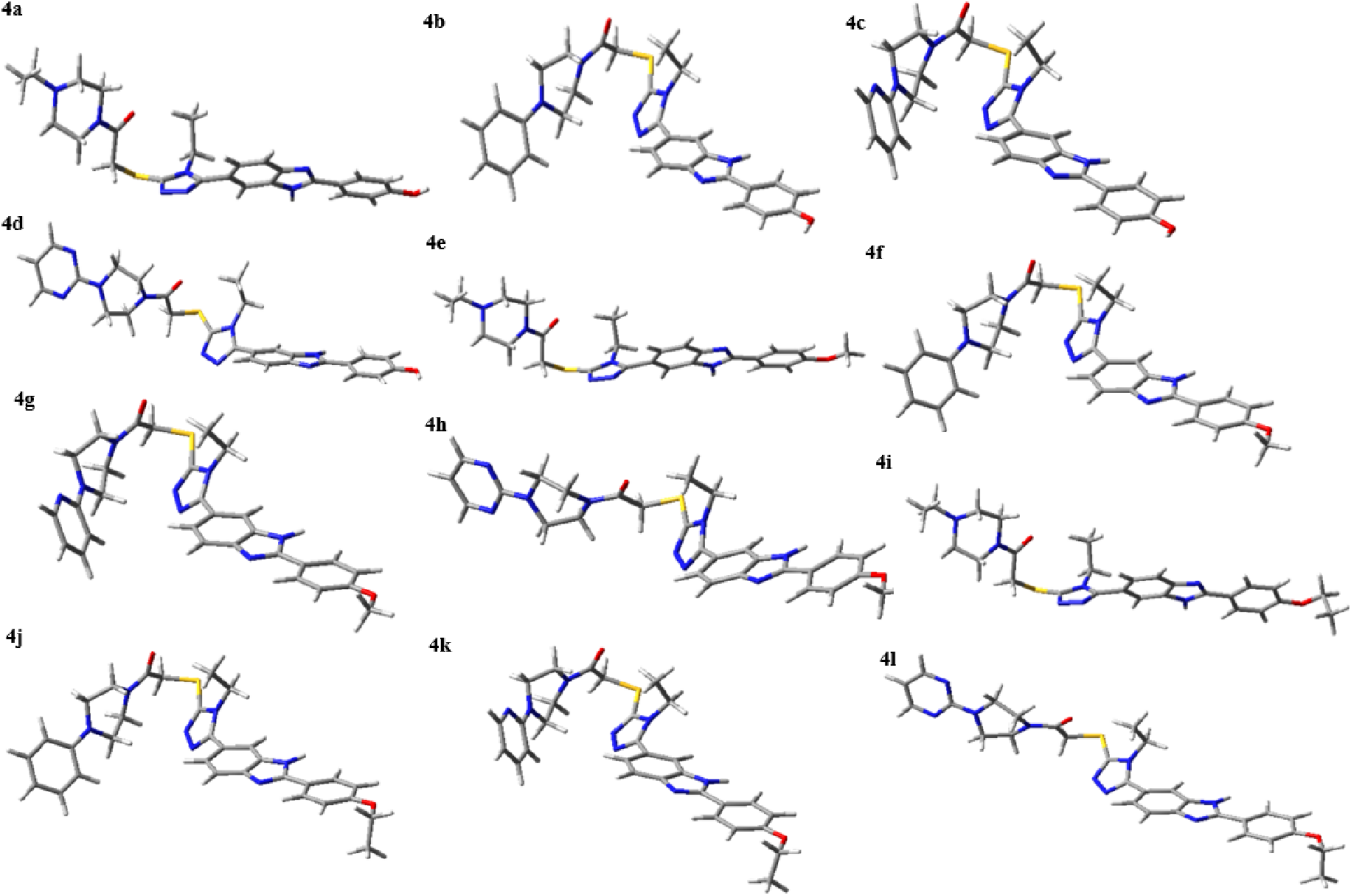
Optimized geometries of **4a**–**l** molecules at the B3LYP/6-31G(d,p) level.

The main molecule in building blocks of the compounds consists of the 1,2,4-triazol ring with a thio group attached to the R1-piperazin ethanone group and R-phenyl-benzimidazole group at third and fifth position of this ring, respectively (Figure 10).

According to HOMO–LUMO analysis, the chemical reactivity order is **4b**> **4f** > **4j**> **4c** > **4g**> **4k** > **4e** = **4i**> **4l** > **4a**> **4h** > **4d**. Compound **4b** (with lower Δ*E* = 3.598 eV) is more chemically reactive than the other molecules; this is consistent with significant inhibitory activity against Topoisomerase I and highlights their potential as lead compounds in anticancer therapy. The electrophilic indexes (ω) of all molecules belong to a strong electrophilc group as their values are bigger than 1.50 eV.^28^

The main molecule in building blocks of the compound **4b** consists of the 1,2,4- triazol ring with a thio group attached to a phenyl-piperazin ethanone group and a hydroxyphenyl-benzimidazole group at third and fifth location of this ring, respectively (Figure 10). The rotation of the hydroxyphenyl-benzimidazole group with respect to the 1,2,4-triazole ring is measured as 134.7°, while the rotation of the thio group attached to the phenyl-piperazin ethanone group to this ring is −134.4°. These values indicate that the molecule is not planar, as shown in Figure 10. All molecules show the same trend.

**Figure 11:**
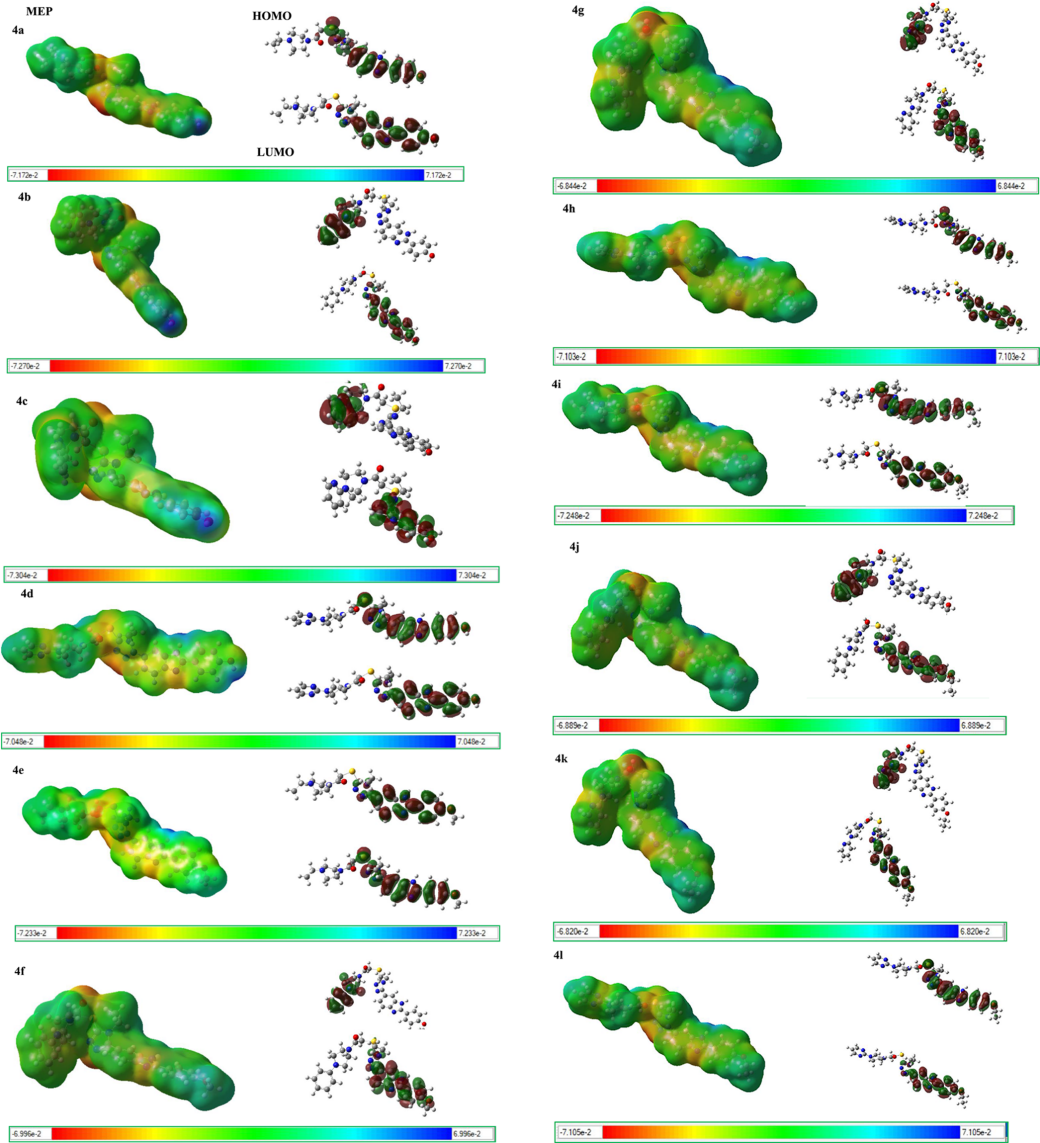
Molecular electrostatic potential (MEP) and HOMO–LUMO diagrams of the compounds **4a**–**l** at the B3LYP/6-31G(d,p) level. Atom colors: carbon in gray, nitrogen in blue, oxygen in red, sulfur in yellow, and hydrogen in white. The surfaces plotted by the 0.0004 electrons/b3 contour of the electronic density. (for **4a** molecule: color ranges, in au: blue, more positive than 0.0717; green, between 0.0717 and 0; yellow, between 0 and −0.0717; red, more negative than −0.0717).

In **4a**, **4d**, **4e**, **4h**, **4i**, and **4l** molecules, HOMOs are distributed in the 1,2,4- triazol ring linked a thio group and a phenyl-benzimidazole group, while LUMOs are localized in the phenyl-benzimidazole group. In other molecules, HOMOs and LUMOs are distributed in the R1-piperazine ring and the in phenyl-benzimidazole group, respectively (Figure 11).

According to MEP diagrams, negative regions with high electron density are observed around the N atoms in the triazole and benzimidazole rings and the O atom of ethanone, which are responsible for electrophilic attacks in all molecules. Positive regions having the low electron density of all molecules are formed around the N–H group of the benzimidazole ring, which are responsible for nucleophilic attacks. In addition, the nitrogen atoms in the triazole and benzimidazole rings and the oxygen atoms attached to the phenyl ring are atoms that can make possible hydrogen bonds, confirming also by molecular docking studies for **4b** and **4h**. These hydrogen bonds contribute to the stabilization of protein–ligand interaction.

### ADME Estimation

**Figure 12:**
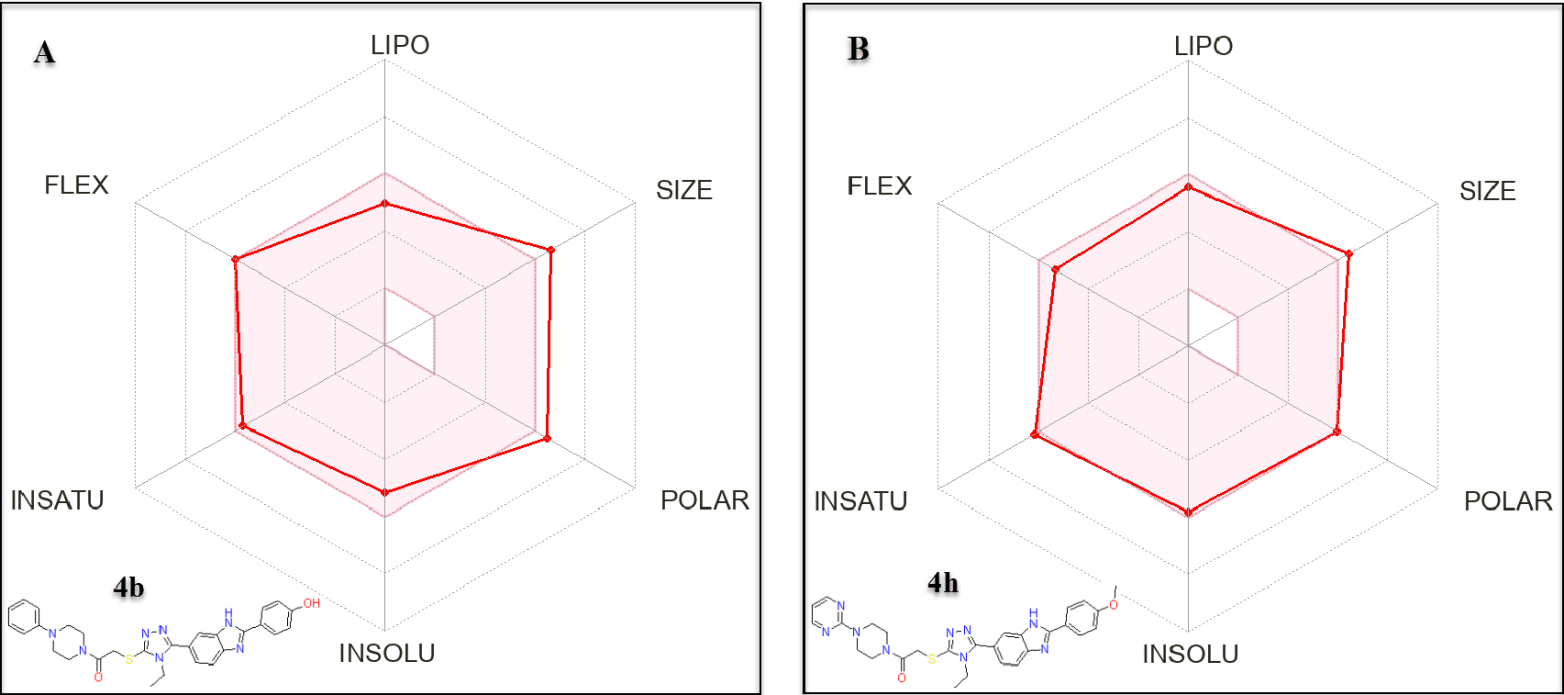
Radar plots illustrating the ADME profiles of (A) compound **4b** and (B) compound **4h**, highlighting their respective physicochemical attributes such as lipophilicity (LIPO), molecular size (SIZE), flexibility (FLEX), polarity (POLAR), solubility (INSOLU), and saturation (INSATU).

In the exploration of the ADME profiles of compounds **4b** and **4h**, this part provides an analysis of their pharmacokinetic and physicochemical properties, which are crucial determinants in their potential as therapeutic agents. Computational ADME studies were performed by entering the similes of the compounds on the SwissADME server.^29^ The radar plots (Figure 12A,B) graphically synthesize the predictive data, encapsulating the compounds’ profiles in terms of lipophilicity (LIPO), size, polarity, solubility (INSOLU), saturation (INSATU), and flexibility (FLEX). These plots indicate high lipophilicity for both compounds, which may favor membrane permeability but also suggest the possibility of bioaccumulation and nonspecific interactions. The substantial molecular size of both compounds could pose challenges in absorption and distribution, while their moderate flexibility, indicated by the number of rotatable bonds 9 for **4b** and 8 for **4h** may enhance binding to biological targets.

Compound **4b**, with a molecular weight of 555.65 g/mol and a Csp3 of 0.29, possesses a moderate level of lipophilicity, as reflected by a consensus Log P_o/w of 2.96. Despite its moderate size and polar surface area (TPSA of 143.25 Å^2^), it is predicted to have low GI absorption and is not BBB permeant, highlighting potential limitations in its central nervous system activity. It acts as a substrate for P-glycoprotein, which could affect bioavailability, and inhibits a range of cytochrome P450 enzymes, suggesting possible drug–drug interaction implications. The synthesis of **4b**, with a score of 3.97, is deemed moderately complex.

In comparison, **4h** has a slightly lower molecular weight of 539.65 g/mol, suggesting a potentially more rigid structure. With a fraction of Csp3 of 0.24, it has a higher lipophilicity, indicated by a consensus Log P_o/w of 3.63 and a lower TPSA of 128.47 Å^2^, which could impact its solubility and permeability. Like **4b**, **4h** is predicted to have low GI absorption and is not BBB permeant, also serving as a P-gp substrate and inhibiting several CYP450 enzymes. It presents a moderately favorable bioavailability score of 0.55, with a synthetic accessibility score of 3.90, suggesting comparable complexity to **4b** in its synthesis.

Both compounds, while exhibiting promising attributes such as significant lipophilicity and flexibility, show deviations from ideal chemical property spaces as defined by Lipinski’s rule and others, underscoring the need for further optimization. The radar plots, with their red-filled areas, provide a comparative understanding of the compounds’ profiles, illuminating the balance between drug-likeness attributes and challenges.

## Conclusion

In this study, we successfully hybridized benzimidazole with 1,2,4-triazole to synthesize a series of compounds, aiming to discover potent topoisomerase I inhibitors for cancer therapy. The synthesized compounds were in vitro assessed for their cytotoxic effects on A549 lung carcinoma and C6 rat glioma cell lines. Among them, compounds **4b** and **4h** demonstrated remarkable cytotoxicity against the A549 cell line, with IC_50_ values of 7.34 ± 0.21 μM and 4.56 ± 0.18 μM, respectively, surpassing the standard drug doxorubicin (IC_50_ = 12.420 ± 0.5 μM). This highlights their potential as more effective alternatives in targeting cancer cells. The significant inhibitory activity of these compounds against DNA topoisomerase I underscores their potential as lead compounds in anticancer drug development. Furthermore, the in silico molecular docking and dynamics studies revealed that compounds **4b** and **4h** have binding modes with the active site of topoisomerase I, confirming their mechanism of action and reinforcing their candidacy as potent anticancer agents. In addition, the in silico ADME profiling using SwissADME indicated favorable pharmacokinetic properties, enhancing their viability as drug candidates.

## Experimental Section

### Chemistry

All the chemicals employed in the synthetic procedure were purchased from Sigma-Aldrich Chemicals (Sigma-Aldrich Corp., St. Louis, MO, USA) or Merck Chemicals (Merck KGaA, Darmstadt, Germany). Melting points of the obtained compounds were determined by MP90 digital melting point apparatus (Mettler Toledo, OH, USA) and were uncorrected. ^1^H NMR and ^13^C NMR spectra of the synthesized compounds were obtained by a Bruker 300 and 75 MHz digital FT-NMR spectrometer (Bruker Bioscience, Billerica, MA, USA) in DMSO-d6, respectively. Splitting patterns were designated as follows: s: singlet; d: doublet; t: triplet; m: multiplet in the NMR spectra. Coupling constants (J) were reported as Hertz. All reactions were monitored by thin-layer chromatography (TLC) using Silica Gel 60 F254 TLC plates (Merck KGaA, Darmstadt, Germany).

#### General Procedure for the Synthesis of Methyl 2-(4-substitutedphenyl)-1H-benzimidazole-6-carboxylate Derivatives (1a–c)

4-Substituted benzaldehyde (0.03 mol), sodium disulfide (5.7 g, 0.03 mol), and DMF (10 mL) were placed in the microwave synthesis reactor vial (30 mL) and kept in the microwave synthesis reactor at 240 °C under 10 bar pressure for 5 min. At the end of this period, the mixture was removed from the reactor, and methyl 3,4-diaminobenzoate (0.03 mol) was added and subjected to microwave irradiation for another 5 min under the same reaction conditions. At the end of the reaction period, the product was poured into ice water and precipitated, filtered, washed with plenty of water, and crystallized from ethanol.

#### Synthesis of 2-(4-Substitutedphenyl)-1H-benzimidazole-6-carbohydrazide Derivatives (2a–c)

Methyl 2-(4-substitutedphenyl)-1H-benzimidazole-6-carboxylate (**1a**–**c**) (0.02 mol), ethanol (15 mL), and hydrazine hydrate (5 mL) was added into the microwave synthesis reactor vial (30 mL) and microwaved. It was kept in the synthesis reactor at 240 °C and under 10 bar pressure for 10 min. At the end of the reaction period, the product was poured into ice water and precipitated, filtered, then washed with plenty of water, and crystallized from ethanol.

#### Synthesis of 5-(2-(4-Substitutedphenyl)-1H-benzimidazol-6-Yl)-4-ethyl-1,2,4-triazole-3-thiol Derivatives (3a–c)

2-(4-Substitutedphenyl)-1H-benzimidazole-6-carbohydrazide (**2a**–**c**) and ethylthiocyanate were dissolved in ethanol and stirred under reflux for 2 h. The precipitated product was filtered and dried. The dried product was added to the sodium hydroxide solution in ethanol and stirred under reflux for 2 h. At the end of the reaction, HCl was added to the product until pH = 2, the product was precipitated by pouring it into ice water and was crystallized from ethanol by washing with plenty of water.

#### Synthesis of 2-Chloro-1-(4-substitutedpiperazin-1-Yl)-ethan-1-one Derivatives (1d–g)

4-Substituted piperazine derivatives (0.012 mol) were dissolved in tetrahydrofuran (THF) (50 mL). The solution was transferred to a dropping funnel by adding triethylamine (0.0132 mol, 1.90 mL). Chloracetyl chloride (0.014 mol, 1.056 mL) and THF (15 mL) were placed in a flask and placed in an ice bath prepared on a magnetic bottom heating stirrer. The mixture containing 4-substituted epiperazine was very carefully added dropwise to the reaction medium on the ice bath. During this time, care was taken to stir the reaction content vigorously. When the dripping process was completed, the reaction medium was taken from the ice bath and stirred at room temperature for 1 h. The resulting residue was filtered and washed with water.

#### Synthesis of the Target Compounds (4a–l)

5-(2-(4-Substitutedphenyl)-1H-benzimidazol-6-yl)-4-ethyl-1,2,4-triazole-3-thiol (**3a**–**c**) derivative compounds were dissolved in acetone and piperazine derivative compounds were added. The reaction mixture was kept at 40 °C under reflux for 12 h and acetone was evaporated at the end of the reaction. The remaining substance was filtered with water, dried, and crystallized from ethanol.

##### 2-((4-Ethyl-5-(2-(4-hydroxyphenyl)-1H-benzo[*d*]imidazol-6-yl)-4H-1,2,4-triazol-3-yl)thio)-1-(4-ethylpiperazin-1-yl)ethanone (4a)

Yield: 66%. Mp 223.5 °C. ^1^H NMR (300 MHz, DMSO-*d*_6_, ppm) δ: 1.00 (3H, t, *J* = 7.11 Hz, CH_3_), 1.25 (3H, t, *J* = 7.05 Hz, CH_3_), 2.30–2.38 (6H, m, piperazine CH), 3.48–3.49 (4H, m, piperazine CH, CH_2_), 4.06–4.08 (2H, m, CH_2_), 4.33 (2H, s, −CH_2_), 6.85 (2H, d, *J* = 8.58 Hz, 1,4- disubstitutedbenzene), 7.34 (1H, dd, *J*_*1*_ = 8.34 Hz, *J*_*2*_ = 1.23 Hz, benzimidazole-C_5_), 7.65 (1H, d, *J* = 8.28 Hz, benzimidazole-C_4_), 7.74 (1H, s, benzimidazole-C_7_), 8.02 (2H, d, *J* = 8.61 Hz, 1,4-disubstitutedbenzene). ^13^C NMR (75 MHz, DMSO-*d*_6_, ppm) δ: 12.35, 15.68, 37.31, 42.09, 45.91, 51.91, 52.33, 52.82, 53.01, 115.19, 116.79, 119.38, 120.14, 122.02, 128.85, 149.61, 152.01, 155.23, 155.68, 156.36, 162.97, 165.81, 169.10. HRMS (*m*/*z*): [M+2H]^+^/2 calcd for C_25_H_29_N_7_O_2_S: 246.6124; found: 246.6119.

##### 2-((4-Ethyl-5-(2-(4-hydroxyphenyl)-1H-benzo[*d*]imidazol-6-yl)-4H-1,2,4-triazol-3-yl)thio)-1-(4-phenylpiperazin-1-yl)ethanone (4b)

Yield: 78%. Mp 319.0 °C. ^1^H NMR (300 MHz, DMSO-*d*_6_, ppm) δ: 1.25 (3H, t, *J* = 7.11 Hz, CH_3_), 3.13 (2H, br.s., piperazine CH), 3.22 (2H, br.s., piperazine CH), 3.63–3.65 (4H, m, piperazine CH), 4.04–4.011 (2H, m, CH_2_), 4.39 (2H, s, −CH_2_), 6.82–6.85 (3H, m, aromatic CH), 6.97 (2H, d, *J* = 7.86 Hz, 1,4-disubstitutedbenzene), 7.21–7.26 (2H, m, aromatic CH), 7.32 (1H, dd, *J*_*1*_ = 8.22 Hz, *J*_*2*_ = 1.53 Hz, benzimidazole-C_5_), 7.62 (1H, d, *J* = 8.28 Hz, benzimidazole-C_4_), 7.71 (1H, s, benzimidazole-C_7_), 8.01 (2H, d, *J* = 8.73 Hz, 1,4-disubstitutedbenzene). ^13^C NMR (75 MHz, DMSO-*d*_6_, ppm) δ: 15.70, 37.27, 41.86, 48.60, 49.00, 51.26, 60.56, 108.45, 109.19, 111.48, 116.36, 116.54, 119.82, 121.72, 121.97, 124.20, 124.78, 128.80, 129.48, 134.20, 134.65, 135.54, 149.59, 151.34, 165.68 (C = O). HRMS (*m*/*z*): [M+2H]^+^/2 calcd for C_29_H_29_N_7_O_2_S: 270.6124; found: 270.6116.

##### 2-((4-Ethyl-5-(2-(4-hydroxyphenyl)-1H-benzo[*d*]imidazol-6-yl)-4H-1,2,4-triazol-3-yl)thio)-1-(4-(2-pyridinyl)piperazin-1-yl)ethanone (4c)

Yield: 76%. Mp 312.4 °C. ^1^H NMR (300 MHz, DMSO-*d*_6_, ppm) δ: 1.25 (3H, br.s., CH_3_), 3.60 (8H, br.s., piperazine CH), 4.08 (2H, s, CH_2_), 4.41 (2H, s, −CH_2_), 6.67–6.83 (4H, m, aromatic CH), 7.32 (1H, s, aromatic CH), 7.58–7.70 (3H, m, aromatic CH), 8.01–8.14 (3H, m, aromatic CH). ^13^C NMR (75 MHz, DMSO-*d*_6_, ppm) δ: 15.70, 37.30, 41.46, 41.72, 44.68, 45.20, 58.19, 104.53, 107.78, 109.93, 111.07, 113.98, 116.59, 119.90, 121.25, 124.58, 128.01, 128.79, 134.03, 138.14, 140.58, 143.39, 146.09, 148.05, 166.24 (C = O).

##### 2-((4-Ethyl-5-(2-(4-hydroxyphenyl)-1H-benzo[*d*]imidazol-6-yl)-4H-1,2,4-triazol-3-yl)thio)-1-(4-(2-pyrimidinyl)piperazin-1-yl)ethanone (4d)

Yield: 74%. Mp 144.0 °C. ^1^H NMR (300 MHz, DMSO-*d*_6_, ppm) δ: 1.23 (3H, t, CH_3_), 3.06–3.14 (4H, m, piperazine CH), 3.60–3.66 (4H, m, piperazine CH), 3.75 (2H, s, CH_2_), 4.38 (2H, s, −CH_2_), 6.80–6.83 (2H, m, aromatic CH), 6.96–6.99 (2H, m, aromatic CH), 7.05–7.10 (2H, m, aromatic CH), 7.60–7.63 (1H, m, aromatic CH), 7.70 (1H, s, aromatic CH), 8.00 (2H, d, *J* = 8.67 Hz, aromatic CH). ^13^C NMR (75 MHz, DMSO-*d*_6_, ppm) δ: 15.55, 37.36, 42.08, 45.90, 52.41, 52.90, 61.29, 115.27, 115.44, 115.55, 116.72, 119.55, 120.14, 122.04, 122.23, 128.84, 131.13, 131.23, 134.42, 134.46, 149.60, 156.35, 160.15, 163.37, 165.84.

##### 2-((4-Ethyl-5-(2-(4-methoxyphenyl)-1H-benzo[*d*]imidazol-6-yl)-4H-1,2,4-triazol-3-yl)thio)-1-(4-ethylpiperazin-1-yl)ethanone (4e)

Yield: 71%. Mp 112.7 °C. ^1^H NMR (300 MHz, DMSO-*d*_6_, ppm) δ: 1.01 (3H, t, *J* = 7.11 Hz, CH_3_), 1.26 (3H, t, *J* = 6.96 Hz, CH_3_), 3.46–3.52 (8H, m, piperazine CH), 3.23 (2H, s, CH_2_), 3.87 (3H, s, OCH_3_), 4.07 (2H, q, *J* = 6.97 Hz, CH_2_), 4.32 (2H, s, −CH_2_), 7.07 (2H, d, *J* = 8.91 Hz, 1,4- disubstitutedbenzene), 7.31 (1H, dd, *J*_*1*_ = 8.22 Hz, *J*_*2*_ = 1.44 Hz, benzimidazole-C_5_), 7.65 (1H, s, benzimidazole-C_4_), 7.75–7.77 (1H, m, benzimidazole-C_7_), 8.19 (2H, d, *J* = 8.85 Hz, 1,4-disubstitutedbenzene). ^13^C NMR (75 MHz, DMSO-*d*_6_, ppm) δ: 12.33, 15.70, 37.30, 42.11, 45.94, 51.92, 52.35, 52.83, 103.11, 108.40, 109.26, 114.64, 114.75, 121.56, 123.20, 128.69, 131.08, 135.98, 139.92, 151.16, 165.86 (C = O). HRMS (*m*/*z*): [M+2H]^+^/2 calcd for C_26_H_31_N_7_O_2_S: 253.6203; found: 253.6200.

##### 2-((4-Ethyl-5-(2-(4-methoxyphenyl)-1H-benzo[*d*]imidazol-6-yl)-4H-1,2,4-triazol-3-yl)thio)-1-(4-phenylpiperazin-1-yl)ethanone (4f)

Yield: 75%. Mp 153.8 °C. ^1^H NMR (300 MHz, DMSO-*d*_6_, ppm) δ: 1.31 (3H, t, *J* = 7.20 Hz, CH_3_), 3.05–3.14 (4H, m, piperazine CH), 3.51–3.55 (4H, m, piperazine CH), 3.80 (3H, s, OCH_3_), 4.63 (2H, s, −CH_2_), 5.44 (2H, s, CH_2_), 6.78–6.82 (3H, m, aromatic CH), 6.90 (1H, s, aromatic CH), 7.10–7.13 (3H, m, aromatic CH), 7.65–7.67 (2H, m, aromatic CH), 7.77–7.81 (1H, m, aromatic CH), 8.21–8.24 (2H, m, aromatic CH). ^13^C NMR (75 MHz, DMSO-*d*_6_, ppm) δ: 15.66, 30.06, 37.25, 42.14, 44.64, 45.74, 46.60, 49.00, 55.81, 114.19, 114.77, 114.85, 116.33, 116.39, 116.47, 119.60, 119.81, 122.70, 128.76, 129.48, 130.98, 137.32, 151.09, 153.11, 160.13, 161.27, 165.67.

##### 2-((4-Ethyl-5-(2-(4-methoxyphenyl)-1H-benzo[*d*]imidazol-6-yl)-4H-1,2,4-triazol-3-yl)thio)-1-(4-(2-pyridinyl)piperazin-1-yl)ethanone (4g)

Yield: 78%. Mp 204.3 °C. ^1^H NMR (300 MHz, DMSO-*d*_6_, ppm) δ: 1.26–1.29 (3H, m, CH_3_), 3.85 (4H, br.s., piperazine CH), 3.89 (4H, br.s., piperazine CH), 4.11–4.13 (2H, m, CH_2_), 4.47 (3H, s, -OCH_3_), 4.68 (2H, s, CH_2_), 7.21 (2H, d, *J* = 8.91 Hz, aromatic CH), 7.51 (1H, d, *J* = 8.37 Hz, aromatic CH), 7.63–7.66 (2H, m, aromatic CH), 7.86 (2H, d, *J* = 8.19 Hz, aromatic CH), 8.32 (2H, d, *J* = 8.70 Hz, aromatic CH), 8.47 (2H, d, *J* = 8.97 Hz, aromatic CH). ^13^C NMR (75 MHz, DMSO-*d*_6_, ppm) δ: 15.60, 37.38, 41.47, 42.51, 45.64, 54.22, 56.14, 60.68, 110.22, 112.67, 113.59, 115.05, 115.36, 118.27, 124.26, 124.76, 129.55, 130.18, 135.84, 140.99, 143.03,146.33, 150.96, 163.41, 165.15, 165.36, 166.20. HRMS (*m*/*z*): [M+2H]^+^/2 calcd for C_29_H_30_N_8_O_2_S: 278.1179; found: 278.1170.

##### 2-((4-Ethyl-5-(2-(4-methoxyphenyl)-1H-benzo[*d*]imidazol-6-yl)-4H-1,2,4-triazol-3-yl)thio)-1-(4-(2-pyrimidinyl)piperazin-1-yl)ethanone (4h)

Yield: 79%. Mp 190.5 °C. ^1^H NMR (300 MHz, DMSO-*d*_6_, ppm) δ:1.27–1.30 (3H, m, CH_3_), 3.74–3.77 (4H, m, piperazine CH), 3.82–3.87 (4H, m, piperazine CH), 3.91 (3H, s, -OCH_3_), 4.10–4.12 (2H, m, −CH_2_), 4.47 (2H, s, −CH_2_), 6.66–6.70 (2H, m, aromatic CH), 7.28 (2H, d, *J* = 9.00 Hz, aromatic CH), 7.74 (1H, dd, *J*_*1*_ = 8.40 Hz, *J*_*2*_ = 1.29 Hz, benzimidazole-C_5_), 7.95 (1H, d, *J* = 8.46 Hz, aromatic CH), 8.02 (1H, s, Aromatic CH), 8.35–0.36 (1H, m, aromatic CH), 8.40–8.41 (2H, m, aromatic CH). ^13^C NMR (75 MHz, DMSO-*d*_6_, ppm) δ: 15.52, 37.65, 41.85, 42.70, 43.42, 43.71, 45.69, 56.27, 110.64, 110.96, 111.61, 114.52, 115.08, 115.63, 122.56, 124.42, 127.12, 130.62, 135.80, 136.59, 138.43, 150.94, 158.48, 158.59, 161.36, 165.98. HRMS (*m*/*z*): [M+2H]^+^/2 calcd for C_28_H_29_N_9_O_2_S: 278.6155; found: 278.6146.

##### 2-((4-Ethyl-5-(2-(4-ethoxyphenyl)-1H-benzo[*d*]imidazol-6-yl)-4H-1,2,4-triazol-3-yl)thio)-1-(4-ethylpiperazin-1-yl)ethanone (4i)

Yield: 70%. Mp 144.3 °C. ^1^H NMR (300 MHz, DMSO-*d*_6_, ppm) δ: 1.30–1.34 (6H, m, CH_3_), 1.37–1.42 (3H, m, CH_3_), 3.45 (4H, br.s., piperazine CH), 3.49 (4H, br.s., piperazine CH), 4.13–4.17 (2H, m, CH_2_), 4.22–4.29 (2H, m, CH_2_), 4.41–4.43 (2H, m, CH_2_), 4.57 (2H, s, −CH_2_), 7.25–7.28 (2H, m, aromatic CH), 7.74–7.77 (1H, m, aromatic CH), 7.95 (1H, d, *J* = 8.43 Hz, aromatic CH), 8.03–8.07 (1H, m, aromatic CH), 8.47–8.52 (2H, m, aromatic CH). ^13^C NMR (75 MHz, DMSO-*d*_6_, ppm) δ: 13.84, 15.05, 15.54, 38.95, 42.41, 45.67, 47.36, 50.01, 50.42, 50.98, 51.09, 114.29, 115.93, 117.62, 119.59, 123.64, 125.20, 127.28, 130.67, 132.89, 137.46, 139.44, 142.14, 147.54, 165.31 (C = O). HRMS (*m*/*z*): [M+2H]^+^/2 calcd for C_27_H_33_N_7_O_2_S: 260.6281; found: 260.6272.

##### 2-((4-Ethyl-5-(2-(4-ethoxyphenyl)-1H-benzo[*d*]imidazol-6-yl)-4H-1,2,4-triazol-3-yl)thio)-1-(4-phenylpiperazin-1-yl)ethanone (4j)

Yield: 69%. Mp 118.8 °C. ^1^H NMR (300 MHz, DMSO-*d*_6_, ppm) δ: 1.22–1.27 (3H, m, CH_3_), 1.33–1.37 (3H, m, CH_3_), 3.09–3.14 (4H, m, piperazine CH), 3.18–3.23 (4H, m, piperazine CH), 4.10–4.12 (4H, m, CH_2_), 4.40 (2H, s, −CH_2_), 6.80–6.82 (1H, m, aromatic CH), 6.93–6.96 (2H, m, aromatic CH), 7.08–7.11 (2H, m, aromatic CH), 7.19–7.22 (2H, m, aromatic CH), 7.41–7.44 (1H, m, aromatic CH), 7.71–7.74 (1H, m, aromatic CH), 7.82 (1H, s, aromatic CH), 8.22–8.24 (2H, m, aromatic CH). ^13^C NMR (75 MHz, DMSO-*d*_6_, ppm) δ: 15.06, 15.66, 37.26, 41.93, 44.11, 45.69, 48.60, 49.06, 63.80, 115.28, 116.33, 116.54, 119.80, 119.92, 121.04, 122.42, 122.81, 128.31, 128.92, 129.46, 130.04, 149.80, 151.15, 153.48, 156.12, 160.70, 165.95. HRMS (*m*/*z*): [M+2H]^+^/2 calcd for C_31_H_33_N_7_O_2_S: 284.6281; found: 284.6280.

##### 2-((4-Ethyl-5-(2-(4-ethoxyphenyl)-1H-benzo[*d*]imidazol-6-yl)-4H-1,2,4-triazol-3-yl)thio)-1-(4-(2-pyridinyl)piperazin-1-yl)ethanone (4k)

Yield: 80%. Mp 162.2 °C. ^1^H NMR (300 MHz, DMSO-*d*_6_, ppm) δ: 1.24–1.29 (3H, m, CH_3_), 1.36–1.40 (3H, m, CH_3_), 3.72–3.78 (8H, m, piperazine CH), 4.14–4.19 (2H, m, CH_2_), 4.45 (2H, s, CH_2_), 4.91 (2H, s, −CH_2_), 7.11–7.15 (1H, m, aromatic CH), 7.21–7.24 (3H, m, aromatic CH), 7.65–7.68 (1H, m, aromatic CH), 7.87–7.90 (2H, m, aromatic CH), 7.96 (1H, s, aromatic CH), 8.26 (1H, d, *J* = 8.70 Hz, aromatic CH), 8.37 (2H, d, *J* = 8.82 Hz, aromatic CH). ^13^C NMR (75 MHz, DMSO-*d*_6_, ppm) δ: 14.99, 15.66, 38.40, 41.46, 45.19, 45.71, 47.91, 52.90, 58.83, 109.93, 110.29, 113.57, 113.67, 114.80, 115.11, 115.47, 116.78, 116.99, 118.86, 121.01, 121.32, 124.18, 124.68, 130.16, 140.34, 140.91, 163.03, 166.20. HRMS (*m*/*z*): [M+2H]^+^/2 calcd for C_30_H_32_N_8_O_2_S: 285.1257; found: 285.1260.

##### 2-((4-Ethyl-5-(2-(4-ethoxyphenyl)-1H-benzo[*d*]imidazol-6-yl)-4H-1,2,4-triazol-3-yl)thio)-1-(4-(2-pyrimidinyl)piperazin-1-yl)ethanone (4l)

Yield: 71%. Mp 140.8 °C. ^1^H NMR (300 MHz, DMSO-*d*_6_, ppm) δ:1.26–1.28 (3H, m, CH_3_), 1.36–1.41 (3H, m, CH_3_), 3.53–3.57 (8H, m, piperazine CH), 3.96–3.99 (4H, m, CH_2_), 4.52 (2H, s, −CH_2_), 6.73–6.76 (1H, m, aromatic CH), 7.28 (2H, d, *J* = 9.03 Hz, aromatic CH), 7.80 (1H, dd, *J*_*1*_ = 8.49 Hz, *J*_*2*_ = 1.44 Hz, benzimidazole-C_5_), 7.98 (2H, d, *J* = 8.52 Hz, aromatic CH), 8.08 (1H, s, Aromatic CH), 8.36–8.38 (1H, m, aromatic CH), 8.49–8.52 (3H, m, aromatic CH). ^13^C NMR (75 MHz, DMSO-*d*_6_, ppm) δ: 14.92, 15.36, 41.82, 42.42, 42.74, 43.45, 43.77, 45.67, 64.40, 110.94, 111.58, 114.09, 114.94, 116.04, 123.54, 125.10, 126.35, 130.94, 132.89, 137.98,154.53, 158.39, 158.55, 160.85, 163.22, 165.86. HRMS (*m*/*z*): [M+2H]^+^/2 calcd for C_29_H_31_N_9_O_2_S: 285.6177; found: 285.6174.

### Cell Viability Assay

The anticancer activity of compounds **4a**–**l** were screened according to the MTT assays. The MTT assays were performed as previously described.^3^ Anticancer activity of final compounds was assessed against five different cancer cell lines A549 (lung carcinoma cell line) and C6 (rat glioma cell line) cell lines. Doxorubicin was used as the reference drugs in the MTT assays.

### DNA Topoisomerase I Assay

The purpose of this investigation was to ascertain if produced substances exhibited topoisomerase I inhibition using the topoisomerase I assay kit (TG1018–2; TopoGen). Using agarose gel electrophoresis, the topoisomerase I inhibitory activities of the final compounds were assessed by measuring the relaxation of supercoiled plasmid DNA. A positive control, camptothecin, was used. A final volume of 20 μL reaction volume was used for the experiment, which contained 2 μL of 10× TGS buffer, 6 μL of water, 2 μL of supercoiled plasmid DNA, 2 μL of the test compound, 2 μL of Topo I, 2 μl of 10% SDS, 2 μL of proteinase K, and 2 μL of the DNA loading dye. Following a 30 min incubation period at 37 °C, the reaction mixtures were electrophoresed using 1× TAE buffer for 75 min at a potential of 50 V on a 1% agarose gel.^25^

### Molecular Docking

Molecular docking simulations were carried out using Maestro version 13.3, which is part of the Schrödinger suite. The three-dimensional crystallographic structure of human DNA topoisomerase I (PDB ID: 1T8I) served as the macromolecular target for our study.^26^ The protein was prepared for docking using the Protein Preparation Wizard, where hydrogen atoms were added consistent with a pH of 7.0, and all water molecules were removed. The binding site was defined based on the cocrystallized ligand camptothecin position, allowing for a comprehensive exploration of the active pocket. The ligand 3D structures of **4b** and **4h** were created using the built-in LigPrep utility, which generated three-dimensional geometries and possible tautomeric states. Subsequent docking was executed using the Glide Ligand Docking module, with the standard precision (SP) mode to scan for potential binding orientations.^29^ Postdocking, the visualization, and analysis of the docked complexes were conducted using PyMOL version 2.4. This molecular visualization tool enabled us to render representations of the docking poses and to dissect the molecular interactions, including hydrogen bonds, hydrophobic contacts, and pi-interactions. The binding poses were visually compared with the known inhibitor camptothecin to assess the fidelity of the docking process and to infer the potential efficacy of the compounds. The maximum common structure RMSD values for the docking simulations were calculated by superimposing the docked ligands onto the cocrystallized ligand structure.

### Molecular Dynamics

Molecular Dynamics Simulation (MDS) serves as a valuable tool for assessing the structural stability and flexibility of protein–ligand complexes.^30^ In this study, we employed MDS to validate the Protein–Ligand Complex (PLC) involving the top-hit compound and to evaluate the ligand’s binding stability within the active site of the chosen target protein. For our MDS, we utilized the Desmond module within the Schrodinger Suite, which was developed by the D.E. Shaw research group under an academic license.^31^ Our simulation setup involved the creation of an orthorhombic simulation box using Simple Point-Charge (SPC) water molecules, with periodic boundary conditions extending 10 Å from the protein’s surface.^32^ We solvated the system using the TIP3P water model and added counterions to maintain neutrality. An isosmotic state was preserved by introducing 0.15 M NaCl into the system. The OPLS_AA force field was applied to the protein–ligand complex, and energy minimization was conducted until system stability was achieved (via 1000 steps of steepest descent and the conjugate gradient algorithm).^33,34^ Subsequently, the equilibrated system underwent a 300 ns Molecular Dynamics Simulation at a temperature of 310.15 K and a pressure of 1.0 bar, employing the NPT (isothermal–isobaric ensemble).^35^ The simulation results underwent comprehensive analysis, including the generation of a simulation interaction diagram, evaluation of Root Mean Square Deviation (RMSD) and Root Mean Square Fluctuation (RMSF), examination of protein–ligand interaction diagrams, identification of amino acid residues involved in ligand interactions in each trajectory frame, and examination of various ligand properties’ trajectories. After simulation, Molecular Mechanics/Generalized Born Surface Area (MM-GBSA) analysis was performed using the thermal_MMGBSA.py script from the Prime/Desmond module.^36^ This analysis encompassed free binding energy calculations, focusing on 200 frames within the 300 ns MDS data and yielding binding free energies in kcal/mol. In summary, our study leveraged Molecular Dynamics Simulation and MM-GBSA analysis to comprehensively evaluate the stability and interactions of a protein–ligand complex, providing insights into binding free energies for potential pharmaceutical applications.

### Quantum Mechanical Calculations

It is a necessity to determine the correct molecular structure corresponding to the minimum energy state in the structure–activity relationship; therefore, the DFT method was used in the geometry optimization stage of all structures. DFT calculations were performed using the Gaussian 09 program^37^ with the B3LYP exchange correlation functional with the 6-31G(d,p) basis set. The GaussView 5.0 program was used to create the input geometries and visualize the results.

## Supporting Information Available

^1^H NMR, ^13^C NMR, and HRMS spectra of compounds **4a**–**4l** (PDF)
