# Supplementary material for: New Benzimidazole-Triazole Derivatives as Topoisomerase I Inhibitors: Design, Synthesis, Anticancer Screening, and Molecular Modeling Studies
Source: ACS Omega. 2024 Mar 6;9(11):13359–72. doi: 10.1021/acsomega.3c10345 (PMC10955584; doi:10.1021/acsomega.3c10345)
Supplement: Supplementary file 1 — ao3c10345_si_001.pdf [file ao3c10345_si_001.pdf]

New benzimidazole-triazole derivatives as topoisomerase I inhibitors: Design, synthesis, anticancer screening, and molecular modeling studies

Ulviye Acar Çevik[1*], Betül Kaya[2], Ismail Celik[3], Mithun Rudrapal[4], Gourav Rakshit[5], Arzu Karayel[6], Serkan Levent[1], Derya Osmaniye[1], Begüm Nurpelin Sağlık[1], Merve Baysal[7], Özlem Atlı Eklioğlu[7], Yusuf Özkay[1], Zafer Asım Kaplancıklı[1]

[1] Department of Pharmaceutical Chemistry, Faculty of Pharmacy, Anadolu University, Eskişehir 26470, Turkey

[2] Department of Pharmaceutical Chemistry, Faculty of Pharmacy, Zonguldak Bülent Ecevit University, Zonguldak, Turkey

[3] Department of Pharmaceutical Chemistry, Faculty of Pharmacy, Erciyes University, Kayseri 38039, Turkey

[4] Department of Pharmaceutical Sciences, School of Biotechnology and Pharmaceutical Sciences, Vignan's Foundation for Science, Technology & Research (Deemed to Be University), Guntur, 522213, India.

[5] Department of Pharmaceutical Sciences & Technology, Birla Institute of Technology, Ranchi 835215, India.

[6] Department of Physics, Faculty of Arts and Science, Hitit University, 19030 Çorum, Turkey.

[7] Department of Pharmaceutical Toxicology, Faculty of Pharmacy, Anadolu University, Eskişehir 26470, Turkey

*Correspondence:

E-mail: uacar@anadolu.edu.tr; Tel. +90-222-335-0580/3775

Address: Anadolu University, Faculty of Pharmacy, Department of Pharmaceutical Chemistry, Eskişehir, Turkey

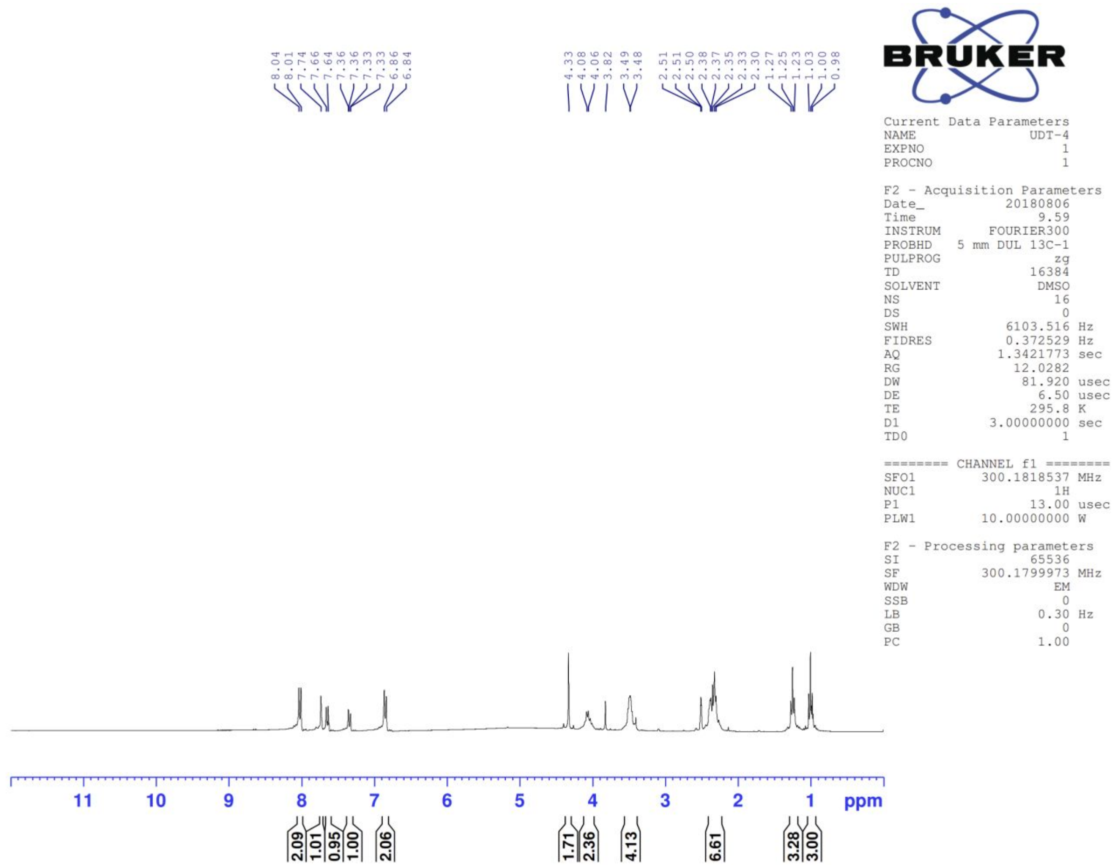

Figure S1. ^{1}H-NMR spectrum of compound **4a**

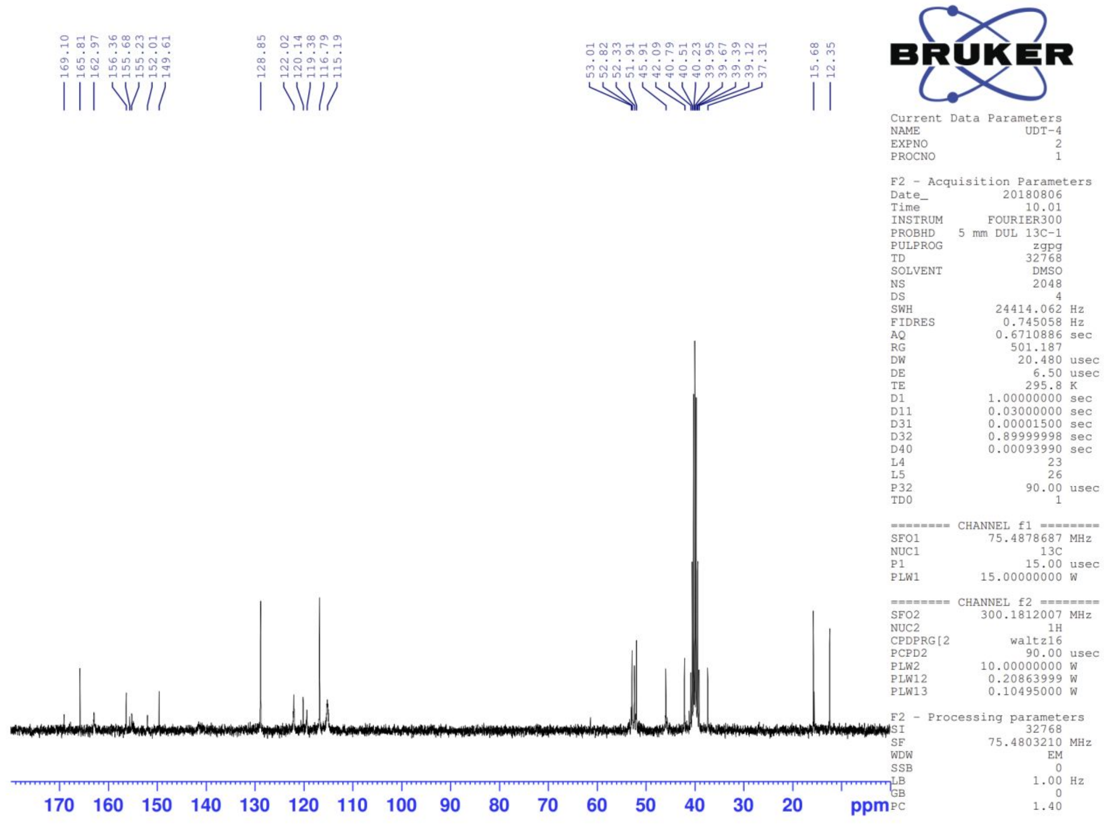

Figure S2. ^{13}C-NMR spectrum of compound **4a**

Data File: C:\LabSolutions\Data\Analiz\uac\UDT-4_4.lcd

Elmt	Val.	Min	Max	Elmt	Val.	Min	Max	Elmt	Val.	Min	Max	Elmt	Val.	Min	Max	Use Adduct
H	1	5	35	O	2	0	5	S	2	1	1	Ru	2	0	0	H
C	4	5	35	F	1	0	0	Cl	1	0	0	Pd	2	0	0	
N	3	0	8	P	3	0	0	Br	1	0	0	I	3	0	0	

Error Margin (ppm):	5	DBE Range:	0.0 - 20.0	Electron Ions:	both
HC Ratio:	unlimited	Apply N Rule:	yes	Use MSn Info:	yes
Max Isotopes:	3	Isotope RI (%):	1.00	Isotope Res:	9000
MSn Iso RI (%):	10.00	MSn Logic Mode:	AND	Max Results:	150

Event#: 1 MS(E+) Ret. Time : 1.187 Scan# : 179

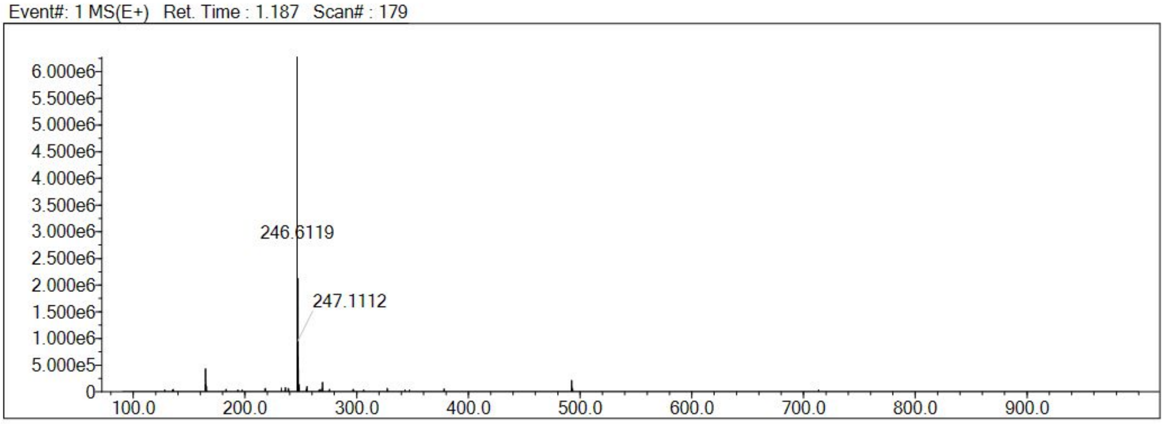

Measured region for 246.6119 m/z

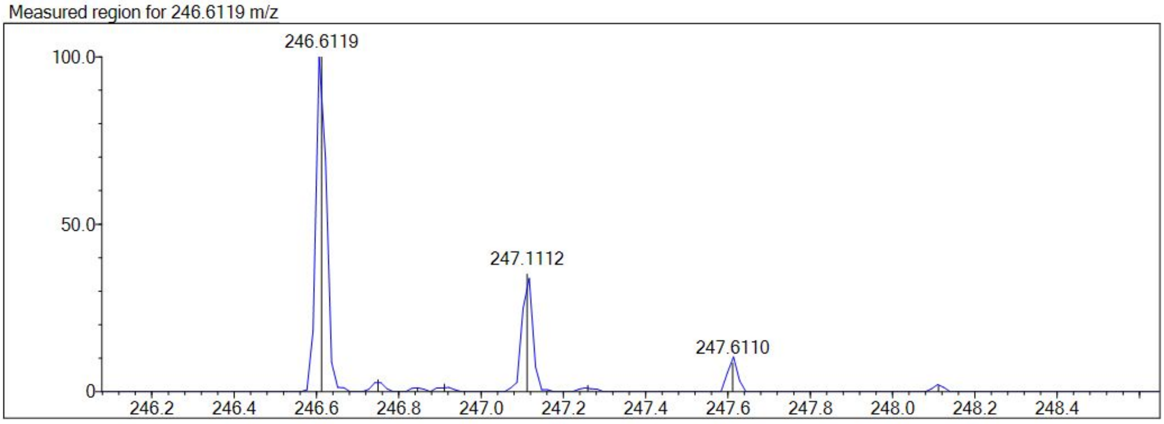

C25 H29 N7 O2 S [M+2H]2+ : Predicted region for 246.6124 m/z

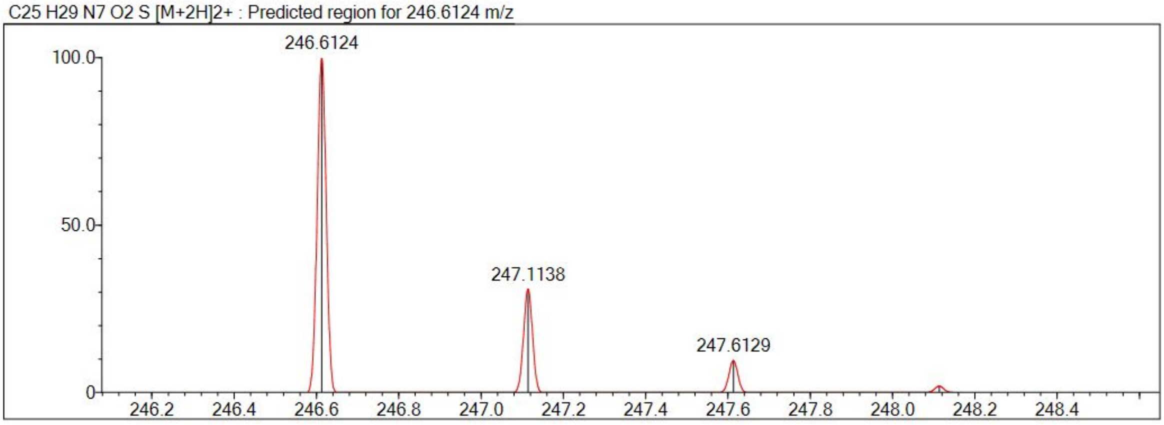

Rank	Score	Formula (M)	Ion	Meas. m/z	Pred. m/z	Df. (mDa)	Df. (ppm)	Iso	DBE
1	67.34	C25 H29 N7 O2 S	[M+2H]2+	246.6119	246.6124	-0.5	-2.03	69.12	15.0

Figure S3. Mass spectrum of compound **4a**

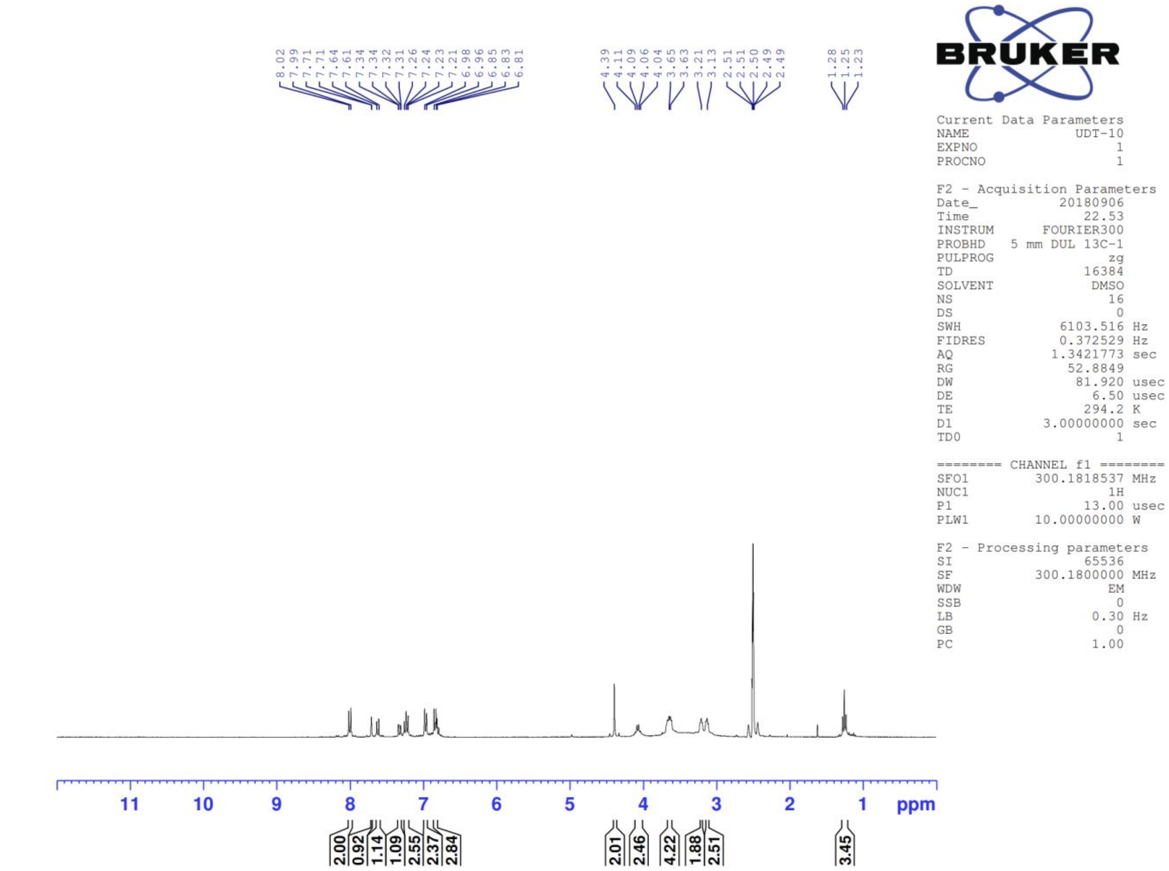

Figure S4. ^{1}H-NMR spectrum of compound **4b**

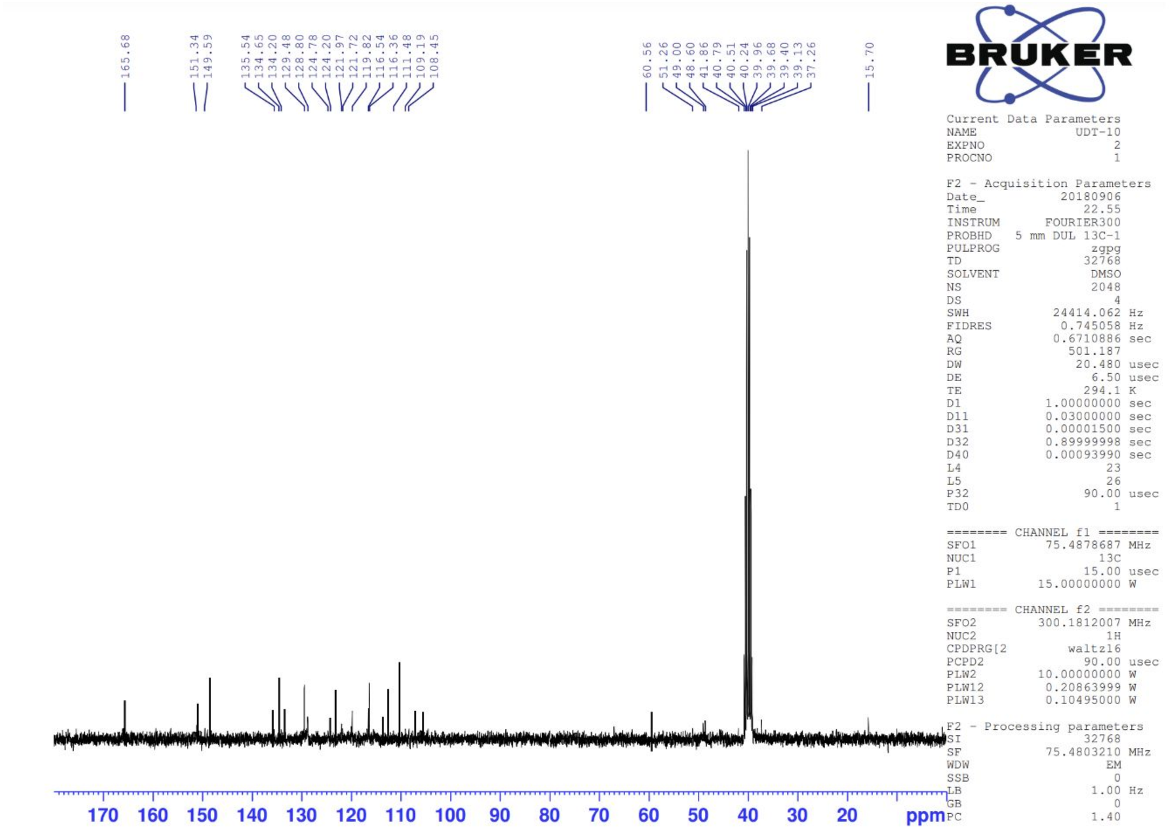

Figure S5. ^{13}C-NMR spectrum of compound **4b**

Formula Predictor Report - UDT-10_7.lcd Page 1 of 1

Data File: C:\LabSolutions\Data\Analiz\uac\UDT-10_7.lcd

Elmt	Val.	Min	Max	Elmt	Val.	Min	Max	Elmt	Val.	Min	Max	Elmt	Val.	Min	Max	Use Adduct
H	1	5	35	O	2	0	5	S	2	0	1	Ru	2	0	0	H
C	4	5	35	F	1	0	0	Cl	1	0	0	Pd	2	0	0	
N	3	0	8	P	3	0	0	Br	1	0	0	I	3	0	0	

Error Margin (ppm):	5	DBE Range:	0.0 - 20.0	Electron Ions:	both
HC Ratio:	unlimited	Apply N Rule:	yes	Use MSn Info:	yes
Max Isotopes:	3	Isotope RI (%):	1.00	Isotope Res:	9000
MSn Iso RI (%):	10.00	MSn Logic Mode:	AND	Max Results:	150

Event#: 1 MS(E+) Ret. Time : 4.427 Scan# : 665

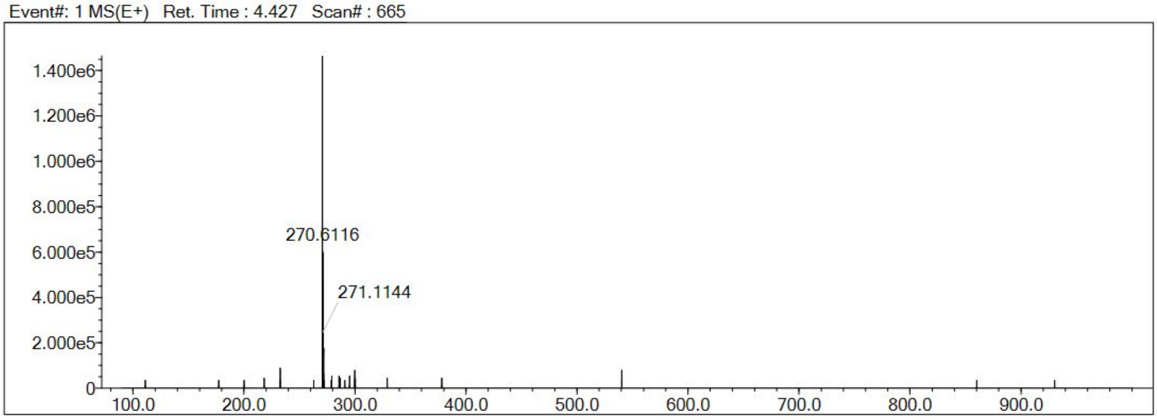

Measured region for 270.6116 m/z

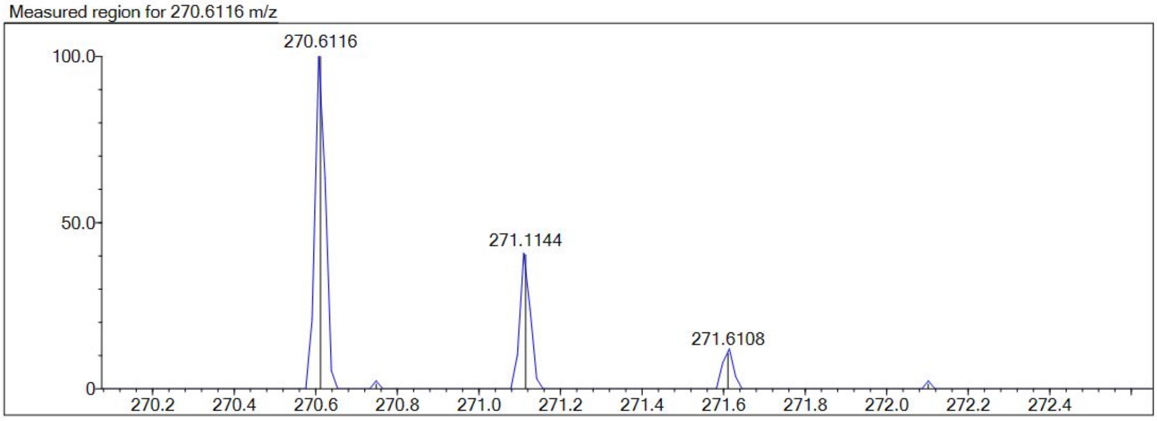

C29 H29 N7 O2 S [M+2H]2+ : Predicted region for 270.6124 m/z

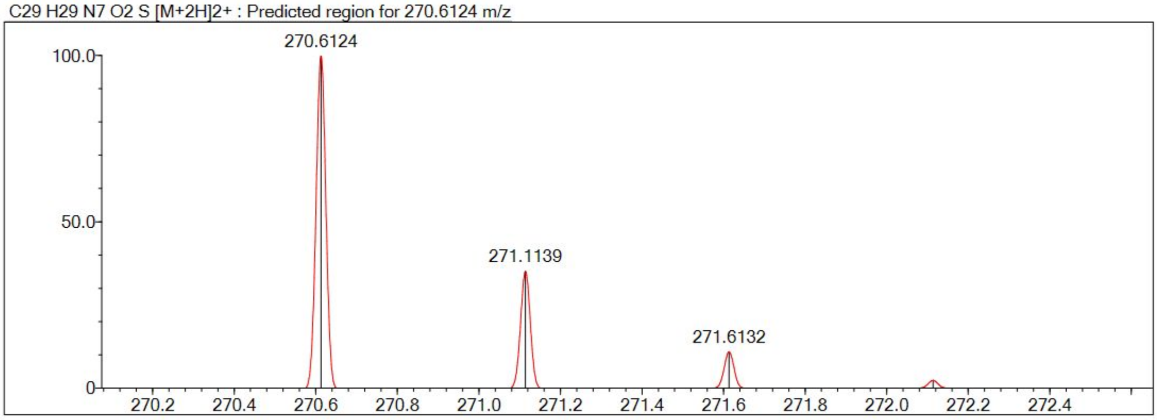

Rank	Score	Formula (M)	Ion	Meas. m/z	Pred. m/z	Df. (mDa)	Df. (ppm)	Iso	DBE
1	39.92	C29 H29 N7 O2 S	[M+2H]2+	270.6116	270.6124	-0.8	-2.96	41.97	19.0

Figure S6. Mass spectrum of compound **4b**

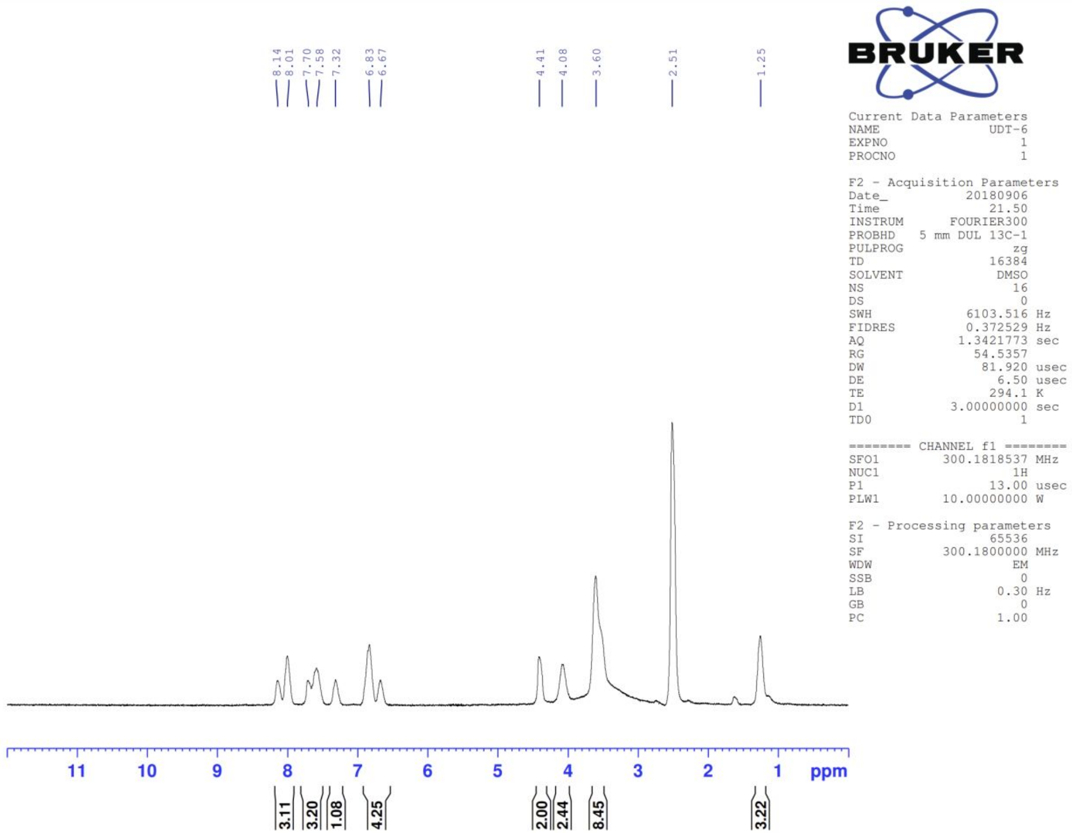

Figure S7. ^{1}H-NMR spectrum of compound **4c**

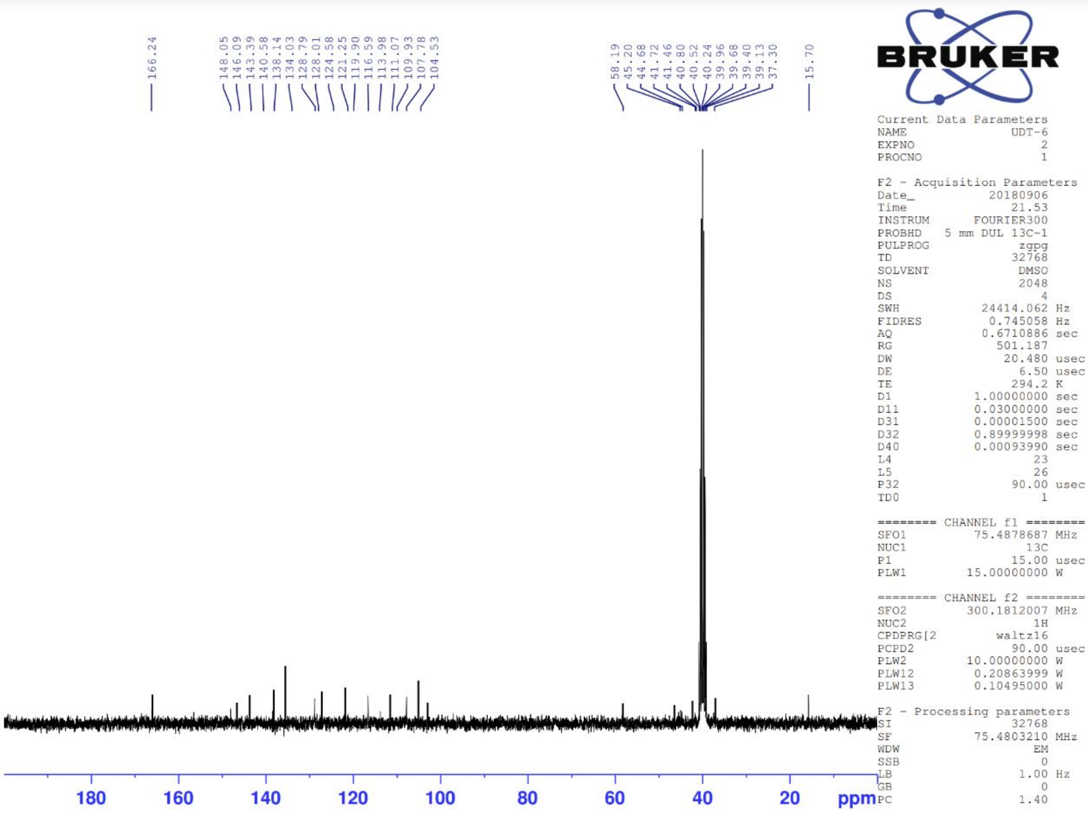

Figure S8. ^{13}C-NMR spectrum of compound **4c**

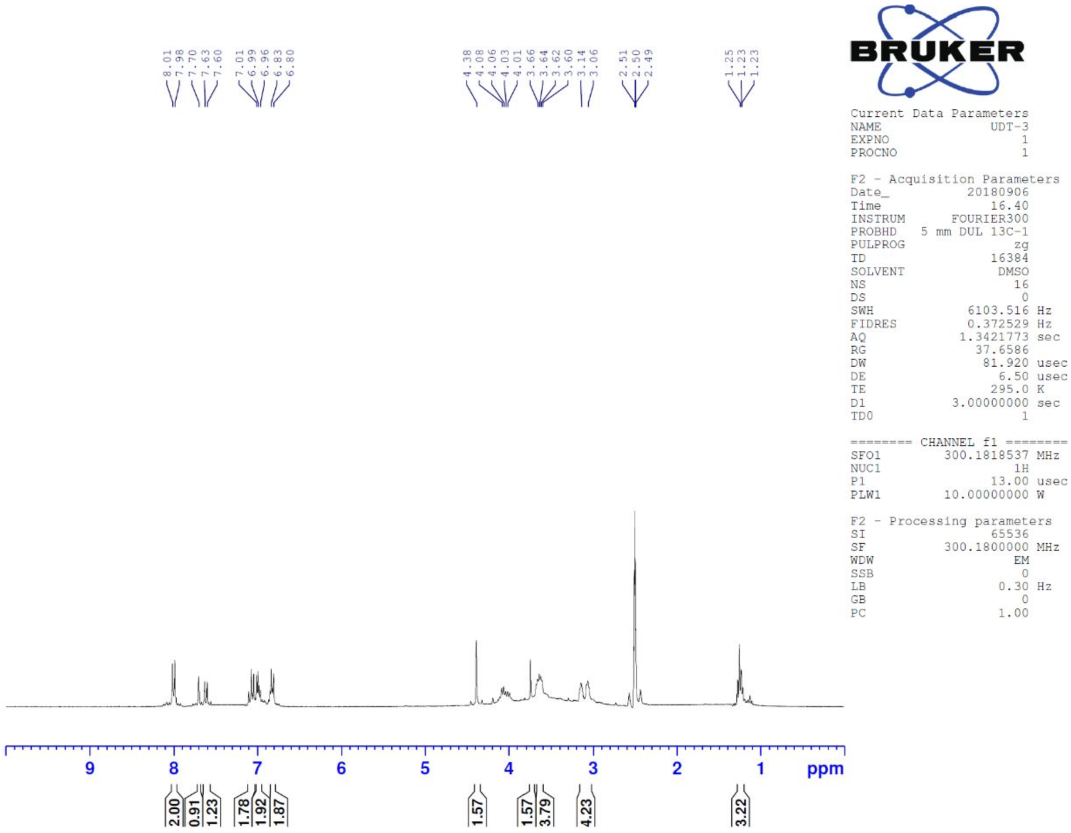

Figure S9. ^{1}H-NMR spectrum of compound **4d**

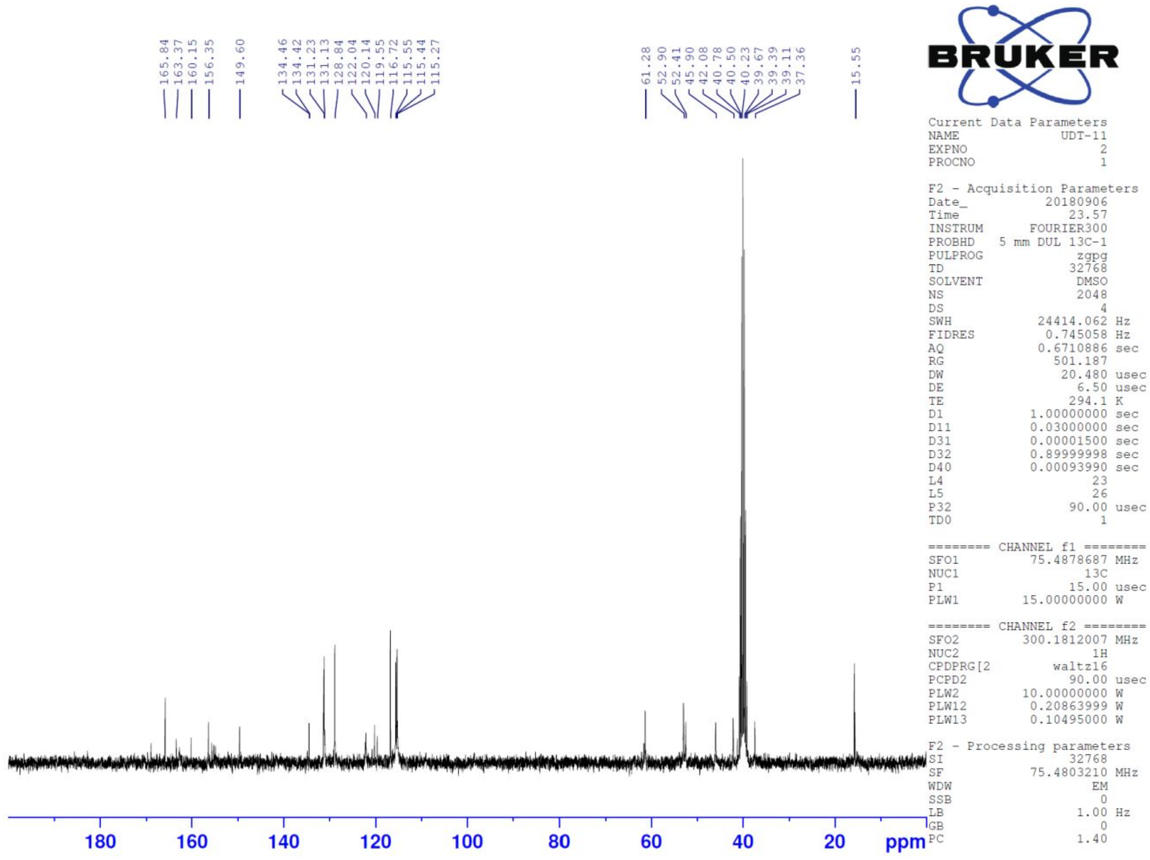

Figure S10. ^{13}C-NMR spectrum of compound **4d**

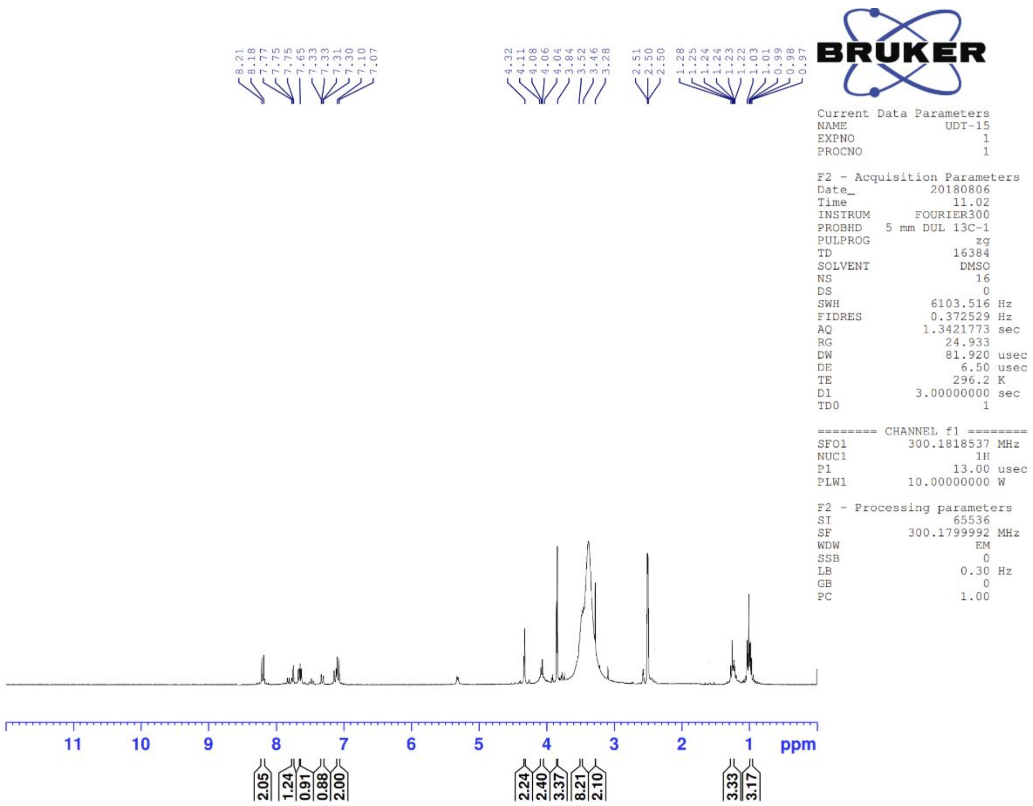

Figure S11. ^{1}H-NMR spectrum of compound **4e**

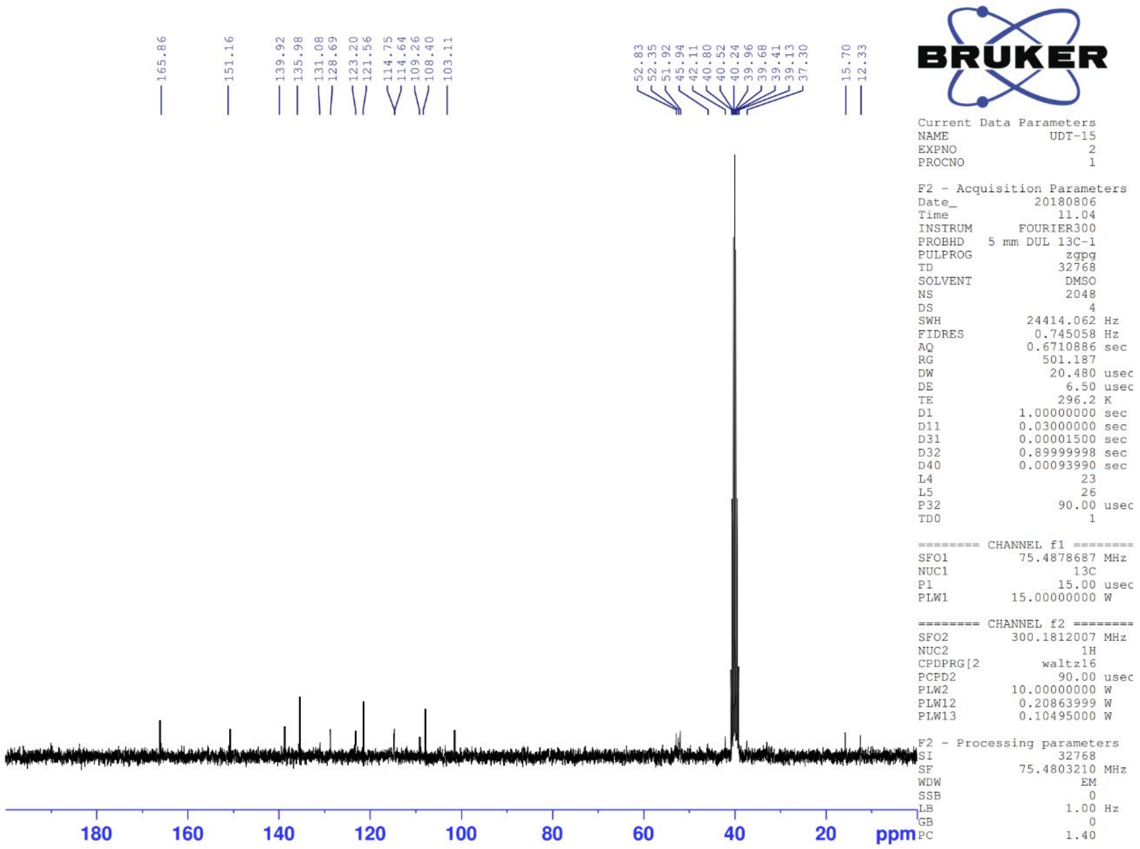

Figure S12. ^{13}C-NMR spectrum of compound **4e**

Data File: C:\LabSolutions\Data\Analiz\uac\UDT-15_8.lcd

Elmt	Val.	Min	Max	Elmt	Val.	Min	Max	Elmt	Val.	Min	Max	Elmt	Val.	Min	Max	Use Adduct
H	1	5	40	O	2	0	5	S	2	1	1	Ru	2	0	0	H
C	4	5	35	F	1	0	0	Cl	1	0	0	Pd	2	0	0	
N	3	0	8	P	3	0	0	Br	1	0	0	I	3	0	0	

Error Margin (ppm):	5	DBE Range:	0.0 - 30.0	Electron Ions:	both
HC Ratio:	unlimited	Apply N Rule:	yes	Use MSn Info:	yes
Max Isotopes:	3	Isotope RI (%):	1.00	Isotope Res:	9000
MSn Iso RI (%):	10.00	MSn Logic Mode:	AND	Max Results:	150

Event#: 1 MS(E+) Ret. Time : 2.160 Scan# : 325

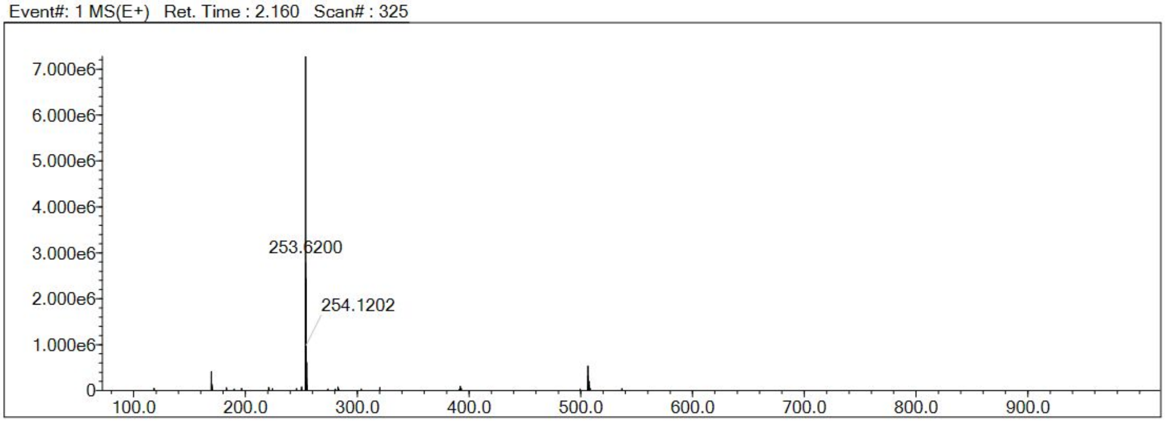

Measured region for 253.6200 m/z

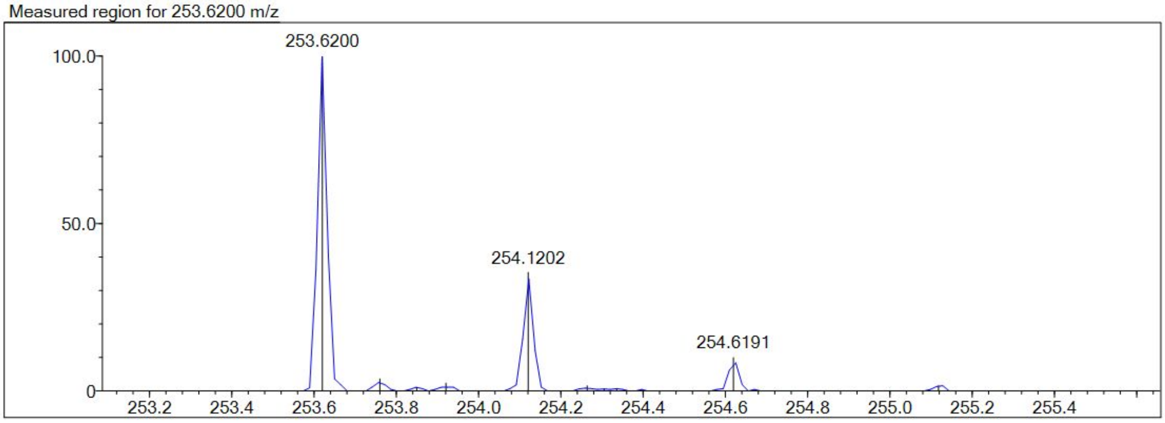

C26 H31 N7 O2 S [M+2H]2+ : Predicted region for 253.6203 m/z

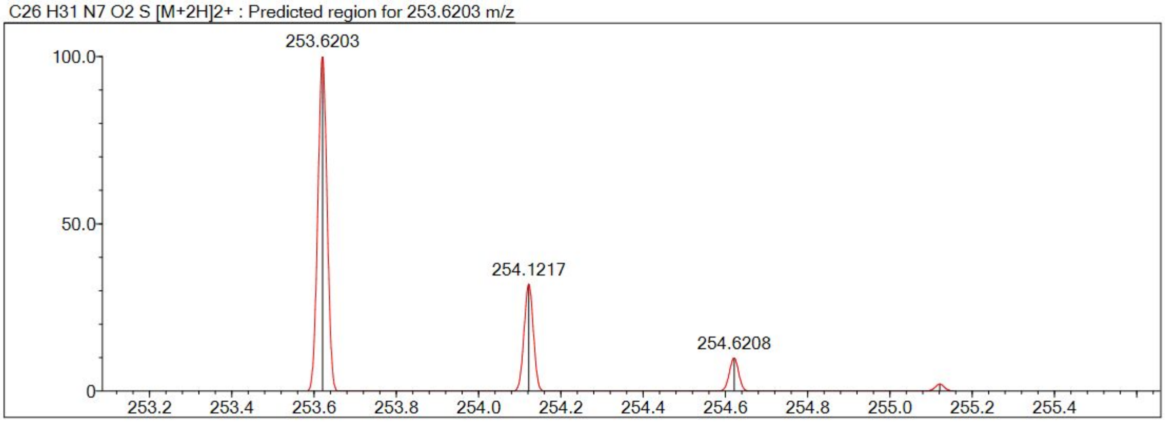

Rank	Score	Formula (M)	Ion	Meas. m/z	Pred. m/z	Df. (mDa)	Df. (ppm)	Iso	DBE
1	89.43	C26 H31 N7 O2 S	[M+2H]2+	253.6200	253.6203	-0.3	-1.18	89.84	15.0

Figure S13. Mass spectrum of compound **4e**

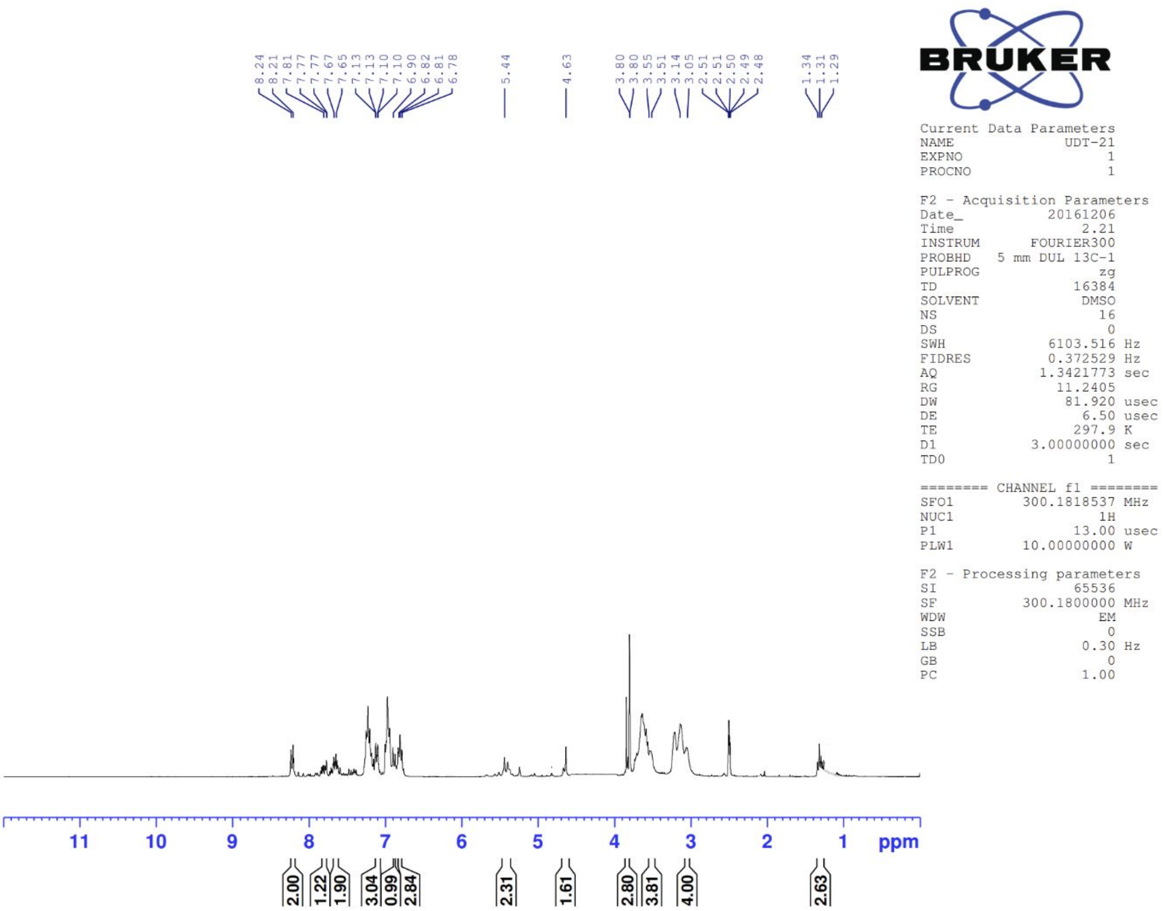

Figure S14. ^{1}H-NMR spectrum of compound **4f**

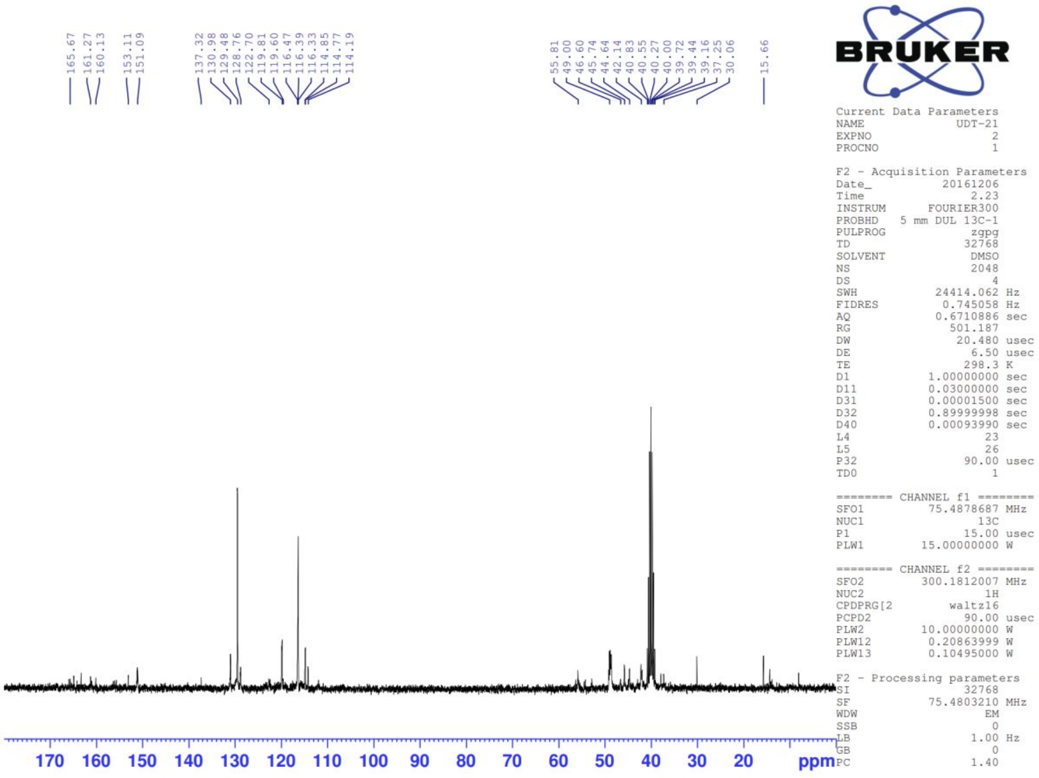

Figure S15. ^{13}C-NMR spectrum of compound **4f**

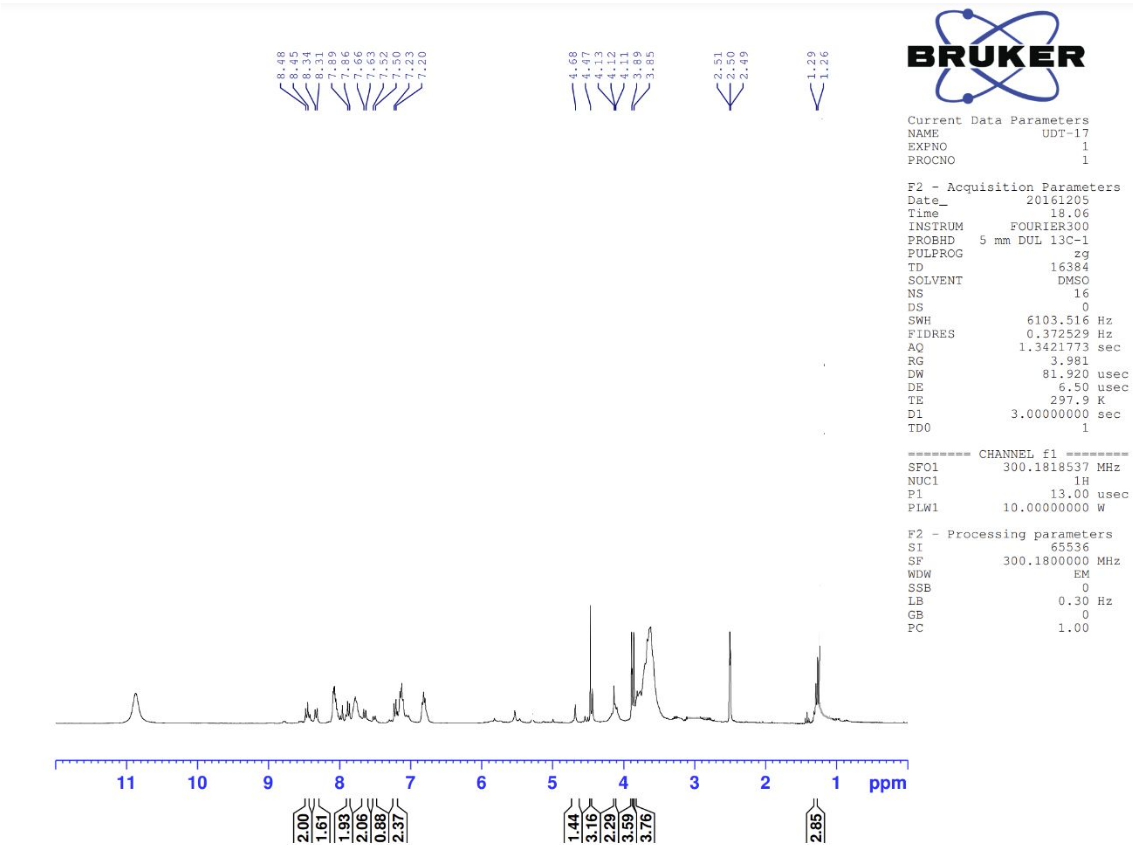

Figure S16. ^{1}H-NMR spectrum of compound **4g**

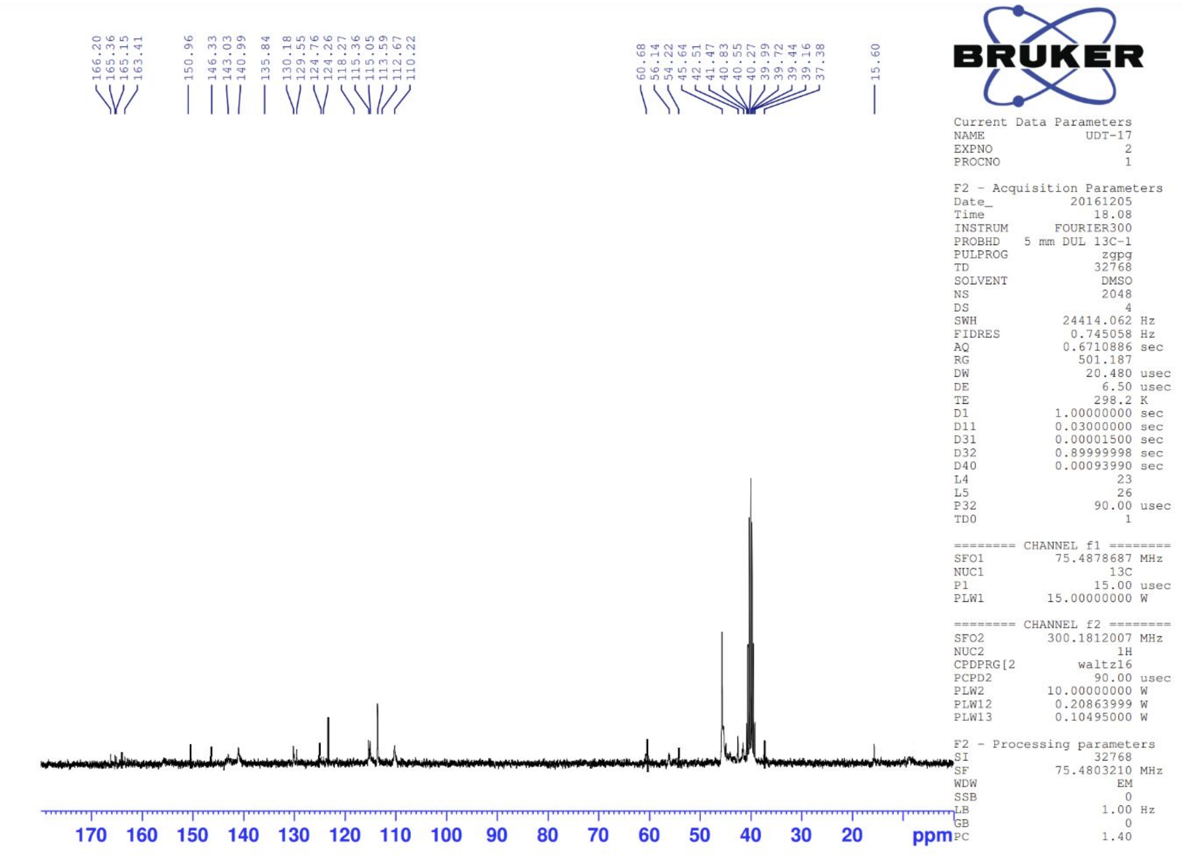

Figure S17. ^{13}C-NMR spectrum of compound **4g**

Data File: C:\LabSolutions\Data\Analiz\uac\UDT-17_9.lcd

Elmt	Val.	Min	Max	Elmt	Val.	Min	Max	Elmt	Val.	Min	Max	Elmt	Val.	Min	Max	Use Adduct
H	1	5	40	O	2	0	5	S	2	1	1	Ru	2	0	0	H
C	4	5	35	F	1	0	0	Cl	1	0	0	Pd	2	0	0	
N	3	0	8	P	3	0	0	Br	1	0	0	I	3	0	0	

Error Margin (ppm):	5	DBE Range:	0.0 - 30.0	Electron Ions:	both
HC Ratio:	unlimited	Apply N Rule:	yes	Use MSn Info:	yes
Max Isotopes:	3	Isotope RI (%):	1.00	Isotope Res:	9000
MSn Iso RI (%):	10.00	MSn Logic Mode:	AND	Max Results:	150

Event#: 1 MS(E+) Ret. Time : 2.773 -> 3.267 Scan# : 417 -> 491

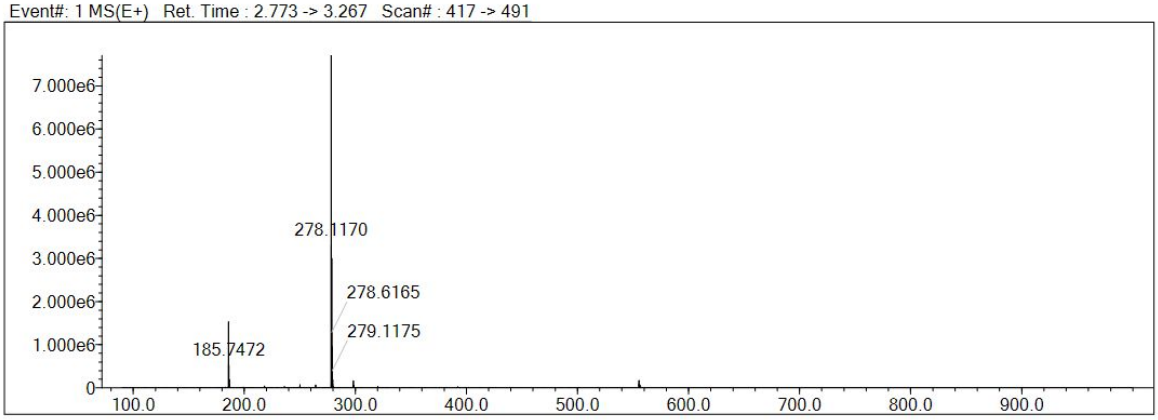

Measured region for 278.1170 m/z

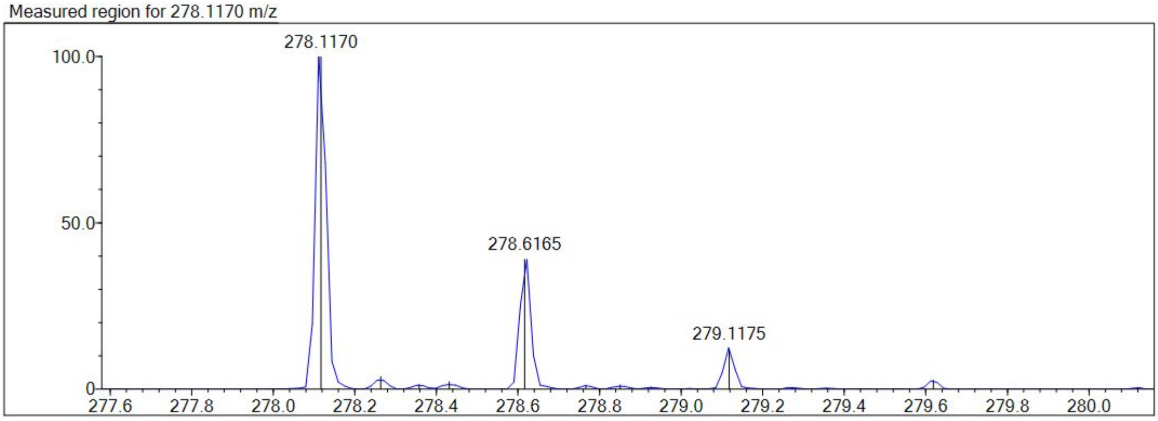

C29 H30 N8 O2 S [M+2H]2+ : Predicted region for 278.1179 m/z

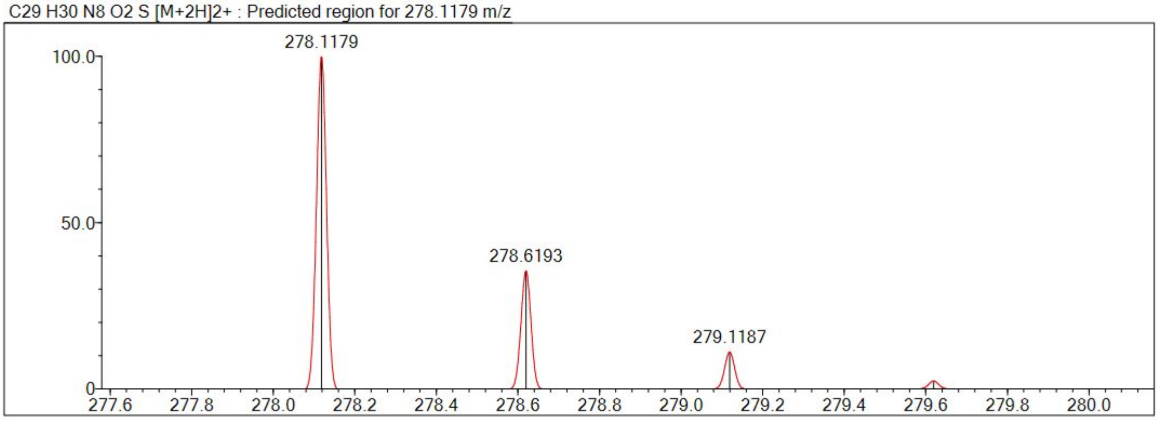

Rank	Score	Formula (M)	Ion	Meas. m/z	Pred. m/z	Df. (mDa)	Df. (ppm)	Iso	DBE
1	62.10	C29 H30 N8 O2 S	[M+2H]2+	278.1170	278.1179	-0.9	-3.24	65.79	19.0

Figure S18. Mass spectrum of compound **4g**

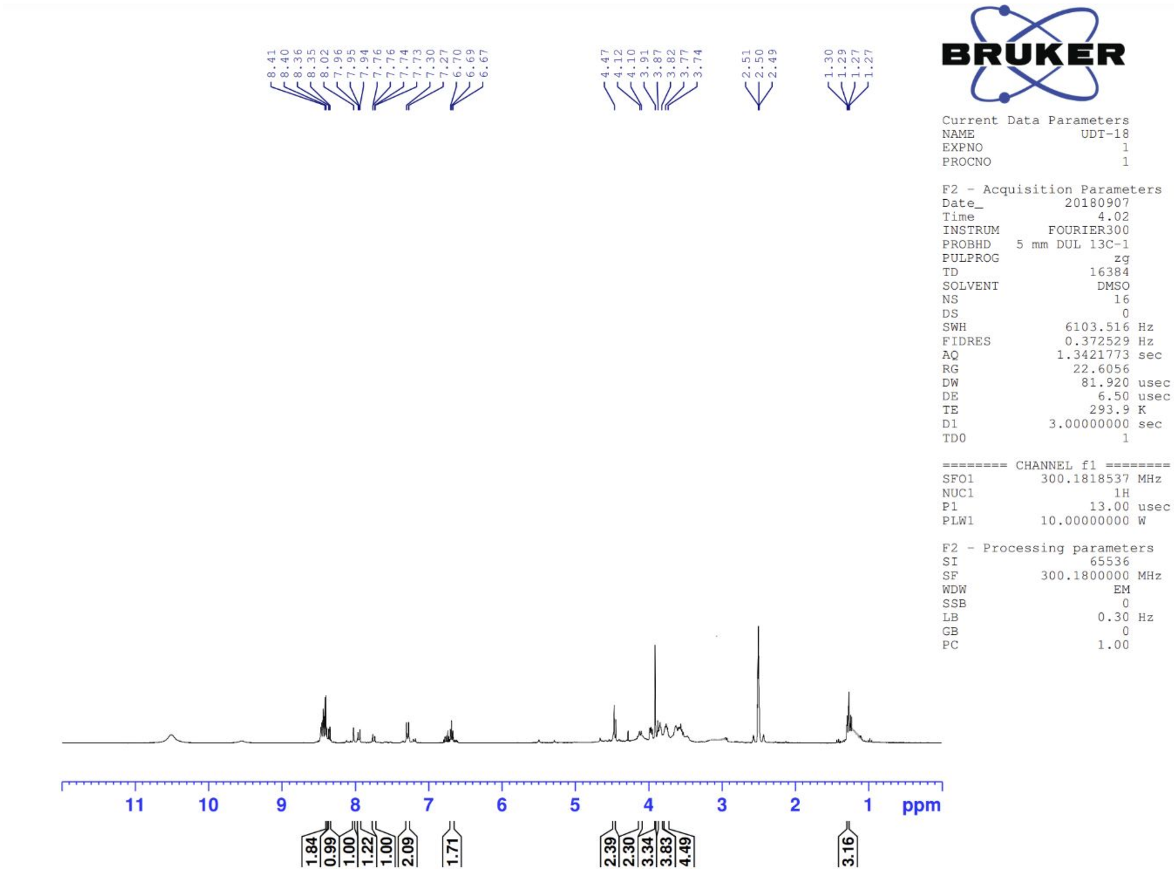

Figure S19. ^{1}H-NMR spectrum of compound **4h**

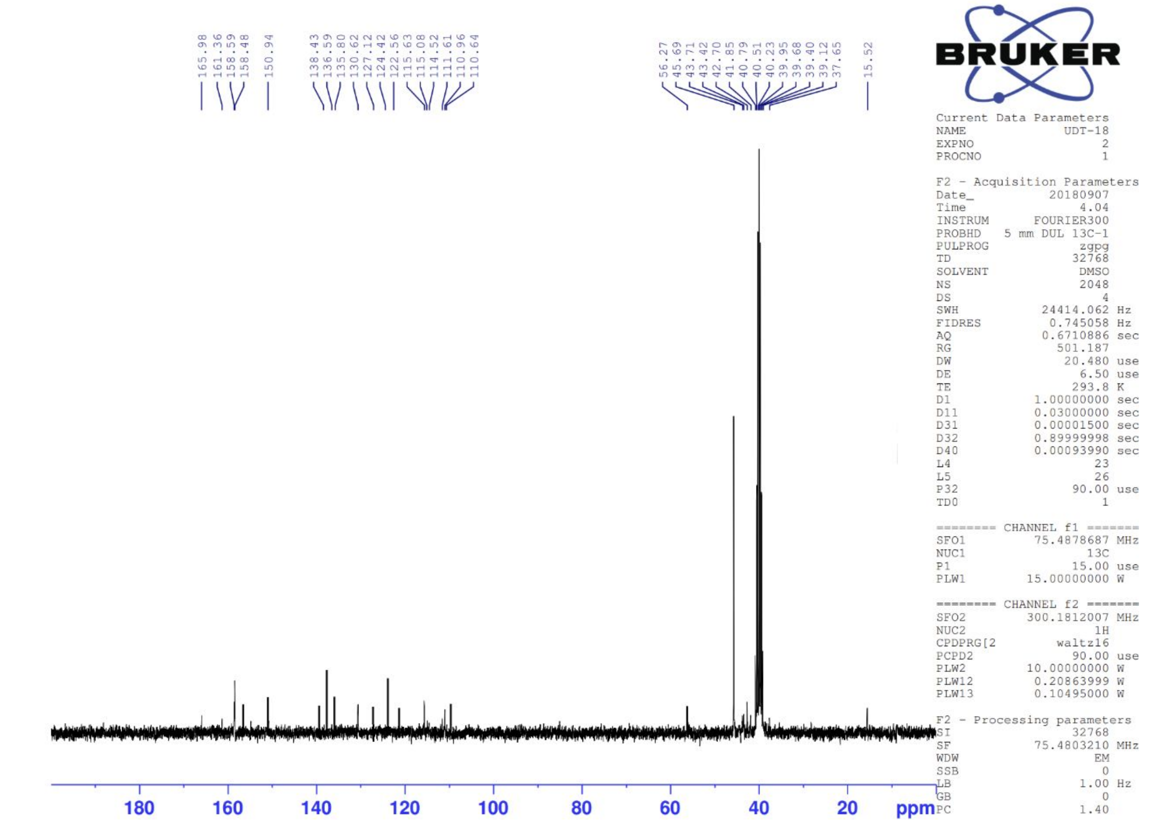

Figure S20. ^{13}C-NMR spectrum of compound **4h**

Data File: C:\LabSolutions\Data\Analiz\uac\UDT-18_4.lcd

Elmt	Val.	Min	Max	Elmt	Val.	Min	Max	Elmt	Val.	Min	Max	Elmt	Val.	Min	Max	Use Adduct
H	1	5	40	O	2	0	5	S	2	1	1	Ru	2	0	0	H
C	4	5	35	F	1	0	0	Cl	1	0	0	Pd	2	0	0	
N	3	0	9	P	3	0	0	Br	1	0	0	I	3	0	0	

Error Margin (ppm): 5
HC Ratio: unlimited
Max Isotopes: 3
MSn Iso RI (%): 10.00

DBE Range: 0.0 - 30.0
Apply N Rule: yes
Isotope RI (%): 1.00
MSn Logic Mode: AND

Electron Ions: both
Use MSn Info: yes
Isotope Res: 9000
Max Results: 150

Event#: 1 MS(E+) Ret. Time : 4.240 Scan# : 637

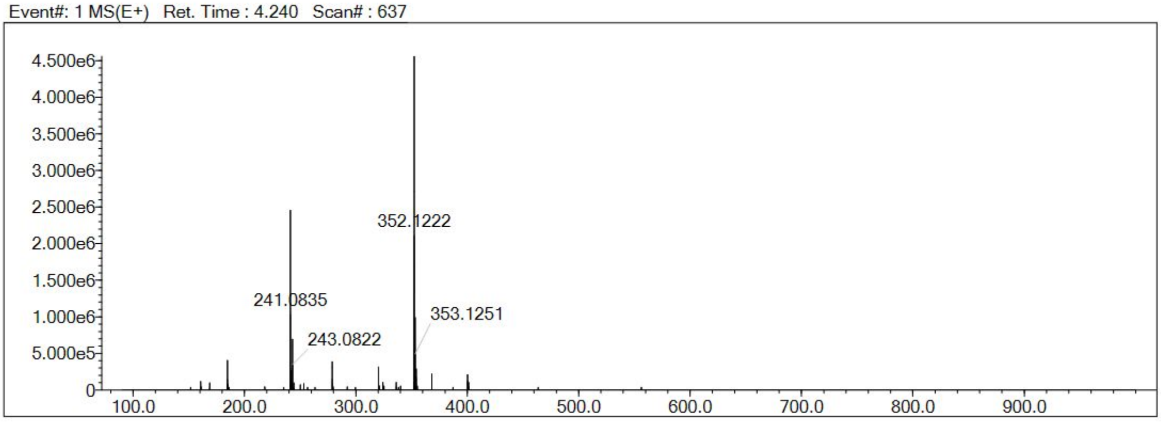

Measured region for 278.6146 m/z

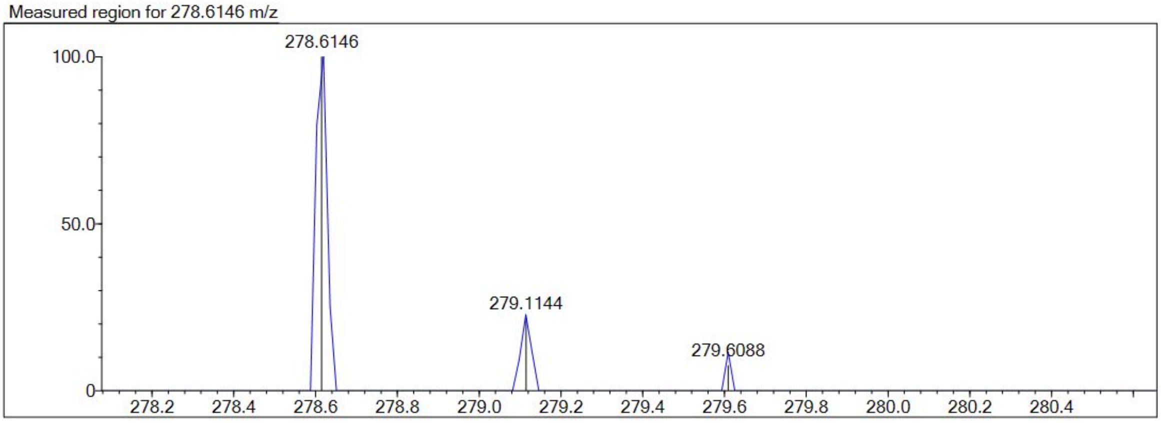

C28 H29 N9 O2 S [M+2H]2+ : Predicted region for 278.6155 m/z

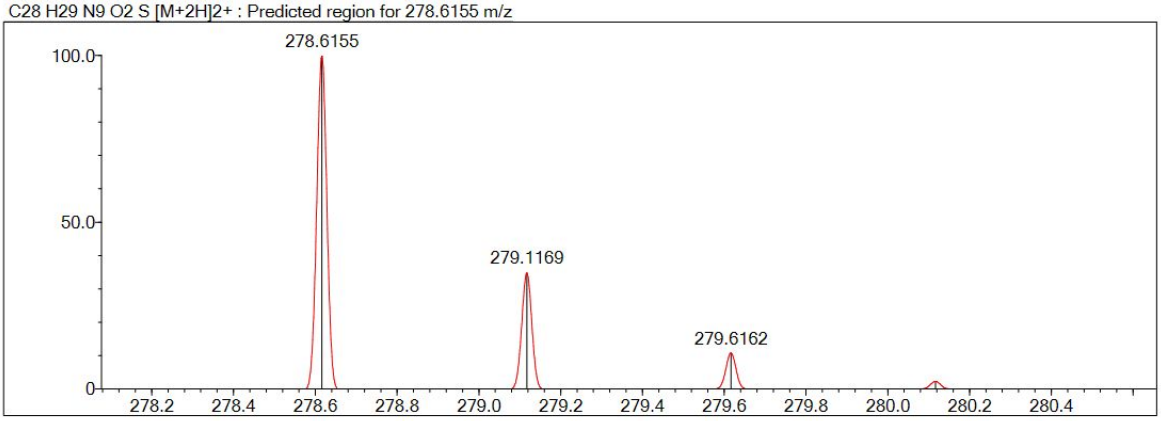

Rank	Score	Formula (M)	Ion	Meas. m/z	Pred. m/z	Df. (mDa)	Df. (ppm)	Iso	DBE
1	35.03	C28 H29 N9 O2 S	[M+2H]2+	278.6146	278.6155	-0.9	-3.23	37.09	19.0

Figure S21. Mass spectrum of compound **4h**

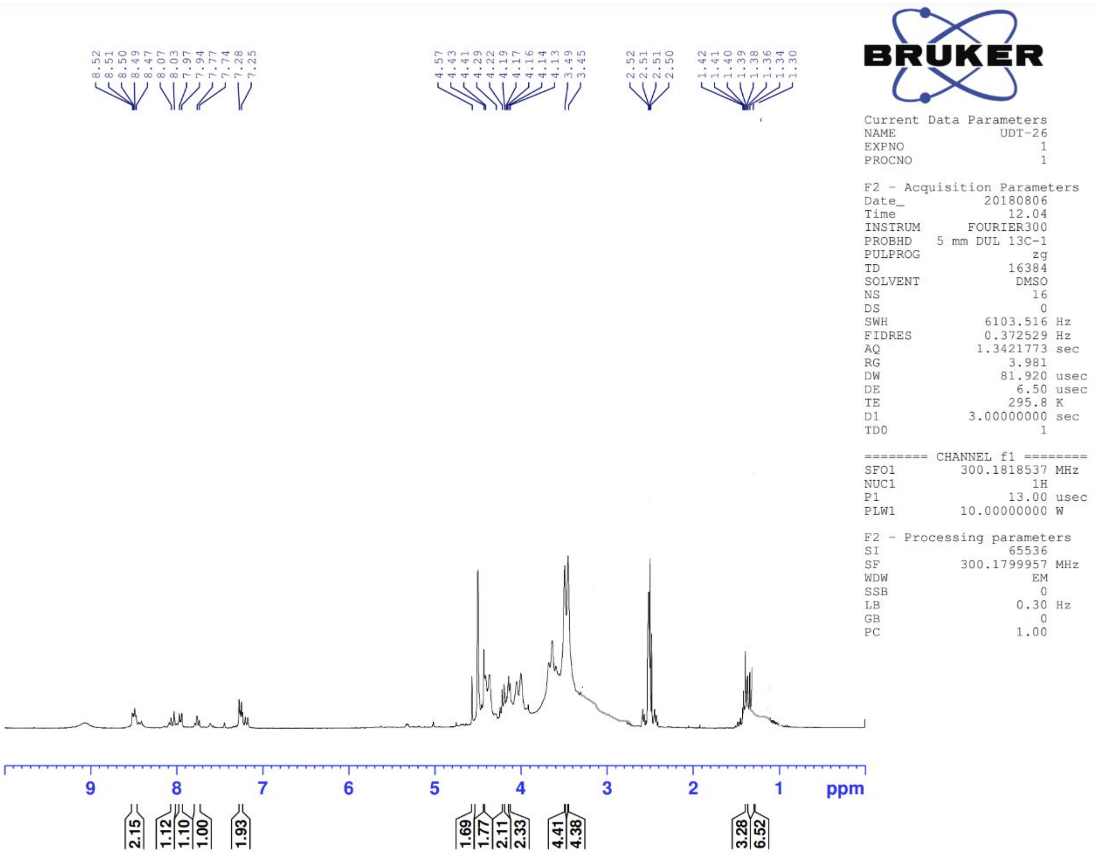

Figure S22. ^{1}H-NMR spectrum of compound **4i**

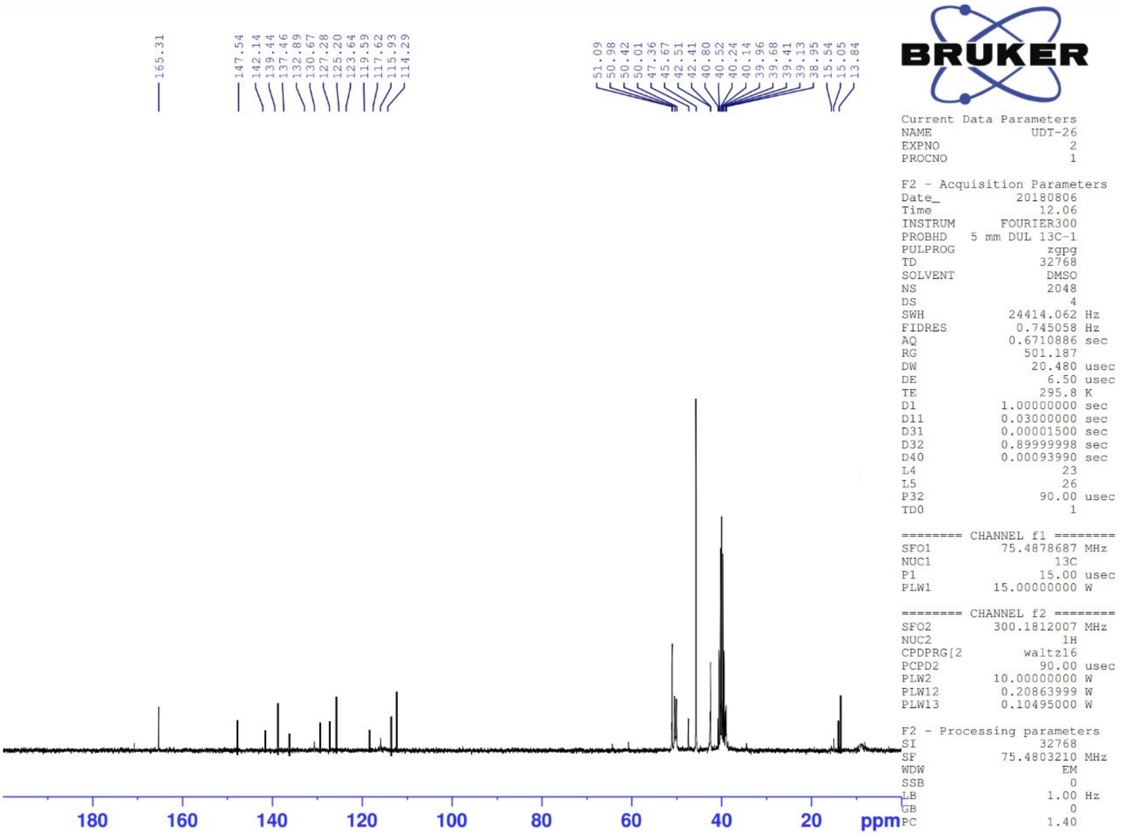

Figure S23. ^{13}C-NMR spectrum of compound **4i**

Data File: C:\LabSolutions\Data\Analiz\uac\UDT-26_16.lcd

Elmt	Val.	Min	Max	Elmt	Val.	Min	Max	Elmt	Val.	Min	Max	Elmt	Val.	Min	Max	Use Adduct
H	1	5	40	O	2	0	3	S	2	1	1	Ru	2	0	0	H
C	4	5	35	F	1	0	0	Cl	1	0	0	Pd	2	0	0	
N	3	2	10	P	3	0	0	Br	1	0	0	I	3	0	0	

Error Margin (ppm): 5
HC Ratio: unlimited
Max Isotopes: 3
MSn Iso RI (%): 10.00

DBE Range: 0.0 - 50.0
Apply N Rule: yes
Isotope RI (%): 1.00
MSn Logic Mode: AND

Electron Ions: both
Use MSn Info: yes
Isotope Res: 9000
Max Results: 150

Event#: 1 MS(E+) Ret. Time : 2.560 -> 2.787 Scan# : 385 -> 419

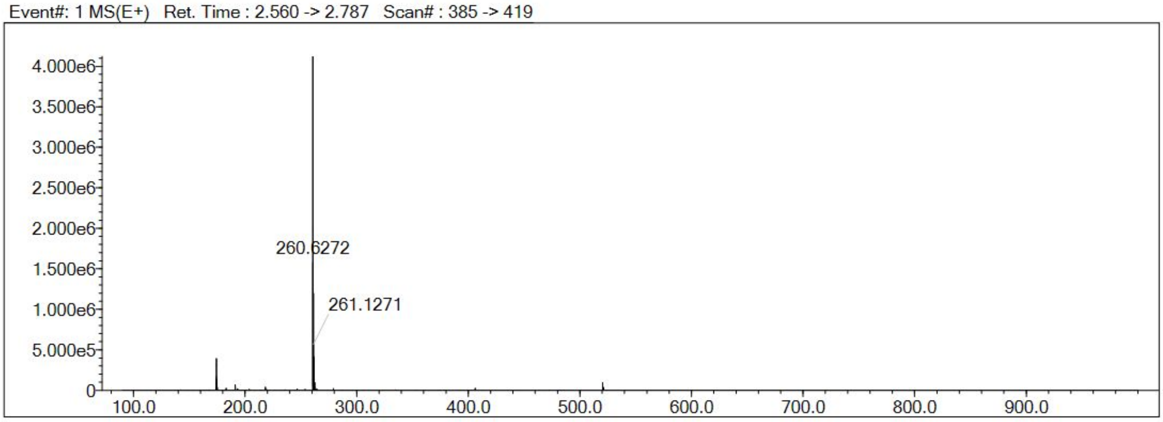

Measured region for 260.6272 m/z

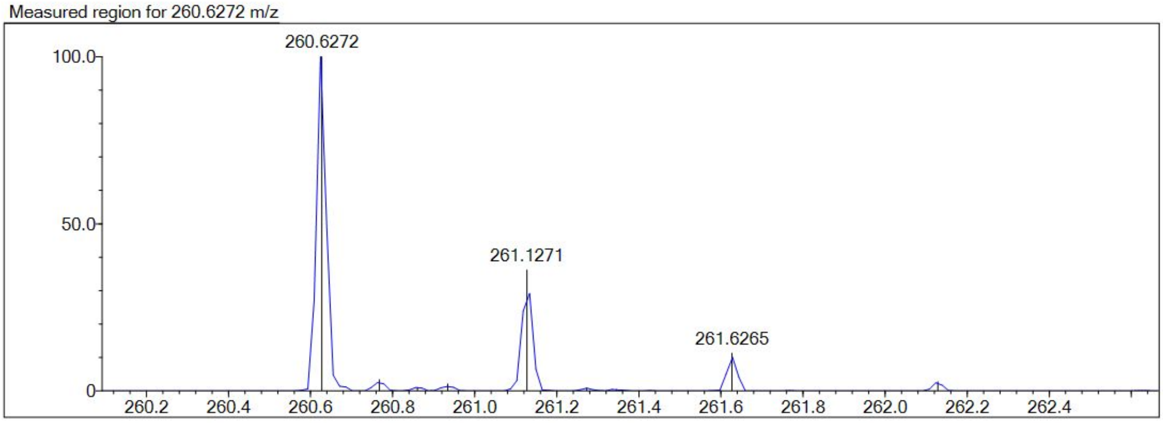

C27 H33 N7 O2 S [M+2H]2+ : Predicted region for 260.6281 m/z

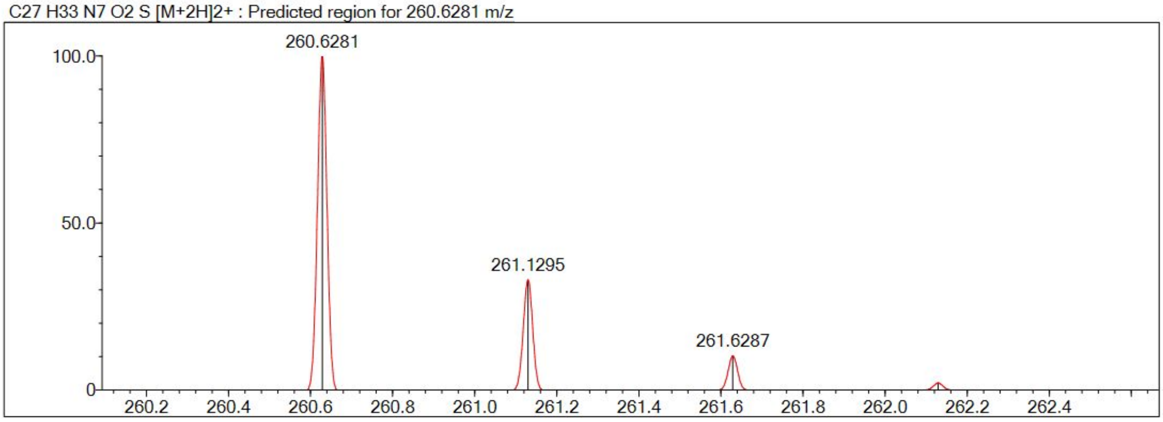

Rank	Score	Formula (M)	Ion	Meas. m/z	Pred. m/z	Df. (mDa)	Df. (ppm)	Iso	DBE
1	61.06	C27 H33 N7 O2 S	[M+2H]2+	260.6272	260.6281	-0.9	-3.45	65.04	15.0

Figure S24. Mass spectrum of compound **4i**

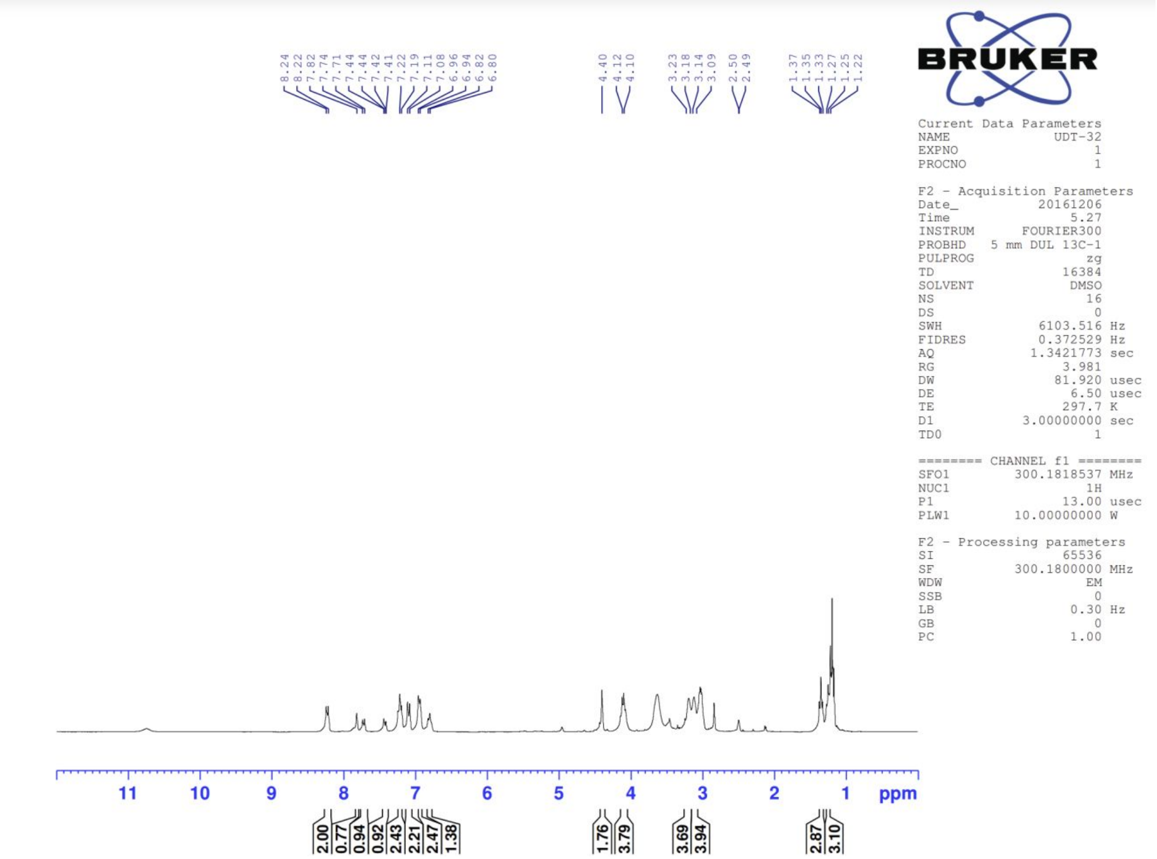

Figure S25. ^{1}H-NMR spectrum of compound **4j**

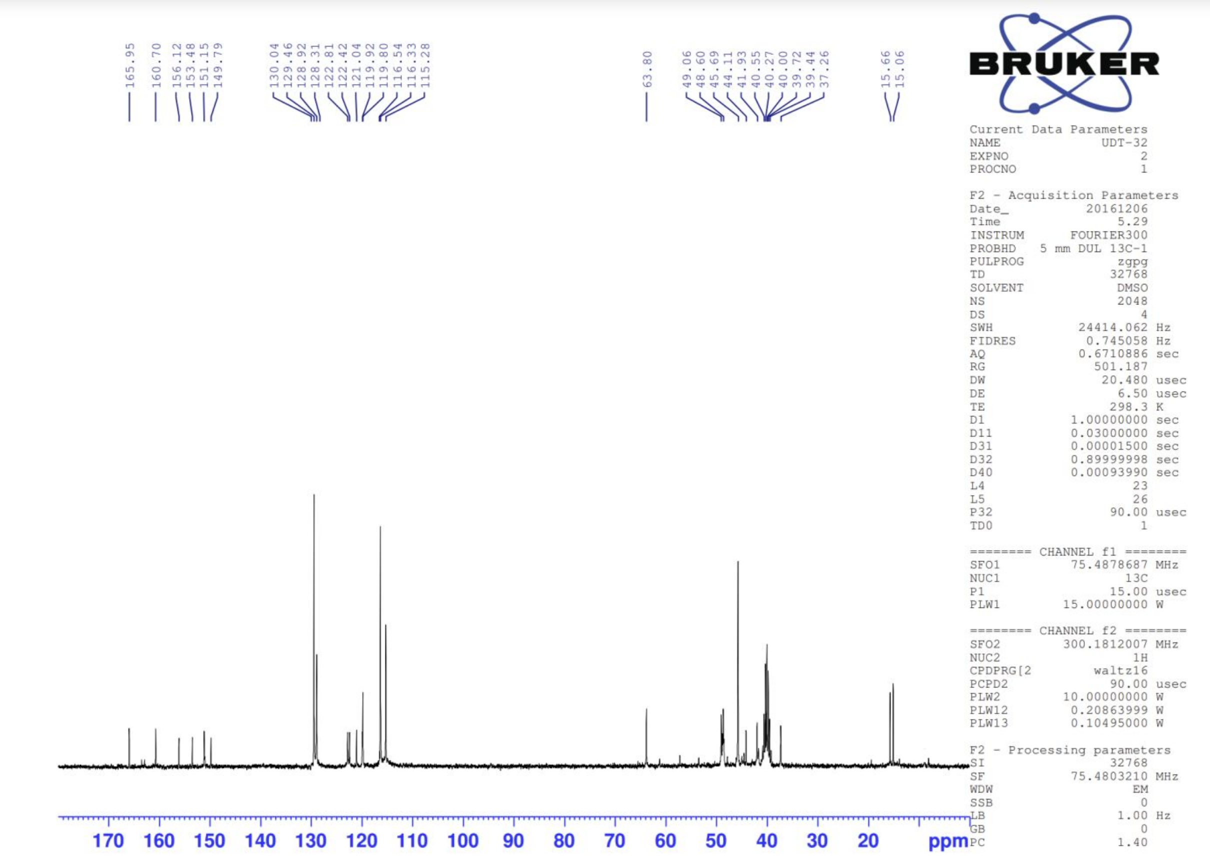

Figure S26. ^{13}C-NMR spectrum of compound **4j**

Data File: C:\LabSolutions\Data\Analiz\uac\UDT-32_21.lcd

Elmt	Val.	Min	Max	Elmt	Val.	Min	Max	Elmt	Val.	Min	Max	Elmt	Val.	Min	Max	Use Adduct
H	1	5	40	O	2	0	3	S	2	1	1	Ru	2	0	0	H
C	4	5	35	F	1	0	0	Cl	1	0	0	Pd	2	0	0	
N	3	2	10	P	3	0	0	Br	1	0	0	I	3	0	0	

Error Margin (ppm):	5	DBE Range:	0.0 - 60.0	Electron Ions:	both
HC Ratio:	unlimited	Apply N Rule:	yes	Use MSn Info:	yes
Max Isotopes:	3	Isotope RI (%):	1.00	Isotope Res:	9000
MSn Iso RI (%):	10.00	MSn Logic Mode:	AND	Max Results:	150

Event#: 1 MS(E+) Ret. Time : 5.080 Scan# : 763

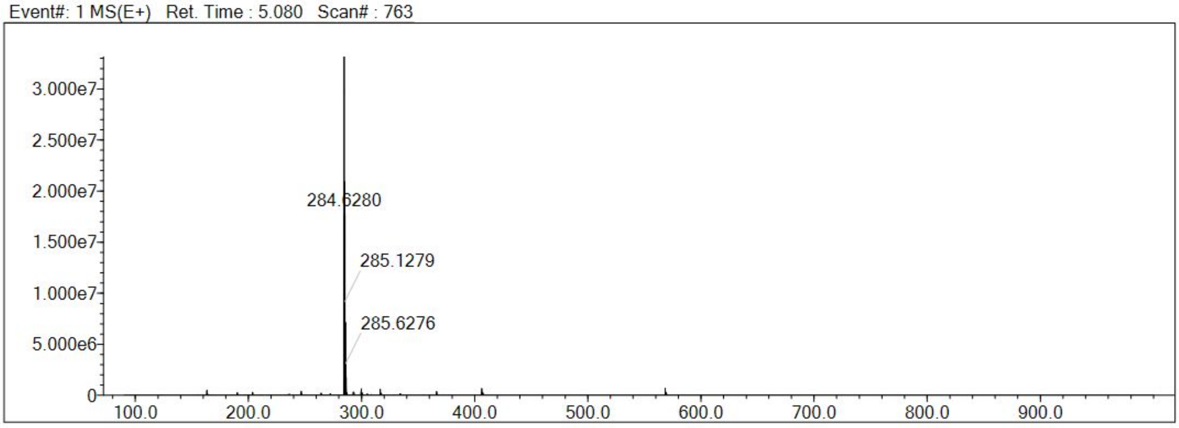

Measured region for 284.6280 m/z

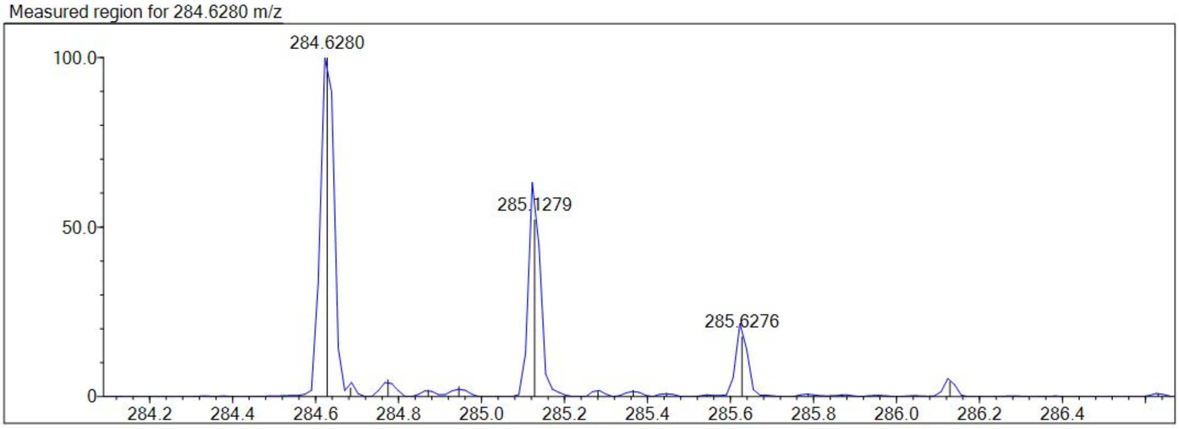

C31 H33 N7 O2 S [M+2H]2+ : Predicted region for 284.6281 m/z

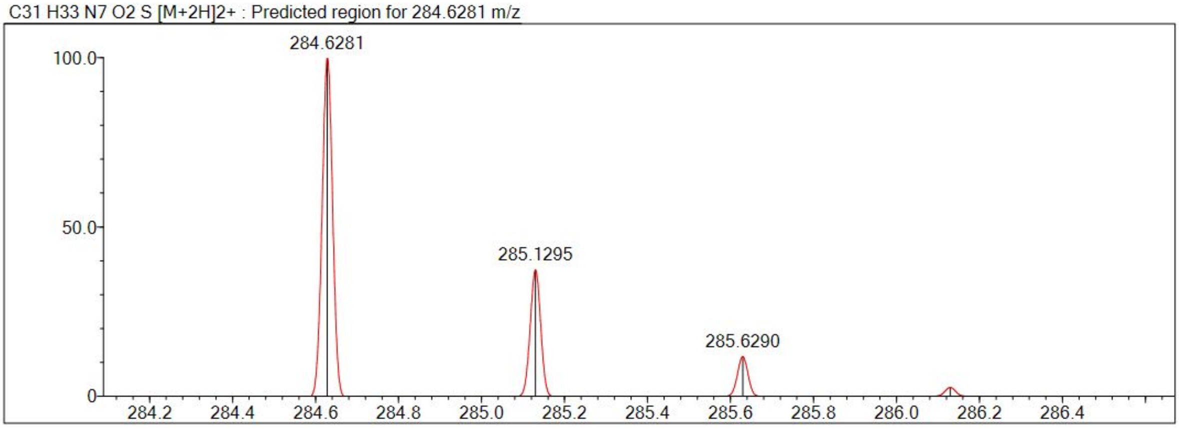

Rank	Score	Formula (M)	Ion	Meas. m/z	Pred. m/z	Df. (mDa)	Df. (ppm)	Iso	DBE
1	54.34	C31 H33 N7 O2 S	[M+2H]2+	284.6280	284.6281	-0.1	-0.35	54.34	19.0

Figure S27. Mass spectrum of compound **4j**

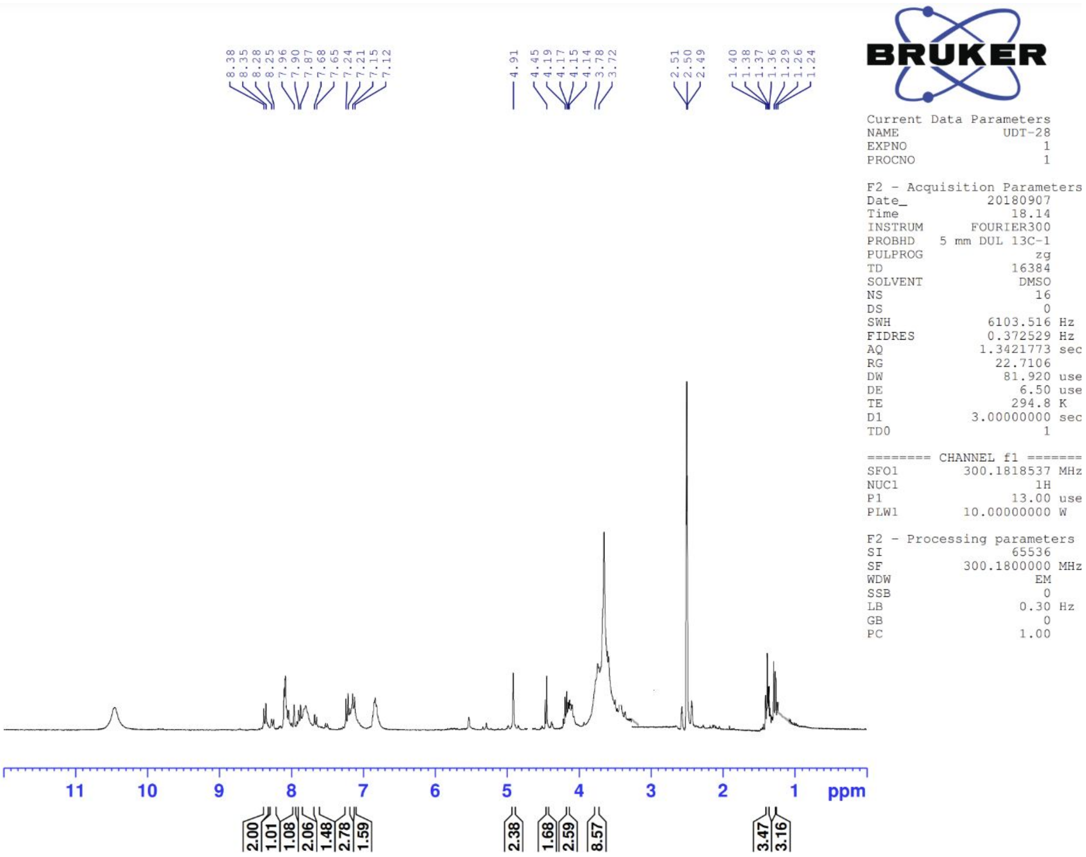

Figure S28. ^{1}H-NMR spectrum of compound **4k**

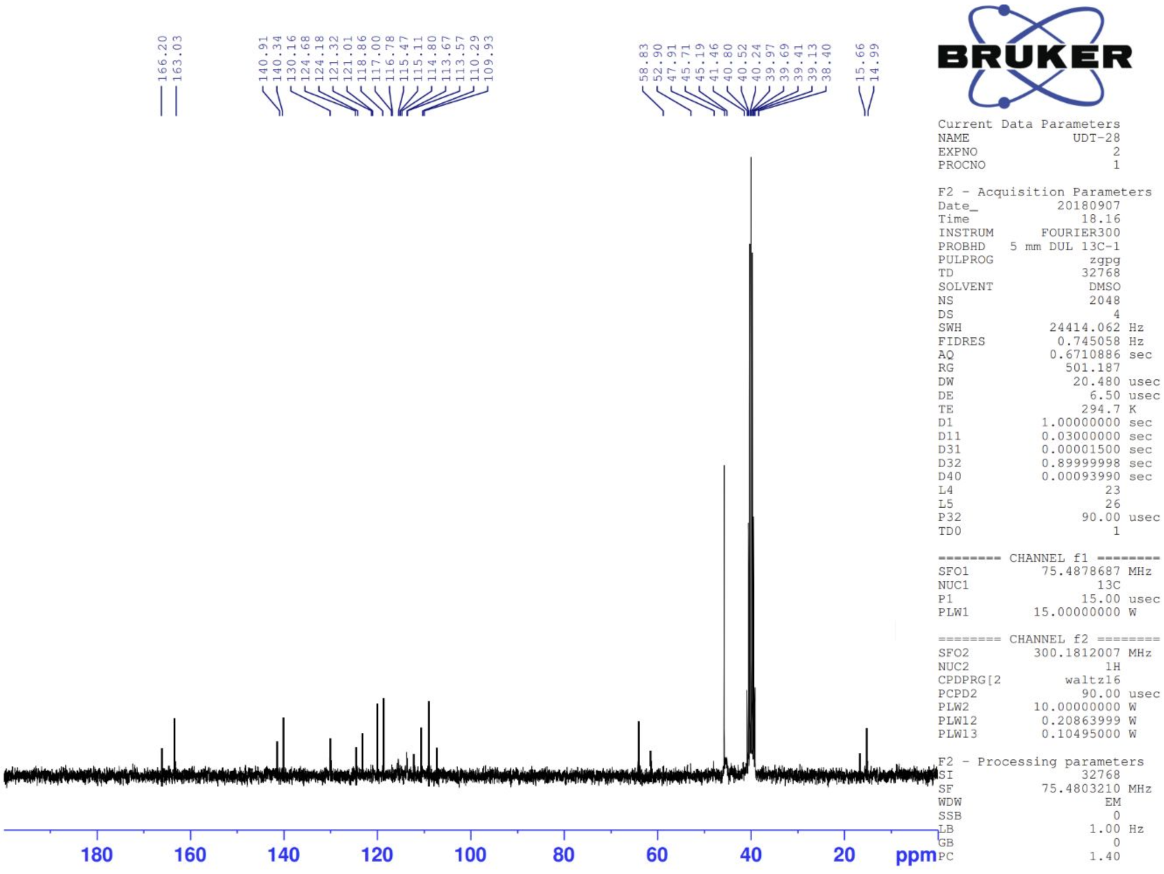

Figure S29. ^{13}C-NMR spectrum of compound **4k**

Formula Predictor Report - UDT-28_18.lcd

Data File: C:\LabSolutions\Data\Analiz\uac\UDT-28_18.lcd

Elmt	Val.	Min	Max	Elmt	Val.	Min	Max	Elmt	Val.	Min	Max	Elmt	Val.	Min	Max	Use Adduct
H	1	5	40	O	2	0	3	S	2	1	1	Ru	2	0	0	H
C	4	5	35	F	1	0	0	Cl	1	0	0	Pd	2	0	0	
N	3	2	10	P	3	0	0	Br	1	0	0	I	3	0	0	

Error Margin (ppm):	5	DBE Range:	0.0 - 50.0	Electron Ions:	both
HC Ratio:	unlimited	Apply N Rule:	yes	Use MSn Info:	yes
Max Isotopes:	3	Isotope RI (%):	1.00	Isotope Res:	9000
MSn Iso RI (%):	10.00	MSn Logic Mode:	AND	Max Results:	150

Event#: 1 MS(E+) Ret. Time : 3.400 -> 3.693 Scan# : 511 -> 555

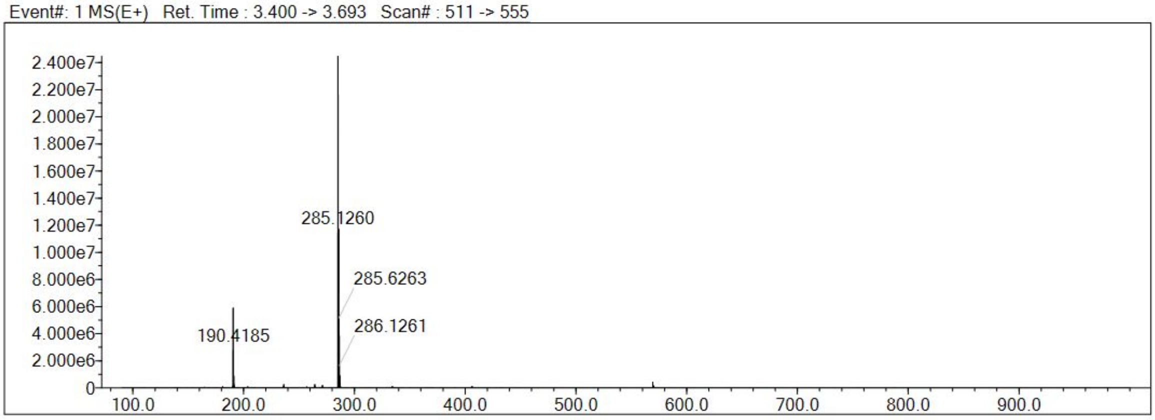

Measured region for 285.1260 m/z

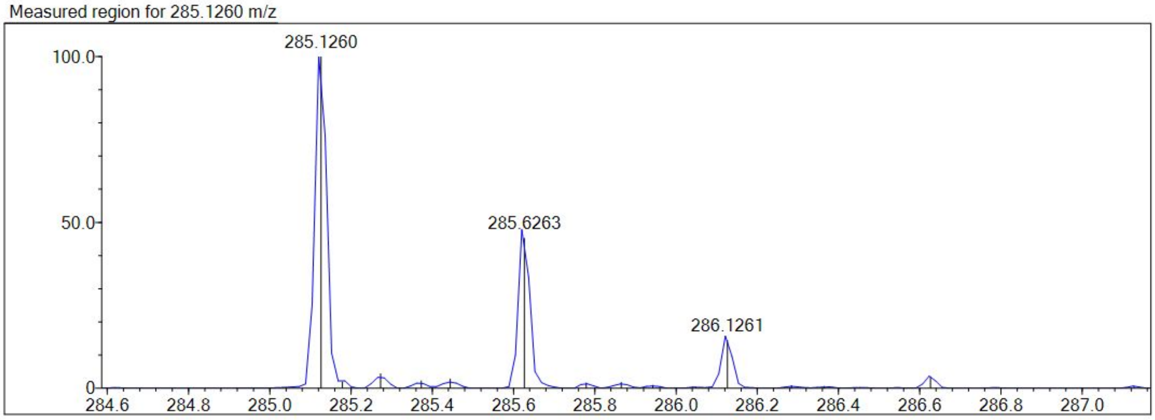

C30 H32 N8 O2 S [M+2H]2+ : Predicted region for 285.1257 m/z

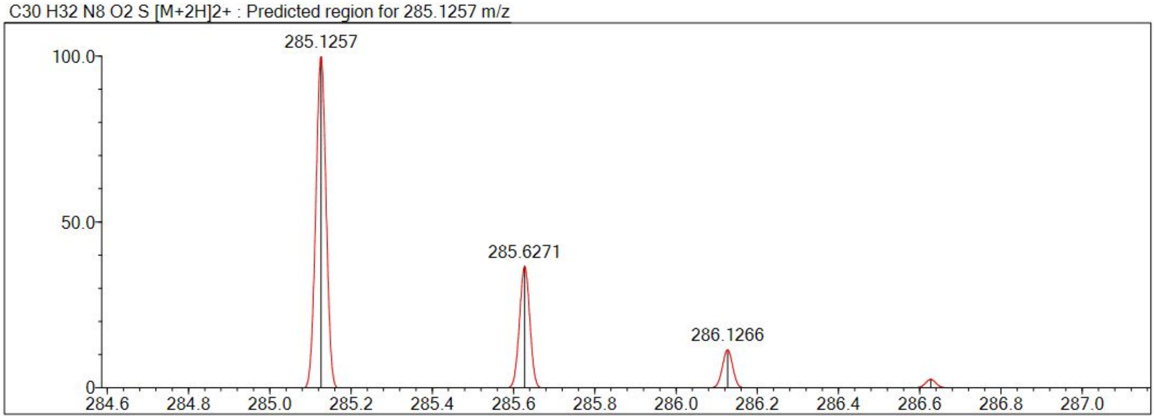

Rank	Score	Formula (M)	Ion	Meas. m/z	Pred. m/z	Df. (mDa)	Df. (ppm)	Iso	DBE
1	71.15	C30 H32 N8 O2 S	[M+2H]2+	285.1260	285.1257	0.3	1.05	71.24	19.0

Figure S30. Mass spectrum of compound **4k**

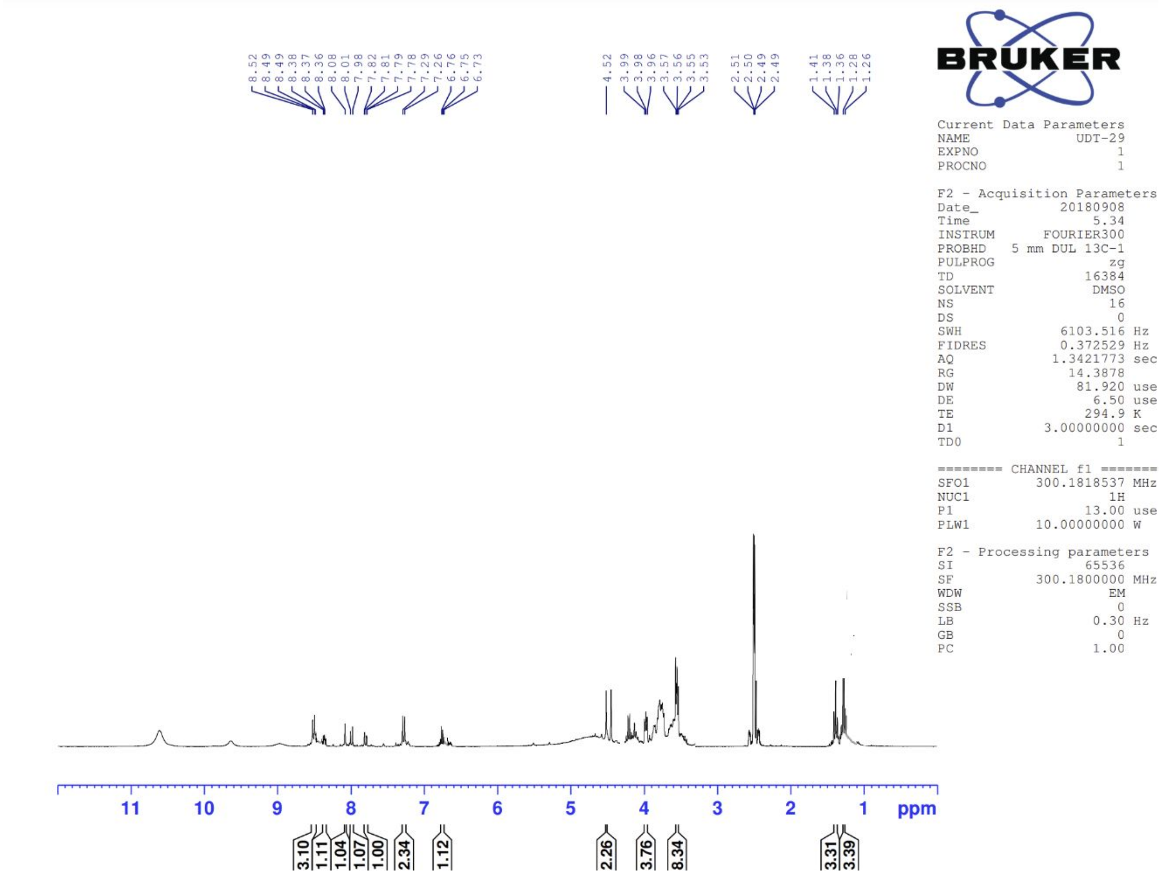

Figure S31. ^{1}H-NMR spectrum of compound **4l**

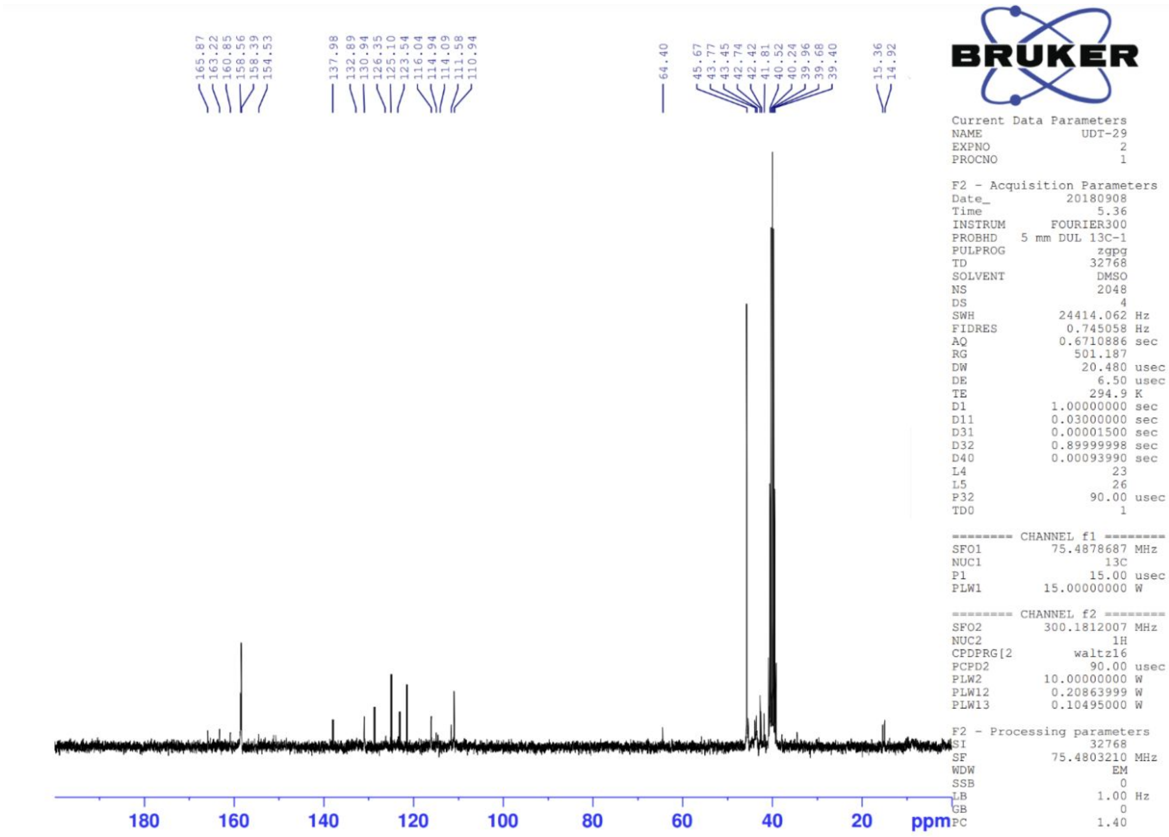

Figure S32. ^{13}C-NMR spectrum of compound **4l**

Data File: C:\LabSolutions\Data\Analiz\uac\UDT-2_2.lcd

Elmt	Val.	Min	Max	Elmt	Val.	Min	Max	Elmt	Val.	Min	Max	Elmt	Val.	Min	Max	Use Adduct
H	1	5	35	O	2	0	5	S	2	1	1	Ru	2	0	0	H
C	4	5	35	F	1	0	0	Cl	1	0	0	Pd	2	0	0	
N	3	0	8	P	3	0	0	Br	1	0	0	I	3	0	0	

Error Margin (ppm):	5	DBE Range:	0.0 - 20.0	Electron Ions:	both
HC Ratio:	unlimited	Apply N Rule:	yes	Use MSn Info:	yes
Max Isotopes:	3	Isotope RI (%):	1.00	Isotope Res:	9000
MSn Iso RI (%):	10.00	MSn Logic Mode:	AND	Max Results:	150

Event#: 1 MS(E+) Ret. Time : 4.627 Scan# : 695

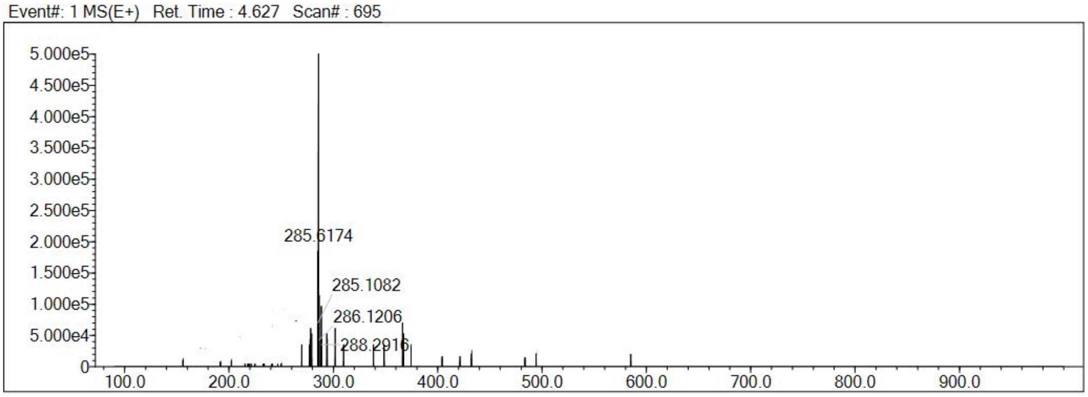

Measured region for 285.6174 m/z

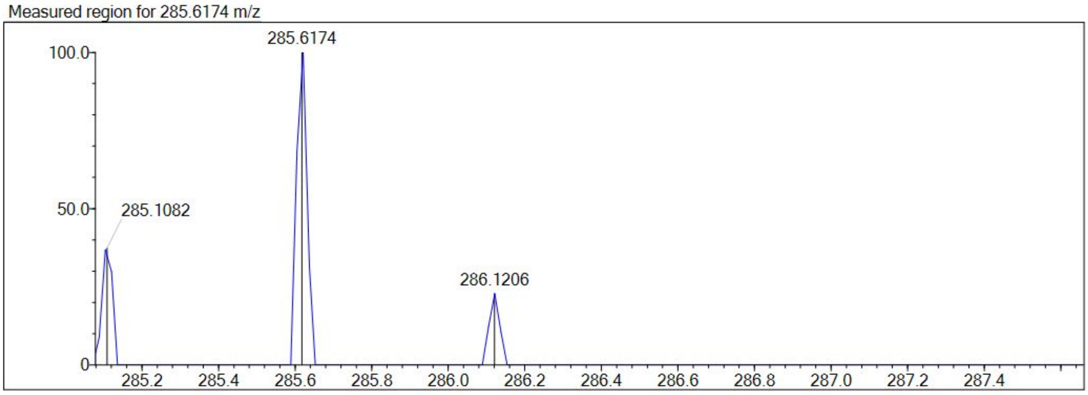

C30 H31 N7 O3 S [M+2H]2+ : Predicted region for 285.6177 m/z

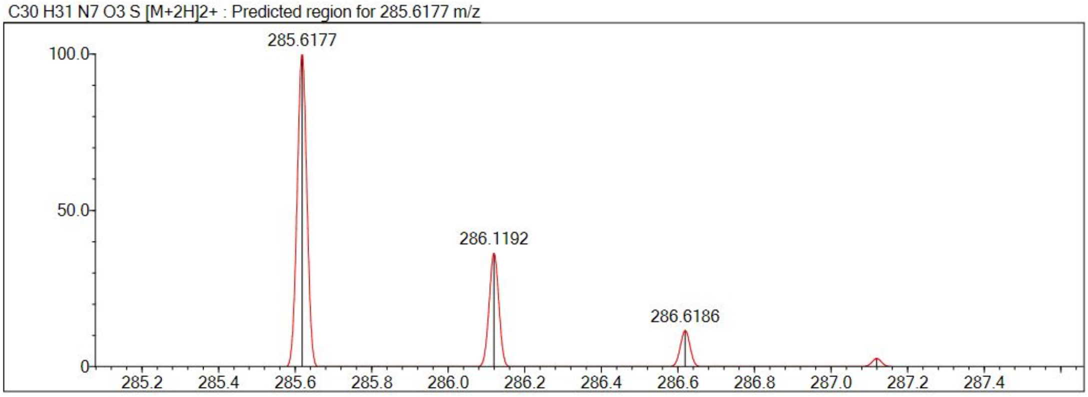

Figure S31. Mass spectrum of compound **4l**
